# Photolysis of cell-permeant caged inositol pyrophosphates controls oscillations of cytosolic calcium in a β-cell line†
†Electronic supplementary information (ESI) available. See DOI: 10.1039/c8sc03479f


**DOI:** 10.1039/c8sc03479f

**Published:** 2019-01-10

**Authors:** S. Hauke, A. K. Dutta, V. B. Eisenbeis, D. Bezold, T. Bittner, C. Wittwer, D. Thakor, I. Pavlovic, C. Schultz, H. J. Jessen

**Affiliations:** a EMBL, Heidelberg , 69117 Heidelberg , Germany . Email: Schultz@embl.de; b University of Freiburg , Institute of Organic Chemistry , 79104 Freiburg , Germany . Email: Henning.jessen@oc.uni-freiburg.de; c OHSU , Dept. Physiology & Pharmacology , Portland , OR , USA . Email: Schulcar@ohsu.edu

## Abstract

Among many cellular functions, inositol pyrophosphates (PP-InsPs) are metabolic messengers involved in the regulation of glucose uptake, insulin sensitivity, and weight gain. However, their mechanisms of action are still poorly understood. So far, the influence of PP-InsPs on cellular metabolism has been studied by overexpression or knockout/inhibition of relevant metabolizing kinases (IP6Ks, PPIP5Ks). These approaches are, *inter alia*, limited by time-resolution and potential compensation mechanisms. Here, we describe the synthesis of cell-permeant caged PP-InsPs as tools to rapidly modulate intracellular levels of defined isomers of PP-InsPs in a genetically non-perturbed cellular environment. We show that caged prometabolites readily enter live cells where they are enzymatically converted into still inactive, metabolically stable, photocaged PP-InsPs. Upon light-triggered release of 5-PP-InsP_5_, the major cellular inositol pyrophosphate, oscillations of intracellular Ca^2+^ levels in MIN6 cells were transiently reduced to spontaneously recover again. In contrast, uncaging of 1-PP-InsP_5_, a minor cellular isomer, was without effect. These results provide evidence that PP-InsPs play an active role in regulating [Ca^2+^]_i_ oscillations, a key element in triggering exocytosis and secretion in β-cells.

## Abstract 2

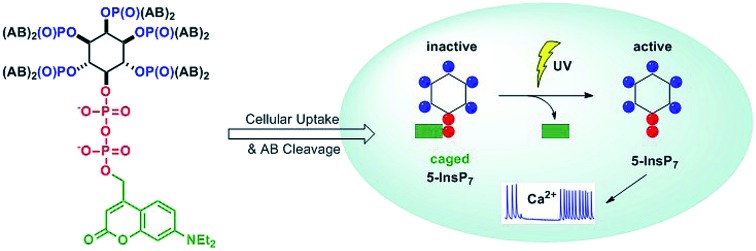
β-Cells respond directly to the intracellular photochemical release of caged inositol pyrophosphate isomers with modulations of oscillations in cytosolic Ca^2+^.

## Introduction

**Fig. 1:**
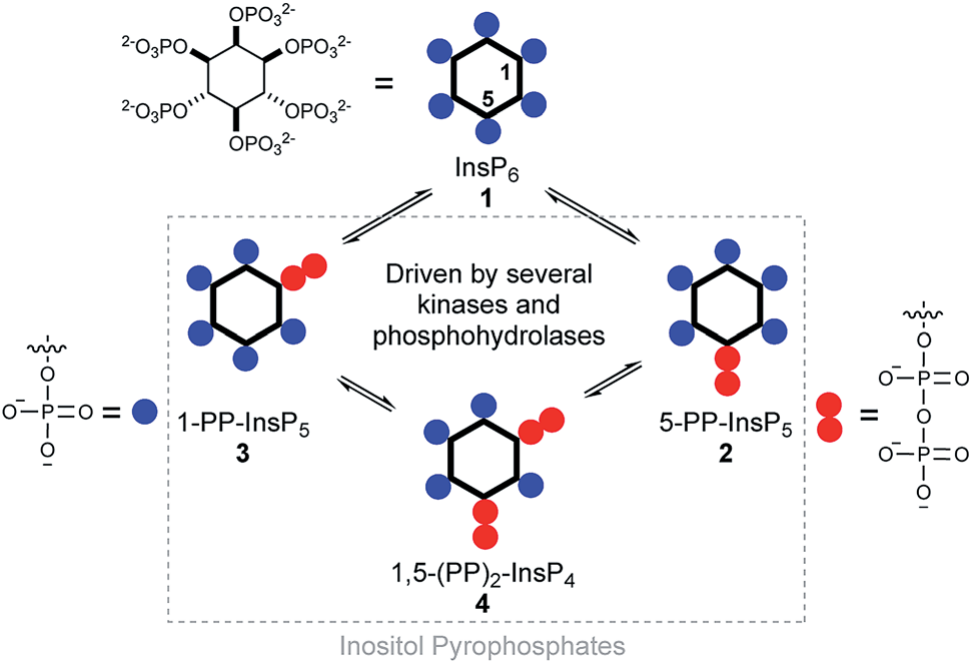
Structure of inositol pyrophosphates and schematic, simplified overview of their metabolic turnover.

Diphosphoinositol polyphosphates (hereafter called inositol pyrophosphates or PP-InsPs) are hyperphosphorylated molecules derived from *myo*-inositol hexakisphosphate (InsP_6_) 1.^1^,^2^ 5-PP-InsP_5_ 2 is the most abundant representative in mammalian cells, but 1-PP-InsP_5_ 3 and 1,5-(PP)_2_-InsP_4_ 4 are also present and are interconverted by enzymatic action (Fig. 1). Many important biological functions in eukaryotes have been associated with PP-InsPs.^3^–^5^ An emerging concept classifies PP-InsPs as ‘metabolic messengers’ that monitor cellular energy and phosphate homeostasis.^6^–^14^ Mice lacking inositol hexakisphosphate kinase 1 (IP6K1 KO), one of the three mammalian InsP_6_ kinases that generate 5-PP-InsP_5_ 2 from InsP_6_ 1, exhibit reduced body weight, do not gain weight on a high-fat diet and are insulin hypersensitive while having reduced levels of circulating insulin.^15^,^16^ Recently, the pan-IP6K inhibitor *N*^2^-(*m*-(trifluoromethyl)benzyl)-*N*^6^-(*p*-nitrobenzyl)purine (TNP)^17^ was shown to protect mildly obese mice from the progression of diet-induced obesity.^18^ Therefore, IP6K1 was suggested as a potential drug target for the treatment of diabetes and obesity.^18^ In this context, it has been reported that extracellularly applied InsP_6_ and 5-PP-InsP_5_ trigger the release of insulin from the readily releasable pool of permeabilized pancreatic β-cells, measured indirectly using patch clamp technology.^19^,^20^

Insulin release from pancreatic β-cells in response to changes in blood-glucose levels is the hallmark of β-cell physiology. Glucose stimulated insulin secretion involves the glucose metabolism-induced rise of the intracellular ATP/ADP ratio. Increased ATP-levels act by inhibiting ATP-sensitive K^+^ (K_ATP_) channels, which results in decreased K^+^-influx, membrane depolarization and subsequent voltage-gated Ca^2+^-influx. Even though an involvement of PP-InsPs in the regulation of oscillations of cytosolic Ca^2+^ levels ([Ca^2+^]_i_ oscillations) seems reasonable due to their impact on insulin secretion in β-cells, their immediate effect on [Ca^2+^]_i_ oscillations has not been studied before.

Extracellular application of non-caged metabolites comes with the drawback of unpredictable concentration gradients across the plasma membrane of live cells, especially when studying charged metabolites. Since chemical or electrophysiological permeabilization of cells causes significant perturbation, even more so when the biological effect is driven by changes in the plasma membrane potential, less invasive technologies are in high demand. The spontaneous increase of second messenger levels using photolysis is such a technology.^21^ The photolabile cage masks the biomolecule to prevent metabolism and target proteins binding until defined light-mediated release of the messenger with high spatiotemporal control. However, if the second messenger under investigation bears significant charge, care must be taken in order to deliver it across the plasma membrane.^22^ Here, a typical approach is masking the negatively charged phosphates by alkylation with acetoxyalkyloxy esters.^23^–^25^

Recently, the cellular delivery of 5-PP-InsP_5_ using acetoxybenzylesters (AB) has been reported.^26^ The latter are quickly degraded inside live cells to release the unmasked phosphate ester. Delivery of a photocaged 5-PP-InsP_5_ using this prometabolite technology has never been achieved before but would additionally facilitate temporal and spatial control over the release of the active messengers and thus avoid concentration gradients. To date, this has not been achieved for any member of the inositol pyrophosphate family. Controlled intracellular release of non-symmetric PP-InsPs, such as 1-PP-InsP_5_, also remains an unsolved problem that it now addressed in this publication. In contrast, the delivery of a symmetric caged 5-PP-InsP_5_ using non-covalent association with a guanidinium-rich molecular transporter was recently reported and used to demonstrate the effect of 5-PP-InsP_5_ on the subcellular localization of the PH domain of Akt in living HeLa cells.^27^

Here, we demonstrate the efficient loading of pancreatic mouse insulinoma 6 (MIN6) cells^28^ with the photocaged PP-InsP isomers 1-PP-InsP_5_, 3-PP-InsP_5_, and 5-PP-InsP_5_, without the need for cell permeabilization or use of additives. The simple incubation of cells with photocaged prometabolites is sufficient. Moreover, we provide evidence that an increase of intracellular 5-PP-InsP_5_ concentrations profoundly impacts β-cell activity ([Ca^2+^]_i_ oscillations), whereas 1-PP-InsP_5_ is inactive.

## Results and discussion

**Scheme 1:**
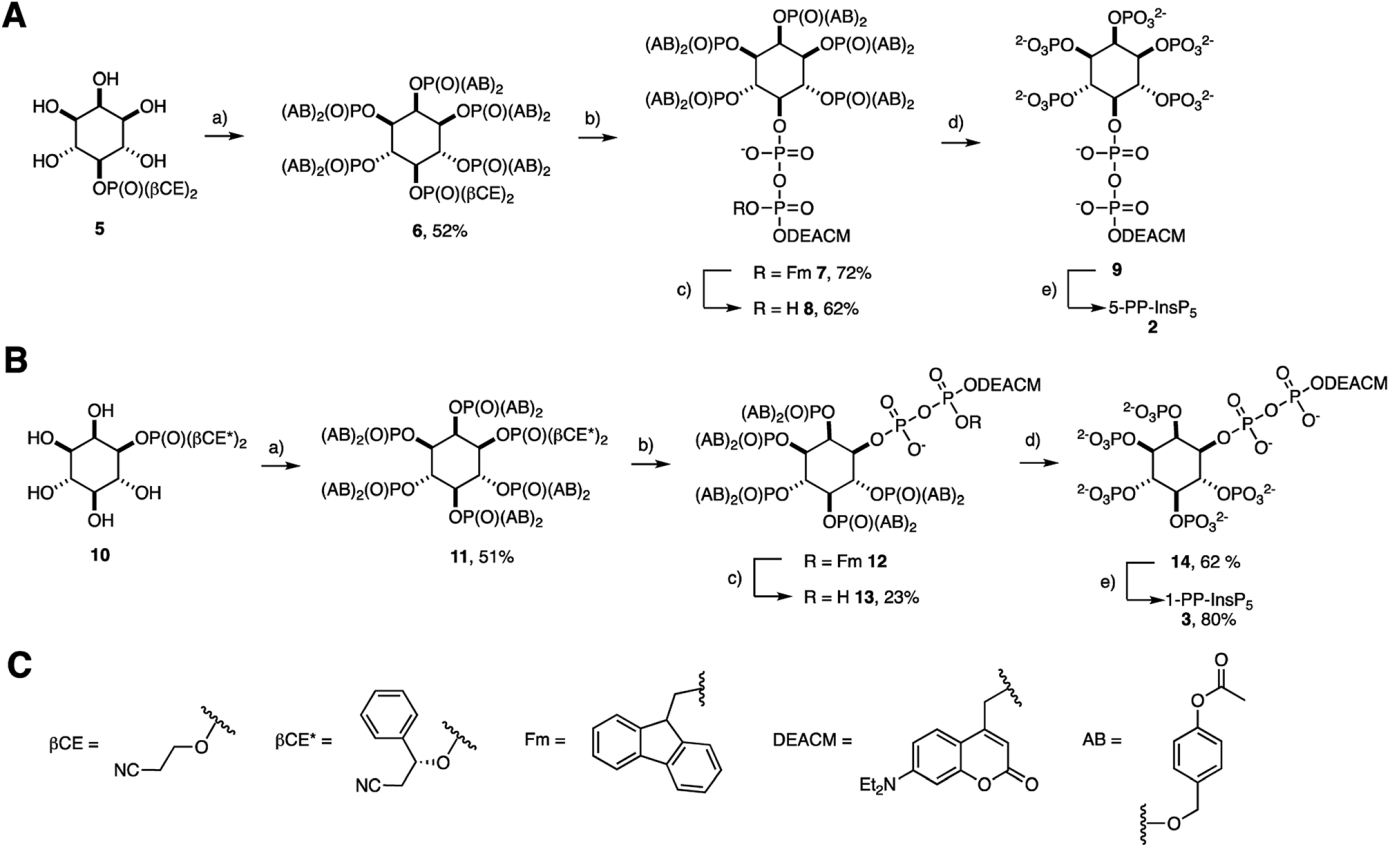
Synthesis of (AB)_10_ DEACM protected target molecules 8, 13 and their conversion into reference compounds 9, 14, 2, and 3. (A) (a) (*i*pr_2_N)P(AB)_2_, ETT, DMF, then *m*CPBA; (b) DBU, BSTFA, MeCN then TFA, MeOH, then (*i*pr_2_N)P(Fm)(DEACM), then *m*CPBA; (c) piperidine (1.2 eq.), DMF; (d) chemically: 33% piperidine in DMF; or enzymatically: with MIN6 cell extract; (e) *hν*; (B) (a) (*i*pr_2_N)P(AB)_2_, ETT, DMF, then *m*CPBA; (b) DBU, BSTFA, MeCN then TFA, MeOH, then (*i*pr_2_N)P(Fm)(DEACM), then *m*CPBA; (c) piperidine, DMF; (d) chemically: 33% piperidine in DMF; or enzymatically: with MIN6 cell extract; (e) *hν*; (C) structures of the protecting groups. Abbreviations: ETT 5-ethylthio tetrazole, DMF *N*,*N*-dimethylformamide, *m*CPBA *meta*-chloroperoxybenzoic acid, DBU 1,8-diazabicyclo[5.4.0]undec-7-ene, BSTFA *N*,*O*-bis(trimethylsilyl)trifluoroacetamide, TFA trifluoroacetic acid.

The preparation of acetoxybenzyl (AB) prometabolites of [7-(diethylamino)coumarin-4-yl]methyl (DEACM) caged PP-InsPs is shown in Scheme 1(A and B). Synthesis of (AB)_10_-DEACM 5-PP-InsP_5_ 8 started from pentanol 5.^29^ The AB protected phosphates were installed using P(iii) chemistry, followed by oxidation to give hexakisphosphate 6 in 52% yield.^30^ Next, the β-cyanoethyl (*β*-CE) groups were replaced by TMS esters, which were methanolized, followed by generation of a P(iii)–P(v) anhydride and oxidation to the protected diphosphate 7 (72% yield) in a one-flask procedure.^26^,^29^,^31^–^33^ The P(iii)–P(v) anhydride was generated with a fluorenylmethyl (Fm) and DEACM modified P-amidite.^24^ In the final step, the Fm group was removed with piperidine (1.2 eq.) to release the caged prometabolite (AB)_10_-DEACM 5-PP-InsP_5_ 8 in 62% yield. For the prometabolite approach, it is vital that the β-phosphate only bears one protecting group (in this case: DEACM) to avoid unwanted side-product formation in the enzymatic cleavage.^26^ Overall, a yield of 23% was achieved for the transformation of 5 to 8. Cleavage of the AB groups leads to the release of DEACM-caged 5-PP-InsP_5_ 9,^27^ and irradiation releases the active messenger 5-PP-InsP_5_ 2 (Scheme 1A and B).

The synthesis of 1-PP-InsP_5_ prometabolite 13 required desymmetrization of *myo*-inositol, which was achieved by asymmetric phosphorylation. This strategy enables the installation of a protected phosphate in the 1- and 3-positions of *myo*-inositol, such as in phosphate ester 10.^29^,^31^ The chiral auxiliary *β*CE* used in this strategy resembles the achiral *β*CE protecting group used in approach A (Scheme 1). This strategy enabled the same synthetic approach to generate (AB)_10_-DEACM 1-PP-InsP_5_ 13 in enantiomerically pure form (Scheme 1B) as well as its enantiomer (AB)_10_-DEACM 3-PP-InsP_5_*ent*-13 (synthesis and data in the ESI†). The overall yield for the synthesis of 13 starting from 10 was 12% after purification by preparative RP-HPLC using evaporative light-scattering detection to avoid cleavage of the photocage. In order to obtain DEACM 1-PP-InsP_5_ (14) without AB groups as a reference, the AB groups were removed chemically with 33% piperidine in DMF in 68% yield. Furthermore, DEACM was removed by irradiation in a photoreactor releasing 1-PP-InsP_5_ 3 in high purity after ion exchange and precipitation in 80% yield.

**Fig. 2:**
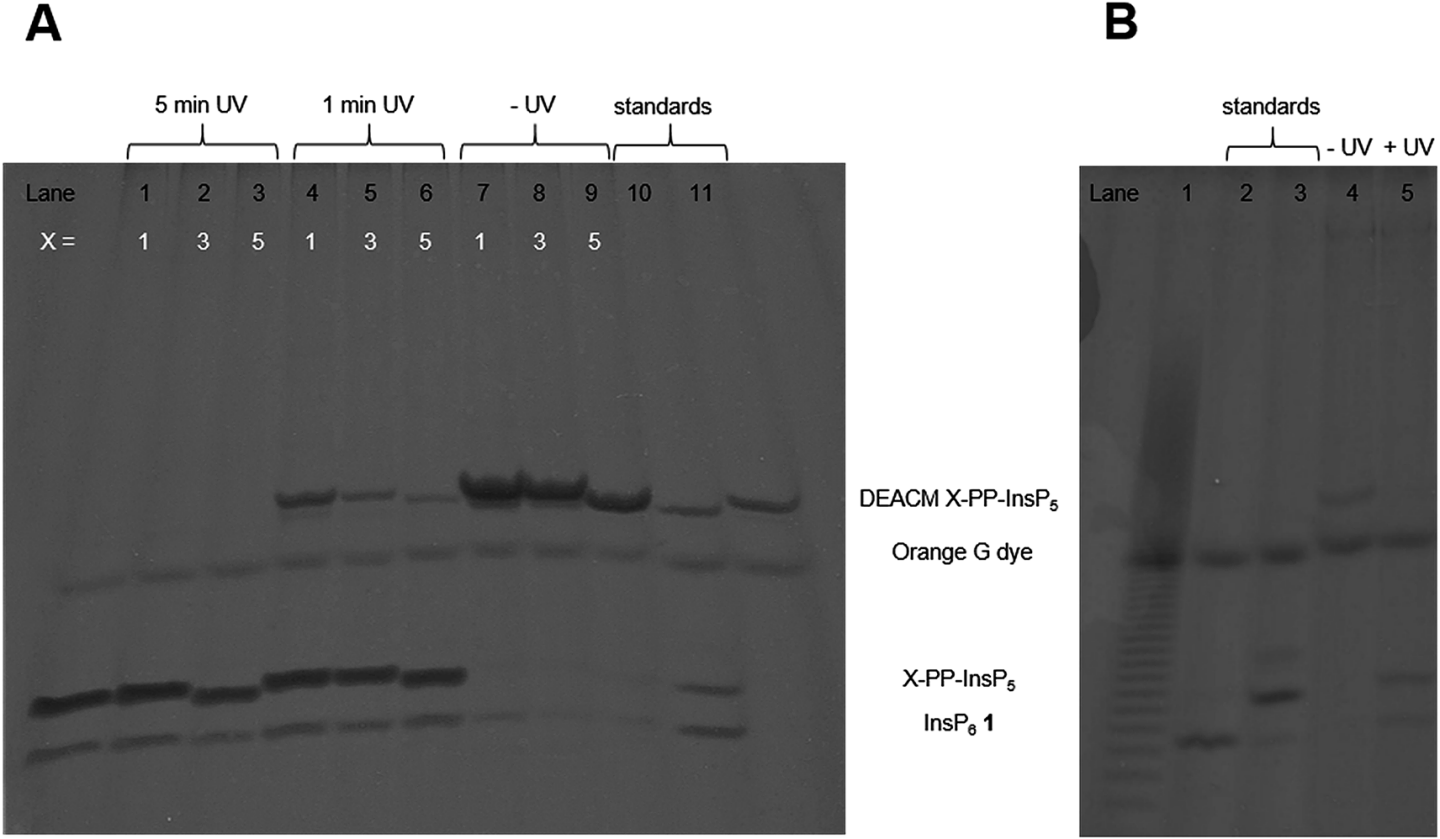
PAGE analysis of (AB)_10_-DEACM X-PP-InsP_5_ (*X* = 1,3,5) isolated from (A) MIN6 cell extract and (B) living MIN6 cells (only for (AB)_10_-DEACM 5-PP-InsP_5_). (A) Lane 1: 10 nmol (AB)_10_-DEACM 1-PP-InsP_5_, 10 min, UV irradiation (5 min). Lane 2: 10 nmol (AB)_10_-DEACM 3-PP-InsP_5_, 10 min, UV irradiation (5 min). Lane 3: 10 nmol (AB)_10_-DEACM 5-PP-InsP_5_, 10 min, UV irradiation (5 min). Lane 4: 10 nmol (AB)_10_-DEACM 1-PP-InsP_5_, 10 min, UV irradiation (1 min). Lane 5: 10 nmol (AB)_10_-DEACM 3-PP-InsP_5_, 10 min, UV irradiation (1 min). Lane 6: 10 nmol (AB)_10_-DEACM 5-PP-InsP_5_, 10 min, UV irradiation (1 min). Lane 7: 10 nmol (AB)_10_-DEACM 1-PP-InsP_5_, 10 min, no UV irradiation. Lane 8: 10 nmol (AB)_10_-DEACM 3-PP-InsP_5_, 10 min, no UV irradiation. Lane 9: 10 nmol (AB)_10_-DEACM 5-PP-InsP_5_, 10 min, no UV irradiation. Lane 10: mixture of the standards DEACM 5-PP-InsP_5_, 5-PP-InsP_5_, InsP_6_. Lane 11: DEACM 1-PP-InsP_5_ (control). (B) Lane 1: Poly-P_25_ standard. Lane 2: InsP_6_ (control), Lane 3: 5-PP-InsP_5_ (control). Lane 4: 30 μM (AB)_10_-DEACM 5-PP-InsP_5_, 24 h. Lane 5: 30 μM (AB)_10_-DEACM 5-PP-InsP_5_, 24 h, UV irradiation.

Next, the AB cleavage process was studied in MIN6 cell extract to validate the cleavage of protecting groups and thereby the release of DEACM caged X-PP-InsP_5_ 9, 14, *ent*-14 from AB prometabolites. Incubation of prometabolites in cell extract, followed by TiO_2_ enrichment and resolution of the digest on polyacrylamide gels (PAGE)^34^ and staining with toluidine blue is shown in Fig. 2A. DEACM 5-PP-InsP_5_ 9 was identified by comigration with a synthetic standard as the product of enzymatic hydrolysis, involving complete and selective removal of 10 AB groups in less than 10 min of incubation. Only minor amounts of InsP_6_ 1 due to hydrolysis of the P-anhydride were identified.^26^ UV irradiation using a 1000 W arc lamp quantitatively released 5-PP-InsP_5_ 2 from the caged compound 9 in MIN6 extract in less than five minutes. A comparable outcome was observed when repeating the experiments with the 1-PP-InsP_5_ prometabolite 13 and its enantiomer *ent*-13 (Fig. 2A), demonstrating the versatility of the approach.

Subsequently, we analyzed the ability of MIN6 cells to take up the prometabolite 8 and to release DEACM 5-PP-InsP_5_ 9. MIN6 cells incubated with 8 (30 μM) were lysed after prolonged exposure (24 h; Fig. 2B) and the lysate was enriched for highly phosphorylated metabolites using TiO_2_ extraction.^35^ The recovered molecules were then analyzed by PAGE. DEACM 5-PP-InsP_5_ 9 was recovered from MIN6 cells after 24 hours of incubation, thus demonstrating delivery and metabolic stability of 9 in live cells (Fig. 2B, lane 4). This procedure was repeated for the nonsymmetric prometabolites 13 and *ent*-13 and worked equally well (Fig. S1 in the ESI†).

Next, cellular uptake by MIN6 cells was verified using confocal microscopy. We found comparable loading of compounds after 4 h of incubation on MIN6 cells (Fig. S2 in the ESI†). Here, coumarin fluorescence (DEACM) was used to analyze subcellular localization of the probe. Z-stack analysis revealed homogenous distribution of all DEACM X-PP-InsP_5_s within internal membranes, whereas no probe was observed in nuclei (Fig. S3 in the ESI†). The proof of cellular uptake of 8 into live MIN6 cells and the quantitative and rapid release of 9 from 8 including the selective photolysis of 9 to 5-PP-InsP_5_ are important prerequisites for the application of caged compounds *in cellulo*.

**Fig. 3:**
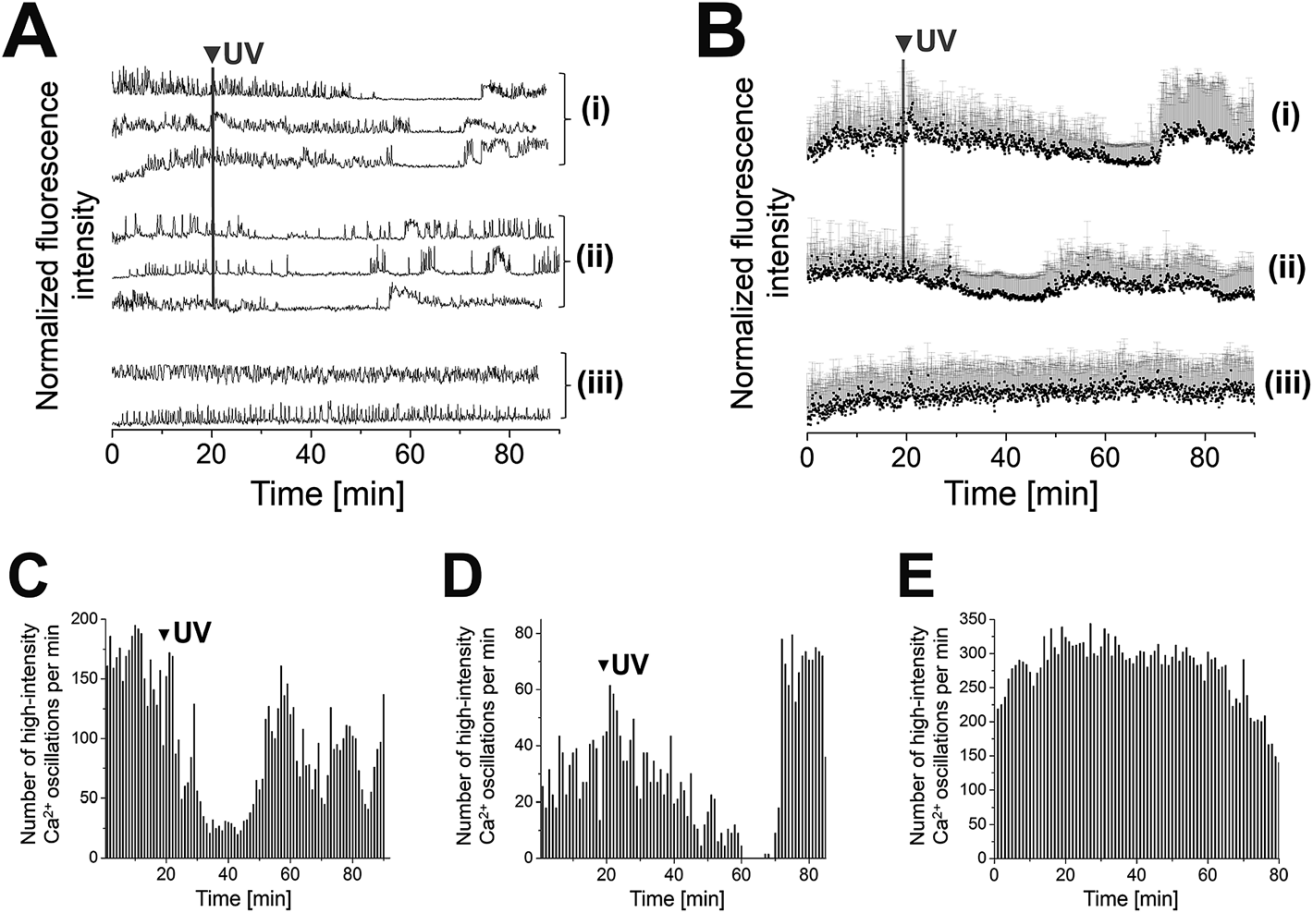
Photolysis of (AB)_10_-DEACM 5-PP-InsP_5_ in MIN6 cells. (A + B) Representative single (A) and averaged (B) Ca^2+^ traces from MIN6 cells, recorded with the Ca^2+^ indicator Fluo-4. (i) Photolysis of (AB)_10_-DEACM 5-PP-InsP_5_, cell population I, (ii) photolysis of (AB)_10_-DEACM 5-PP-InsP_5_, cell population II (ratio population I : II ∼ 1 : 4), (iii) (AB)_10_-DEACM 5-PP-InsP_5_, -UV control. (C–E) Number of detected high-intensity Ca^2+^ events within every 60 s interval. (C) (AB)_10_-DEACM 5-PP-InsP_5_, cell population I; (D) (AB)_10_-DEACM 5-PP-InsP_5_, cell population II; (E) (AB)_10_-DEACM 5-PP-InsP_5_, -UV control. For representative individual Ca^2+^ traces see ESI Fig. S4.† MIN6 cells were loaded with the compounds (10 μM) for 4 h before imaging, which was conducted in the presence of 11 mM glucose. Photolysis: *λ* = 375 nm, 10 frames, 3.2 s frame time (indicated as: UV). *n* > 40 cells in 4 experiments. Error bars present SD.

**Fig. 4:**
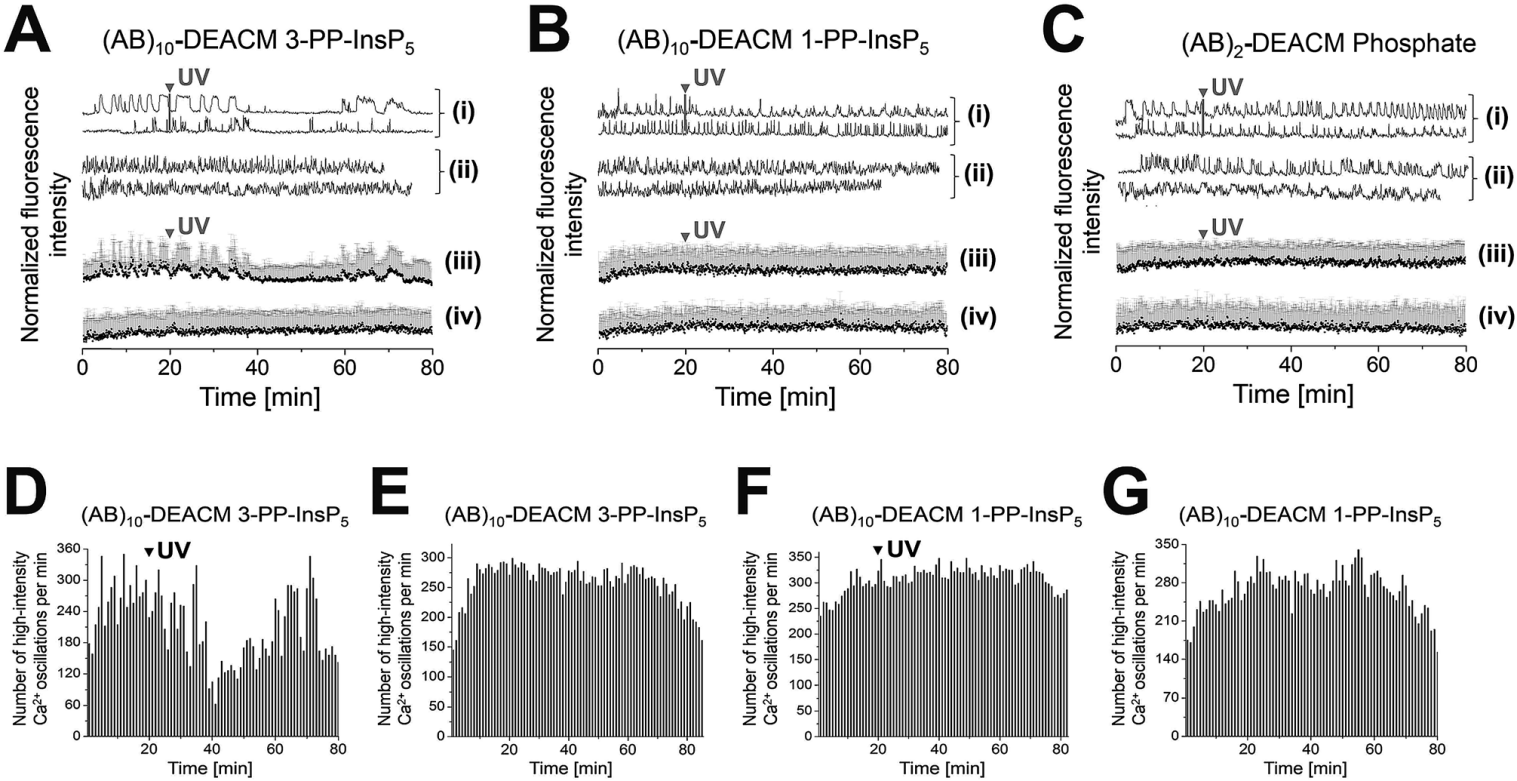
Photolysis of (AB)_10_-DEACM 1-, 3-PP-InsP_5_ and of (AB)_2_-DEACM Phosphate in MIN6 cells. (A–C) Representative single (i + ii) and averaged (iii + iv) Ca^2+^ traces from MIN6 cells, recorded with the Ca^2+^ indicator Fluo-4. (i + iii) Photolysis and (ii + iv) -UV controls of (A) (AB)_10_-DEACM 3-PP-InsP_5_, (B) (AB)_10_-DEACM 1-PP-InsP_5_, and of (C) (AB)_2_-DEACM Phosphate. (D–G) Number of detected high-intensity Ca^2+^ events within every 60 s interval. (D) (AB)_10_-DEACM 3-PP-InsP_5_; (E) (AB)_10_-DEACM 3-PP-InsP_5_, -UV control; (F) (AB)_10_-DEACM 1-PP-InsP_5_; (G) (AB)_10_-DEACM 1-PP-InsP_5_, -UV control. For representative individual Ca^2+^ traces see ESI Fig. S5–S7.† MIN6 cells were loaded with (AB)_10_-DEACM 1- and 3-PP-InsP_5_ (10 μM) for 4 h before imaging, which was conducted in the presence of 11 mM glucose. Photolysis: *λ* = 375 nm, 10 frames, 3.2 s frame time (indicated as: UV). *n* > 40 cells in 4 experiments. Error bars present SD.

These results served as a starting point for monitoring cellular effects on β-cells of different PP-InsPs after uncaging. [Ca^2+^]_i_ oscillations in MIN6 cells provided a highly sensitive and physiologically relevant read-out for monitoring the effects of the rapid increase of the metabolic messengers upon UV-irradiation. The oscillations were monitored at the single β-cell level using the Ca^2+^-sensitive indicator Fluo-4.

(AB)_10_-DEACM 5-PP-InsP_5_ loaded MIN6 cells were grouped into two populations that behaved differently, following photolysis. Consistently, both populations transiently reduced their [Ca^2+^]_i_ oscillations, for *ca.* 20 min (cell population I) or *ca.* 10 min (population II) after UV-irradiation (*λ* = 375 nm, Fig. 3A, B(i, ii) and C, D). Population II was of significantly higher abundance compared to population I (ratio 4 : 1). Even though MIN6 cells within population I stopped [Ca^2+^]_i_ oscillations at different time points, recovery of cell activity occurred almost simultaneously within cells at different sites in the field of view. Comparable effects were observed for MIN6 cells of population II (Fig. 3A and B).

(AB)_10_-DEACM 3-PP-InsP_5_ loaded MIN6 cells transiently reduced their [Ca^2+^]_i_ oscillations, *ca.* 15 min after UV-irradiation (*λ* = 375 nm, Fig. 4A(i, iii) and D), with no effects in the -UV control (Fig. 4A(ii, iv) and E). No comparable effects were observed upon irradiation of (AB)_10_-DEACM 1-PP-InsP_5_ pre-loaded MIN6 cells (*λ* = 375 nm, Fig. 4B(i, iii) and F). 3-PP-InsP_5_ is the non-natural enantiomer of 1-PP-InsP_5_ and is not degraded by the enzymes responsible for inositol pyrophosphate turnover, whereas 1-PP-InsP_5_ is rapidly metabolized.^36^ This metabolic instability might explain the differential behavior of the two enantiomers in the presented assay. We applied (AB)_2_-DEACM phosphate as a negative control in MIN6 cell experiments and no effects on [Ca^2+^]_i_ oscillations were observed upon UV irradiation of loaded MIN6 cells (*λ* = 375 nm, Fig. 4A, B(v) and ESI Fig. S6†). Also, vehicle-loaded MIN6 cells did not show changes in [Ca^2+^]_i_ oscillations upon UV irradiation (*λ* = 375 nm, ESI, Fig. S7†).

## Conclusion

In summary, this study provides advanced synthetic procedures for the preparation of complex inositol pyrophosphate prometabolites and of PP-InsPs that are additionally equipped with DEACM photocages. Such photocaged prometabolites of inositol pyrophosphates have not been reported before. The modifications are not only introduced on symmetric 5-PP-InsP_5_ but also on the two enantiomers 1- and 3-PP-InsP_5_ by applying asymmetric phosphorylation. The photocaged prometabolites enter live cells and can efficiently release the caged precursor molecule involving clean enzymatic removal of ten protecting groups. The procedure is simple, as it only requires incubation of cells with the prometabolite to achieve efficient loading. Upon photolysis, the effects of a controlled intracellular increase of different inositol pyrophosphate species were read out at the single cell level and averaged on ensembles of cells. The controlled increase of the intracellular 5-PP-InsP_5_ concentration was found to translate into a transient decrease of [Ca^2+^]_i_ oscillations within MIN6 cells. In contrast, 1-PP-InsP_5_, the other relevant cellular inositol pyrophosphate with seven phosphate groups, did not show any of these effects but, interestingly, its unnatural enantiomer did. The underlying mechanisms still have to be identified. This study shows for the first time the modulation of β-cell activity upon temporally defined photo-release of inositol pyrophosphate species. In addition to the described effects on cellular [Ca^2+^]_i_ oscillations, we propose that the presented tools and strategies will be of high value to also study other effects that are associated with the PP-InsPs.^14^,^37^

## Conflicts of interest

There are no conflicts to declare.

## Supplementary Material

Supplementary information
