# Supplementary material for: Photolysis of cell-permeant caged inositol pyrophosphates controls oscillations of cytosolic calcium in a β-cell line
Source: Chem Sci. 2019 Jan 10;10(9):2687–92. doi: 10.1039/c8sc03479f (PMC6419925; doi:10.1039/c8sc03479f)
Supplement: Supplementary file 1 [file SC-010-C8SC03479F-s001.pdf]

Electronic Supplementary Material (ESI) for Chemical Science.
This journal is © The Royal Society of Chemistry 2019

Supplemental lnformation (SI)

Photolysis of cell-permeant caged inositol pyrophosphates controls oscillations of cytosolic calcium in a *β*-cell line

S. Hauke[1,%], A. K. Dutta[2,%], V. Eisenbeis[2], D. Bezold[2], T. Bittner[2], C. Wittwer[2], D. Thakor[2,3], I. Pavlovic[2,3], C. Schultz[1,4]*, H. J. Jessen[2,]*

[1]European Molecular Biology Laboratory (EMBL), Cell Biology & Biophysics Unit, Meyerhofstrasse 1, 69117 Heidelberg, Germany

[2] Albert-Ludwigs University Freiburg, Department of Chemistry and Pharmacy, Albertstrasse 21, 79104 Freiburg i.B., Germany

[3] University of Zurich, Department of Chemistry, Winterthurerstrasse 190, 8057 Zurich, Switzerland

[4] Oregon Health & Science University (OHSU), Department of Physiology and Pharmacology, Mail code: L334 3181. S.W. Sam Jackson Park Road Portland, OR 97239-3098, USA.

% equal contribution

Abbreviations

APS	Ammonium persulfate
BSTFA	*N*,*O*-Bis(trimethylsilyl)trifluoroacetamide
CH_2Cl_2	Dichloromethane
DBU	1,8-Diazabicyclo[5.4.0]undec-7-ene
DMEM	Dulbecco's modified eagle's medium
DMF	Dimethylformamide
DMSO	Dimethyl sulfoxide
DTT	Dithiothreitol
EDTA	Ethylenediaminetetraacetic acid
Et_2O	Diethyl ether
ETT	5-(Ethylthio)-1*H*-tetrazole
FBS	Fetal bovine serum
HEPES	*4*-(2-hydroxyethyl)-1-piperazineethanesulfonic acid
LT-ELSD	Low temperature evaporative light scattering detector
MeOH	Methanol
*m*CPBA	*meta*-Chloroperoxybenzoic acid
MPLC	Medium pressure liquid chromatography
PA	Perchloric acid
PBS	Phosphate-buffered saline
Pip	Piperidine
RP-HPLC	Reverse phase high-performance liquid chromatography
TBA	Tetrabutylammonium
TEAA	Triethylammonium acetate
TEMED	Tetramethylethylenediamine
TFA	Trifluoroacetic acid
TiO_2	Titanium dioxide
TLC	Thin layer chromatography

Methods & Analyses

A) Biological methods & analyses

General. Chemicals and solvents were obtained from Sigma-Aldrich (St. Louis, MO & Germany), Alfa Aesar (Karlsruhe, Germany), Grüssing (Filsum, Germany), PAN biotech (Aidenbach, Germany) and Roth (Karlsruhe, Germany) unless stated otherwise; gradient-grade FBS and DMEM were obtained from Gibco (Carlsbad, Ca, U.S. & Germany).

Tissue culture. Insulin-secreting MIN6 *β*-cells[1] were cultured at 37 °C in an 8 % high humidity CO_2 atmosphere. MIN6 cells were grown in DMEM containing 4.5 g/L glucose, supplemented with 15 % FBS and 70 µM *β*-mercaptoethanol. MIN6 cells were plated onto 8-well LabTek microscope dishes (155411 Thermo Scientific) to form pseudoislets.

Polyacrylamide gel electrophoresis (PAGE). PAGE was carried out on a Hoefer SE660 Tall Standard Dual Cooled Vertical Unit. The PAGE procedure was conducted according to the general procedure as described by Losito *et al*.[2] Buffers and solutions for gel electrophoresis were prepared as follows:

10 × Tris/Borate/EDTA (TBE) buffer, pH 8.3	0.89 M Tris-HCl, 0.89 M boric acid, 20 mM EDTA
1 × Orange G dye, pH 7.0	10 mM Tris-HCl, 1 mM EDTA, 30% (w/v) glycerol, 0.1% (w/v) Orange G
Staining solution	0.1% (w/v) toluidine blue, 20% (w/v) MeOH, 2% (w/v) glycerol
De-staining solution	20% (w/v) MeOH, 2% (w/v) glycerol

During pre-run and run, the lower buffer chamber was filled with 6 L of pre-chilled 1 × TBE buffer (4 °C) and the buffer was stirred. A recirculating cooler was used for chilling the buffer. Sample loading was performed with gel-loading pipet tips. Poly-P_{25}, $InsP_6$, 5-PP-$InsP_5$, 1-PP-$InsP_5$, DEACM 5-PP-$InsP_5$ and DEACM 1-PP-$InsP_5$ were used as references.

<u>Electrophoretic separation of 5-PP-$InsP_5$ and its analogues</u>:

1. The gel sandwich was assembled using glass plates (24 × 18 cm) and spacers (1 cm wide, 1.0 mm thick).
2. Gel preparation (~ 40 mL/gel): 35.8 % (w/v) acrylamide:bis-acrylamide 19:1 (33.9 mL, 3030 Roth), 10.0 % (v/v) 10 × TBE buffer (3.8 mL) and 0.05 % (w/v) ammonium persulfate (APS) (200 µL of 10 % APS in milli-Q H_2O) were stirred for 2 min at 0 °C. 0.05 % (v/v) TEMED (20 µL) was added and the solution was stirred for 1 min. The mixture was poured between the pre-casted glass-plates and a 15 lane comb was inserted. The solution was allowed to polymerize for 25-30 min at room temperature.
3. After polymerization, gels were pre-run at 4 °C in 1 × TBE buffer for 30 min at 300 V.
4. Samples and references (0.09 mM Poly-P_{25}, 0.11 mM $InsP_6$, 0.06 mM 5-PP-$InsP_5$, 0.06 mM 1-PP-$InsP_5$, 0.07 mM DEACM 5-PP-$InsP_5$, 0.07 mM DEACM 1-PP-$InsP_5$; milli-Q H_2O as solvent, 22 µL volume per control sample) were prepared. 1 × Orange G dye (5-7 µL) was added to all samples and references (= control samples) prior to loading onto the gel.
5. Wells were washed with 1 × TBE buffer by using a syringe and needle to remove any precipitates and non-polymerized gel debris. The gel was then loaded leaving 2-3 wells empty on each side.
6. Gels were run at 4 °C in 1 × TBE buffer for 20 h at 500 V.
7. After the run, the gel apparatus was disassembled. One glass plate was removed leaving the gel on the other glass plate.
8. Workup: Gels were stained for 30 min with staining solution and then de-stained for 2 h. The de-staining solution was replaced 2-3 times during the entire procedure.
9. Finally, the gels were scanned with a photo scanner.

Cellular uptake and AB cleavage.

For cell viability assays, a microplate reader (TECAN SPARK 10M) was used and measurements were performed in PBS. Viability was checked by adding trypan blue (0.4 % in PBS) to the cell suspension. For *in vitro* and *in cellulo* studies, 5 mM stock solutions of $(AB)_{10}$-DEACM 1-PP-$InsP_5$ ($C_{122}H_{144}N_3O_{49}P_7$, Mol. mass 2653.28), $(AB)_{10}$-DEACM 3-PP-$InsP_5$ ($C_{122}H_{144}N_3O_{49}P_7$, Mol. mass 2653.28) and $(AB)_{10}$-DEACM 5-PP-$InsP_5$ ($C_{124}H_{141}N_4O_{49}P_7$, Mol. mass 2688.29) in DMSO were prepared and stored at −20 °C. UV irradiation of biological assays was performed with an UV lamp (Herolab, UV-6 S/L) or an arc lamp (Newport, OPS-A1000). For perchloric acid (PA) extraction of mammalian cells, TiO_2 beads (Titansphere, 5 µm, 5020-75000 GL Sciences) were used as described by Wilson *et al*.[3,4] The extraction steps until elution were performed at 4 °C and perchloric acid (PA) solution was applied in 1 M concentration.

Cellular uptake into MIN6 cells:

1. For *in cellulo* experiments, MIN6 cells (2-3 × 10^6 cells) were seeded and media was added. Cells were incubated for 24 h at 37 °C in a 5 % CO_2 atmosphere.
2. $(AB)_{10}$-DEACM X-PP-$InsP_5$ (X = 1,3,5) was added (final concentration: 30 µM) and distributed equally. Cells were incubated for 24 h as indicated in step 1.
3. If uncaging was desired, the lid of the culture dish was removed and the sample was irradiated with a UV lamp (30 min at room temperature, λ = 365 nm, minimum distance between lamp and sample) or an arc lamp (5 min on ice, λ = 400 nm, 1000 W, 20 cm distance between lamp and sample).
4. Cells were detached using Trypsin-EDTA. Trypsin activity was quenched and cells were centrifuged (5 min, 200 g).
5. The supernatant was discarded and cells were washed with PBS. The suspension was centrifuged (5 min, 200 g) and the liquid was discarded. The washing step was repeated. The pellet was stored at −20 °C or processed immediately.

6. TiO_2 beads (4-5 mg per sample) were washed with milli-Q H_2O (500 µL) and centrifuged (1 min, 3500 g, 4 °C). The washing step was repeated with PA (500 µL). Beads were then re-suspended in PA (500 µL).
7. The cell pellet was suspended in PA (500 µL) and incubated on ice for 10 min under frequent vortexing.
8. The suspension was centrifuged (5 min, 17000 g, 4 °C), the pellet was discarded and the supernatant was added to the TiO_2 beads. The sample was rotated for 20 min at 4 °C.
9. The beads were pelleted by centrifugation (1 min, 3500 g, 4 °C) and washed twice with PA (500 µL).
10. Phosphorylated compounds were eluted by the addition of 10 % NH_4OH (200 µL), followed by rotation for 5 min. The sample was centrifuged and the supernatant was collected.
11. The elution step was repeated and the supernatants were combined.
12. Samples were evaporated under reduced pressure and at 30 °C until a final volume (< 30 µL) was reached. 1 × Orange G dye (5-7 µL) was added to each samples and the samples were loaded on a polyacrylamide gel as described before.
13. Beads were washed for three times with milli-Q H_2O (500 µL) before storage at 4 °C; the beads are reusable for the same cell line.

AB cleavage in MIN6 cell extracts:

1. For *in vitro* experiments, MIN6 cells were grown to 80-90% confluence.
2. Cells were collected by adding Trypsin-EDTA. The cell suspension was centrifuged (5 min, 200 g) and the supernatant was discarded.
3. Cells were placed on ice and washed with PBS. The suspension was centrifuged (5 min, 200 g, 4 °C) and the supernatant was discarded. The washing step was repeated.
4. Cells were counted and cell viability was determined.
5. Lysis buffer (EPX-99999-000 ThermoFisher, 1 mL/5× 10^7 cells) and protease inhibitor cocktail (P8340 Sigma-Aldrich, 10 µL/mL lysis buffer) were added to the pellet.

6. Homogenization was performed (20 times) by using a tissue homogenizer (Wheaton Dounce tissue grinder, 1 mL, tight pestle). To ensure complete cell lysis, three freeze and thaw cycles were successively implemented.

7. The homogenate was centrifuged (20 min, 17000 g, 4 °C).

8. The supernatant was transferred into a new centrifuge tube and step 7 was repeated.

9. Supernatants were stored on ice and the protein concentration was measured (41.9 mg/mL) with a nanophotometer (Implen Nanophotometer N60)

10. Reaction mixtures were prepared as follows. The reaction buffer (200 mM HEPES, 60 mM $MgSO_4$, 1 M NaCl, 10 mM DTT, pH 8.8) was freshly prepared and the final concentration of $(AB)_{10}$-DEACM X-PP-$InsP_5$ (X = 1,3,5) in every reaction mixture was 0.33 mM.

**Reaction mixture**	**Amounts [µL]**
Reaction buffer	3
Cell extract	20
milli-Q H_2O	5
5 mM $(AB)_{10}$-DEACM X-PP-$InsP_5$	2

14. All reaction mixtures were incubated (37 °C, 900 rpm) for 10 min.

11. If uncaging was desired, the mixtures were irradiated with an arc lamp (on ice, λ = 400 nm, 1000 W, 20 cm distance between lamp and sample) for the respective time intervals

12. The reaction mixtures were quenched by the addition of EDTA (1 µL, 0.5 M, pH 8.0), followed by incubation on ice for 5 min.

13. Inositol pyrophosphates were extracted from the reaction mixtures using TiO_2 beads.

14. TiO_2 beads (4-5 mg per sample) were washed with milli-Q H_2O (500 µL) and centrifuged (1 min, 3500 g). The washing step was repeated with PA (500 µL). Beads were then re-suspended in PA (500 µL).

15. The bead suspensions were added to the reaction mixtures and vortexed briefly.

16. Samples were rotated for 20 min and at 4 °C.

17. Beads were pelleted (1 min, 3500 g, 4 °C) and supernatants were discarded.
18. Beads were washed with PA (500 µL) and centrifuged (1 min, 3500 g, 4 °C). Supernatants were discarded and washing was repeated.
19. Phosphorylated compounds were eluted by the addition of 10 % NH_4OH (200 µL), followed by brief vortexing and rotation for 5 min. Samples were centrifuged and supernatants were collected.
20. The elution step was repeated and the supernatants were combined.
21. The samples were evaporated under reduced pressure and at 30 °C until a final volume (< 30 µL) was reached. 1 × Orange G dye (5 µL) was added to the samples and they were loaded on a polyacrylamide gel as described before.

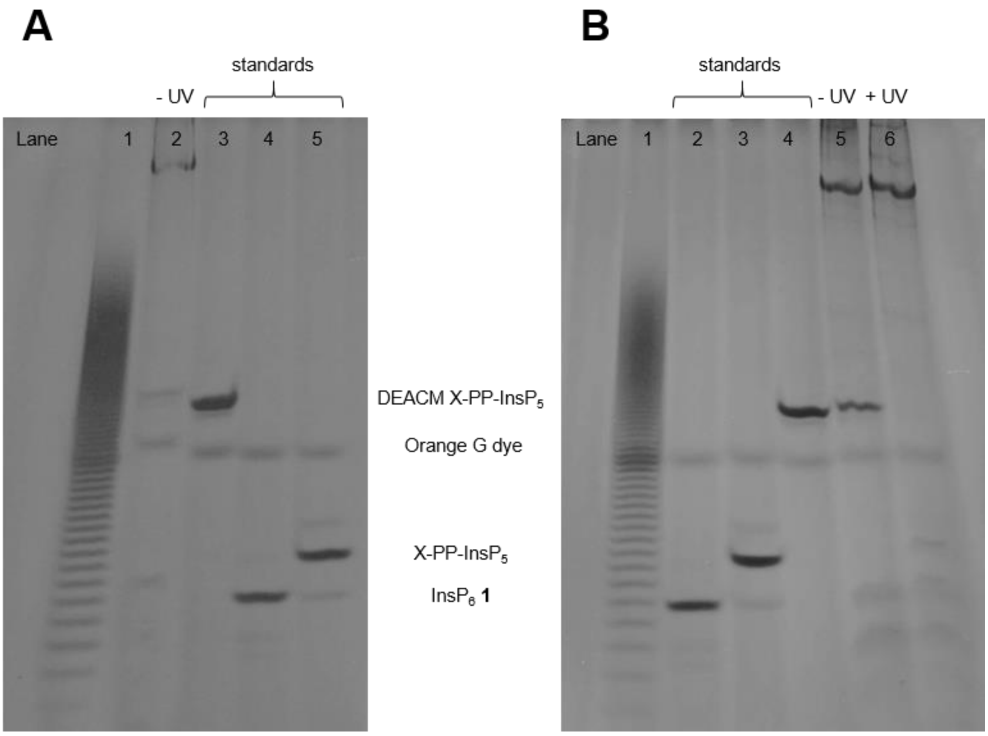

Figure S1: PAGE analysis of (A) $(AB)_{10}$-DEACM 1-PP-$InsP_5$ and (B) $(AB)_{10}$-DEACM 3-PP-$InsP_5$ isolated from living MIN6 cells. (**A**) Lane 1: Poly-P_{25} standard. Lane 2: 30 µM $(AB)_{10}$ DEACM 1-PP-$InsP_5$, 24 h. Lane 3: DEACM 1-PP-$InsP_5$ (control). Lane 4: $InsP_6$ (control). Lane 5: 1-PP-$InsP_5$ (control). (**B**) Lane 1: Poly-P_{25} standard. Lane 2: $InsP_6$ (control). Lane 3: 1-PP-$InsP_5$ (control). Lane 4: DEACM 1-PP-$InsP_5$ (control). Lane 5: 30 µM $(AB)_{10}$-DEACM 3-PP-$InsP_5$, 24 h. Lane 6: 30 µM $(AB)_{10}$-DEACM 3-PP-$InsP_5$, 24 h, UV irradiation.

Confocal laser scanning microscopy. Imaging was performed on a FluoView1200 (Olympus IX83) confocal laser scanning microscope at 37 °C (incubator box made by EMBL), using Olympus 60x Plan-APON (NA 1.4, oil) or 20x UPLS APO (NA 0.75, air) objectives and FluoView software, version 4.2. The images were acquired with a Hamamatsu C9100-50 EM CCD camera. The green channel was imaged using a 488 nm laser line (120 mW/cm^2, 2.5%) and a 525/50 emission mirror. The red channel was imaged using a 559 nm laser (120 mW/cm^2, 2.0%) and a 643/50 emission filter. Images were acquired in 4 s intervals (frame time: 3.9 s). A pulsed 375 nm laser (10 MHz) was applied for uncaging experiments in the entire field of view for 8 frames (3.2 s/frame). The dual scanner set up allowed for simultaneous laser stimulation and confocal imaging. This permitted capturing of cellular responses that occur during or immediately after laser stimulation. To test changes in $[Ca^{2+}]_i$ at the single cell level, MIN6 cells, grown to 70 % confluence in pseudo-islets were incubated with an acetoxymethyl ester of the Ca^{2+}-indicator Fluo-4 (Life Technologies, Eugene, OR), 5 µM in DMEM (1 g/L glucose) for 20 min at 37 °C. Imaging was performed in standard HEPES buffer (in mM: 115 NaCl, 1.2 $CaCl_2$, 1.2 $MgCl_2$, 1.2 K_2HPO_4 and 20 HEPES, pH 7.4). All imaging experiments were conducted at 11 mM glucose, if not stated otherwise.

Data analysis. Fluorescence intensities were extracted from individual cells as a function of time using Fiji[5] and expressed relative to the maximum detected fluorescence intensity after subtraction of background (F/F_0). Representative cells within the field of view were averaged to generate Ca^{2+} traces or to determine the number of detected high-intensity Ca^{2+} events within every 60 s interval. The height of each $[Ca^{2+}]_i$ event was determined relative to the highest detected peak in each trace as a criterion to group Ca^{2+} transients into high-intensity (≥ 60% of highest peak) and low-intensity (< 60% of highest peak) events.

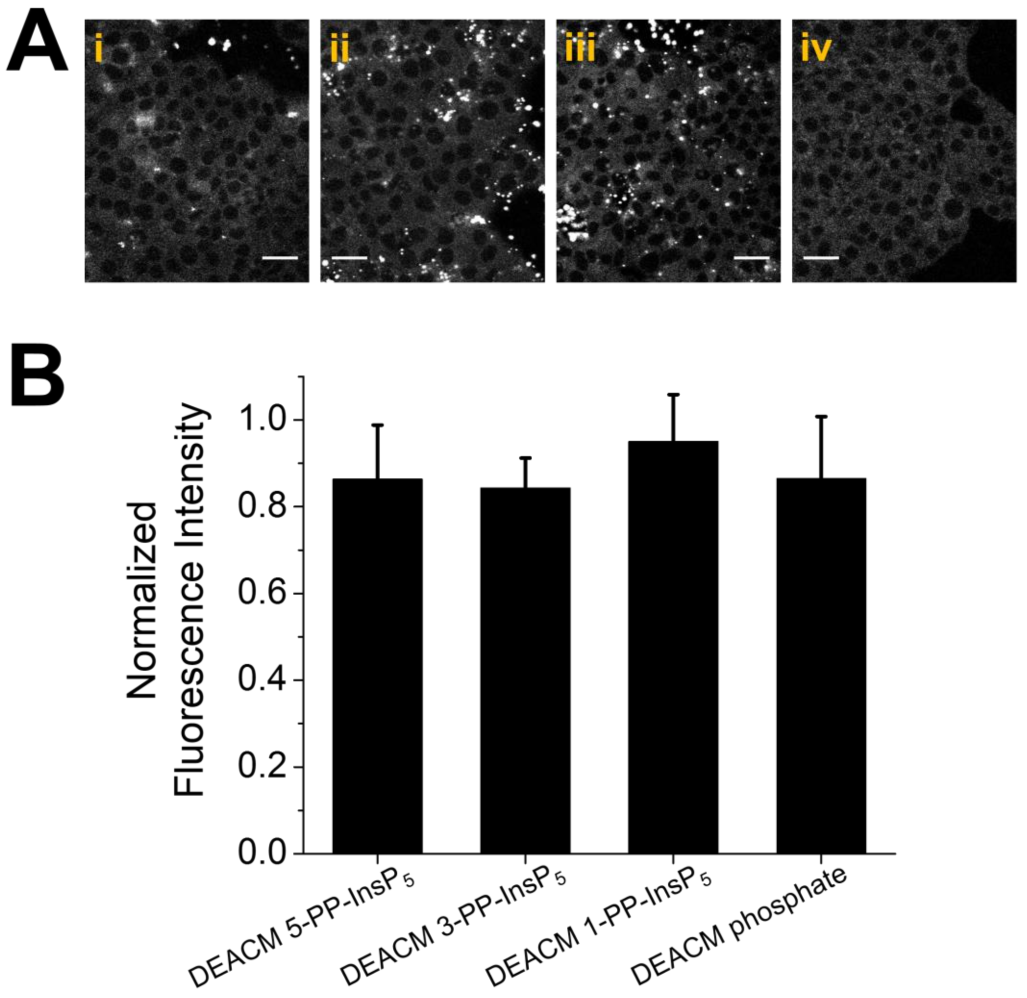

Figure S2. Monitoring the cellular uptake of $(AB)_{10}$-DEACM X-PP-InsP$_5$ into live MIN6 cells. Representative confocal laser scanning microscopy images **(A)** and microscopy-based quantification of compound loading in 200 MIN6 cells **(B)**. **(i)** $(AB)_{10}$-DEACM 5-PP-InsP$_5$, **(ii)** $(AB)_{10}$-DEACM 3-PP-InsP$_5$, **(iii)** $(AB)_{10}$-DEACM 1-PP-InsP$_5$ and **(iv)** $(AB)_2$-DEACM-phosphate were applied on MIN6 cells for 4 h in 10 µM final concentration. The normalized fluorescence intensity was then obtained for the DEACM caged compounds without AB groups. Scale bars, 20 µm.

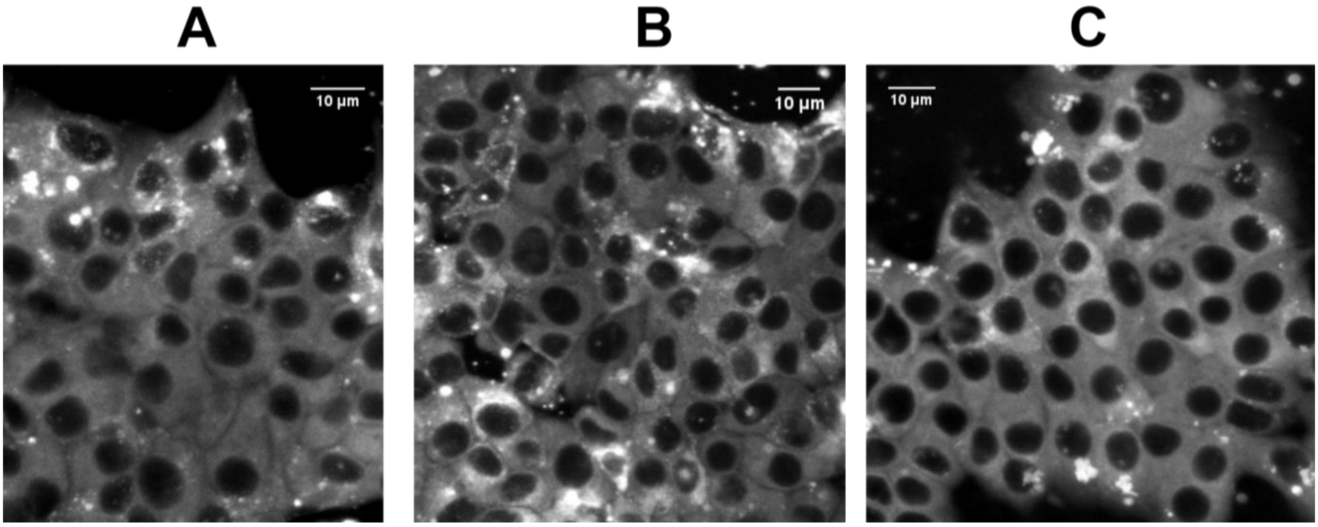

Figure S3. Z-stack analysis of the sub-cellular localization of DEACM X-PP-InsP$_5$ in live MIN6 cells. Presented (AB)$_{10}$-DEACM X-PP-InsP$_5$ were incubated (10 µM final concentration) on live MIN6 cells for 4 h in advance of imaging to allow for the complete removal of AB-groups. Z-stack analysis revealed homogenous distribution of DEACM 1-PP-InsP$_5$ **(A)**, DEACM 3-PP-InsP$_5$ **(B)** and DEACM 5-PP-InsP$_5$ **(C)** within internal membranes of live MIN6 cells. No probe was observed in nuclei. Stacks were acquired using an Olympus Plan-APON 60× (NA1.4, oil) objective, 20 slices, average intensity Z-projection. Scale bars, 10 µm.

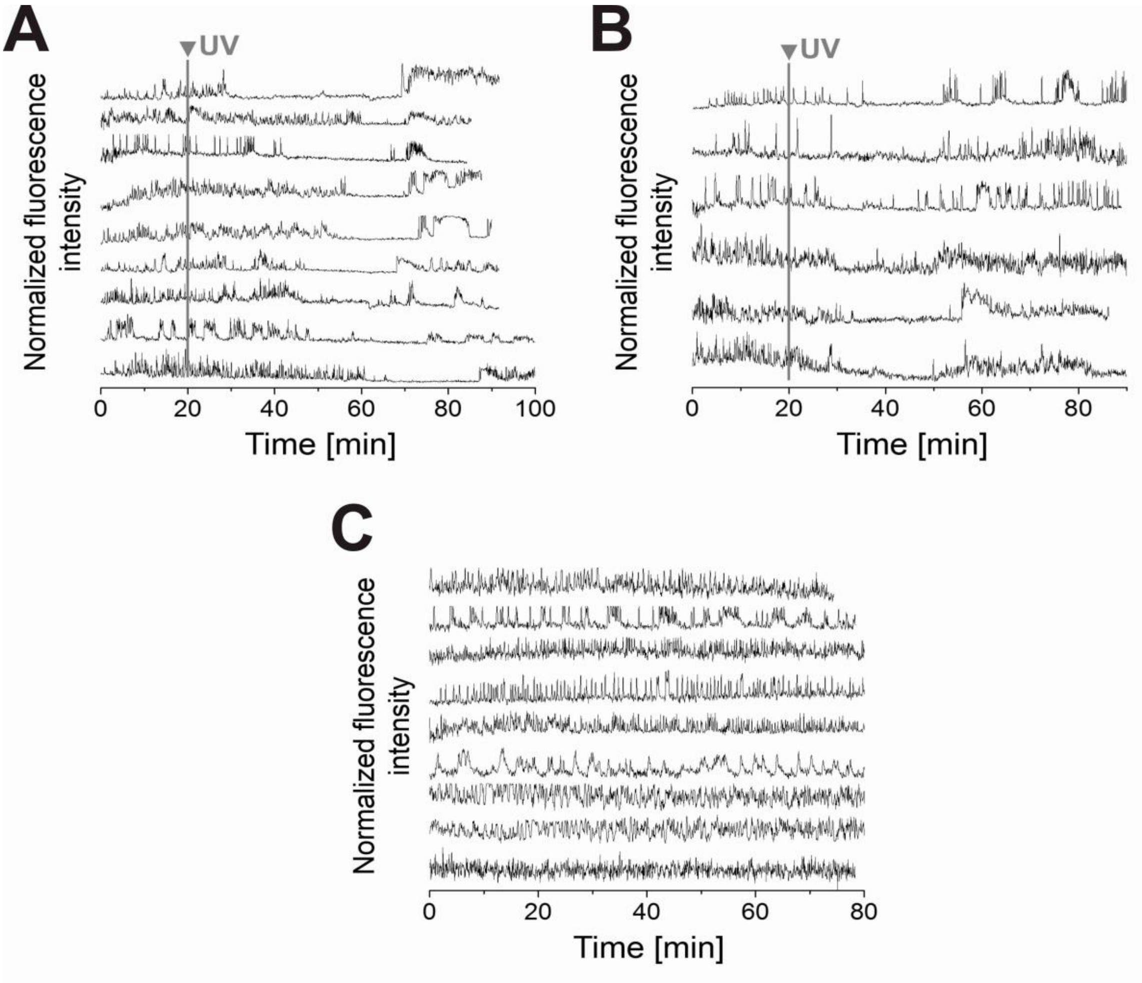

Figure S4. Photolysis of $(AB)_{10}$-DEACM 5-PP-InsP_5 in MIN6 cells. (A-D) Representative, exemplary single Ca^{2+} traces from MIN6 cells, stained with the Ca^{2+} indicator Fluo-4. **(A)** Photolysis of $(AB)_{10}$-DEACM 5-PP-InsP_5, cell population I; **(B)** photolysis of $(AB)_{10}$-DEACM 5-PP-InsP_5, cell population II. $[Ca^{2+}]_i$ oscillations of MIN6 cells transiently stopped upon photolysis of $(AB)_{10}$-DEACM 3-PP-InsP_5 to spontaneously recover. **(C)** $(AB)_{10}$-DEACM 5-PP-InsP_5, -UV control. MIN6 cells were loaded with respective compounds (10 µM) for 4 h before imaging to allow for the enzymatic removal of AB groups. Imaging was conducted at 11 mM glucose. Photolysis: λ = 375 nm, 10 frames, and 3.2 s frame time (indicated as: UV).

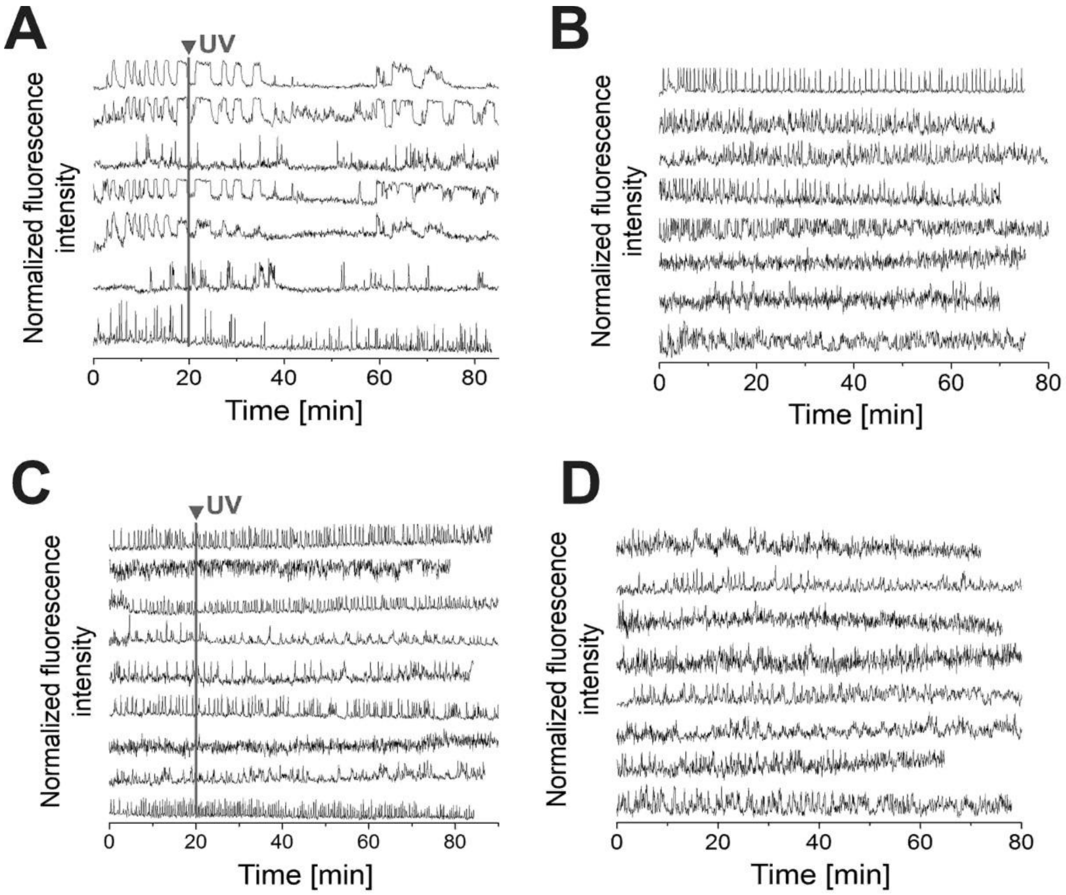

Figure S5. Photolysis of $(AB)_{10}$-DEACM 3-PP-InsP_5 and $(AB)_{10}$-DEACM 1-PP-InsP_5 in MIN6 cells. (A-D) Representative, exemplary single Ca^{2+} traces from MIN6 cells, stained with the Ca^{2+} indicator Fluo-4. **(A)** Photolysis of $(AB)_{10}$-DEACM 3-PP-InsP_5. $[Ca^{2+}]_i$ oscillations of MIN6 cells transiently stopped upon photolysis of $(AB)_{10}$-DEACM 3-PP-InsP_5 to spontaneously recover. **(B)** $(AB)_{10}$-DEACM 3-PP-InsP_5, -UV control; **(C)** photolysis of $(AB)_{10}$-DEACM 1-PP-InsP_5; **(D)** $(AB)_{10}$-DEACM 1-PP-InsP_5, -UV control. MIN6 cells were loaded with respective compounds (10 µM) for 4 h before imaging to allow for the enzymatic removal of AB groups. Imaging was conducted at 11 mM glucose. Photolysis: λ = 375 nm, 10 frames, 3.2 s frame time (indicated as: UV).

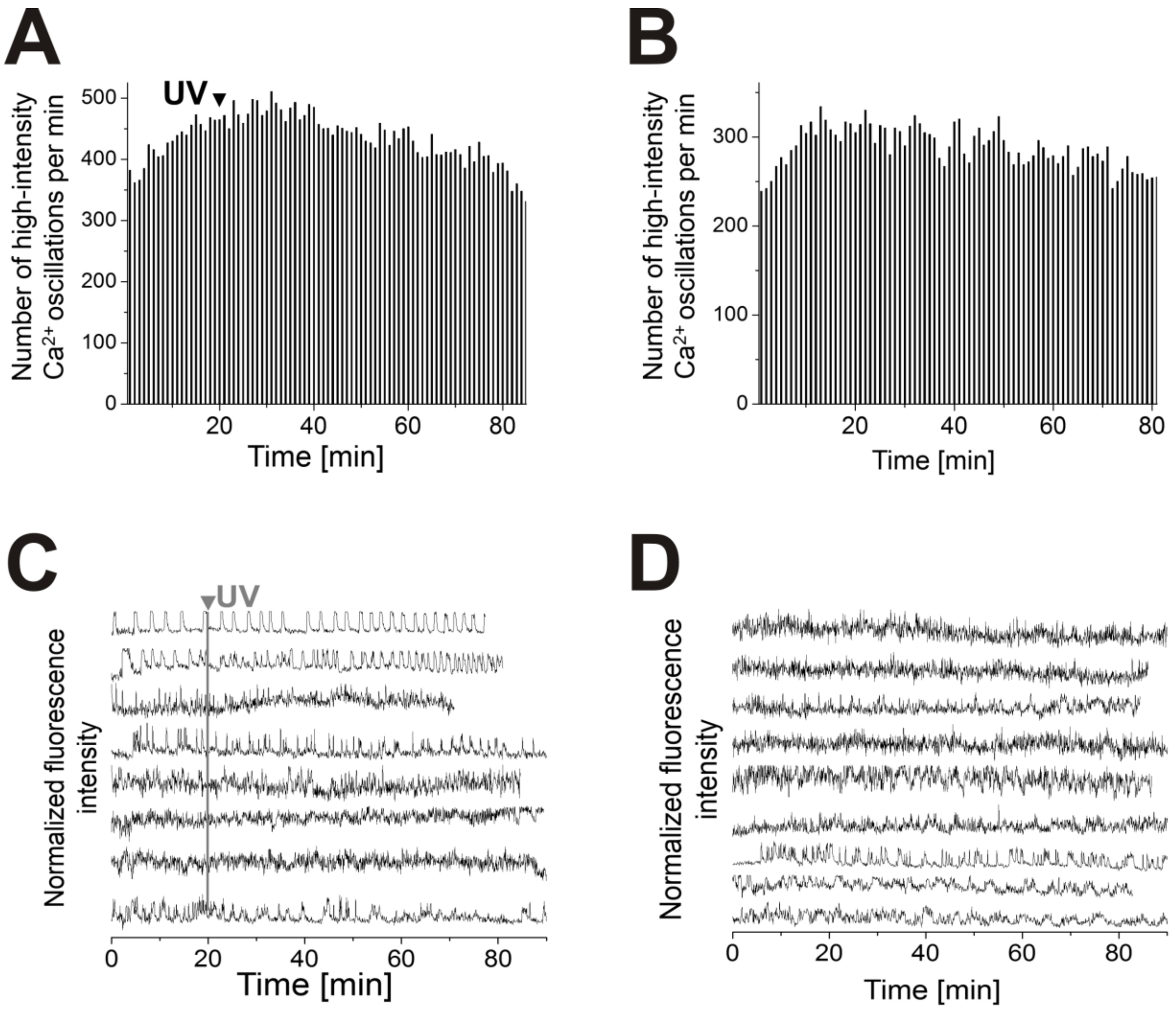

Figure S6. Photolysis of DEACM-(AB)$_2$-phosphate in live MIN6 cells. (A+B) Number of detected high-intensity Ca^{2+} events within every 60 s interval. **(C+D)** Representative, exemplary single Ca^{2+} traces from MIN6 cells, stained with the Ca^{2+} indicator Fluo-4. $[Ca^{2+}]_i$ oscillations of DEACM-(AB)$_2$-phosphate-loaded MIN6 cells remained unchanged after photolysis of DEACM-(AB)$_2$-phosphate **(A+C)** and in the -UV control **(B+D)**. MIN6 cells were loaded with respective compounds (10 µM) for 4 h before imaging to allow for the enzymatic removal of AB groups. Imaging was conducted at 11 mM glucose. Photolysis: λ = 375 nm, 10 frames, 3.2 s frame time (indicated as: UV).

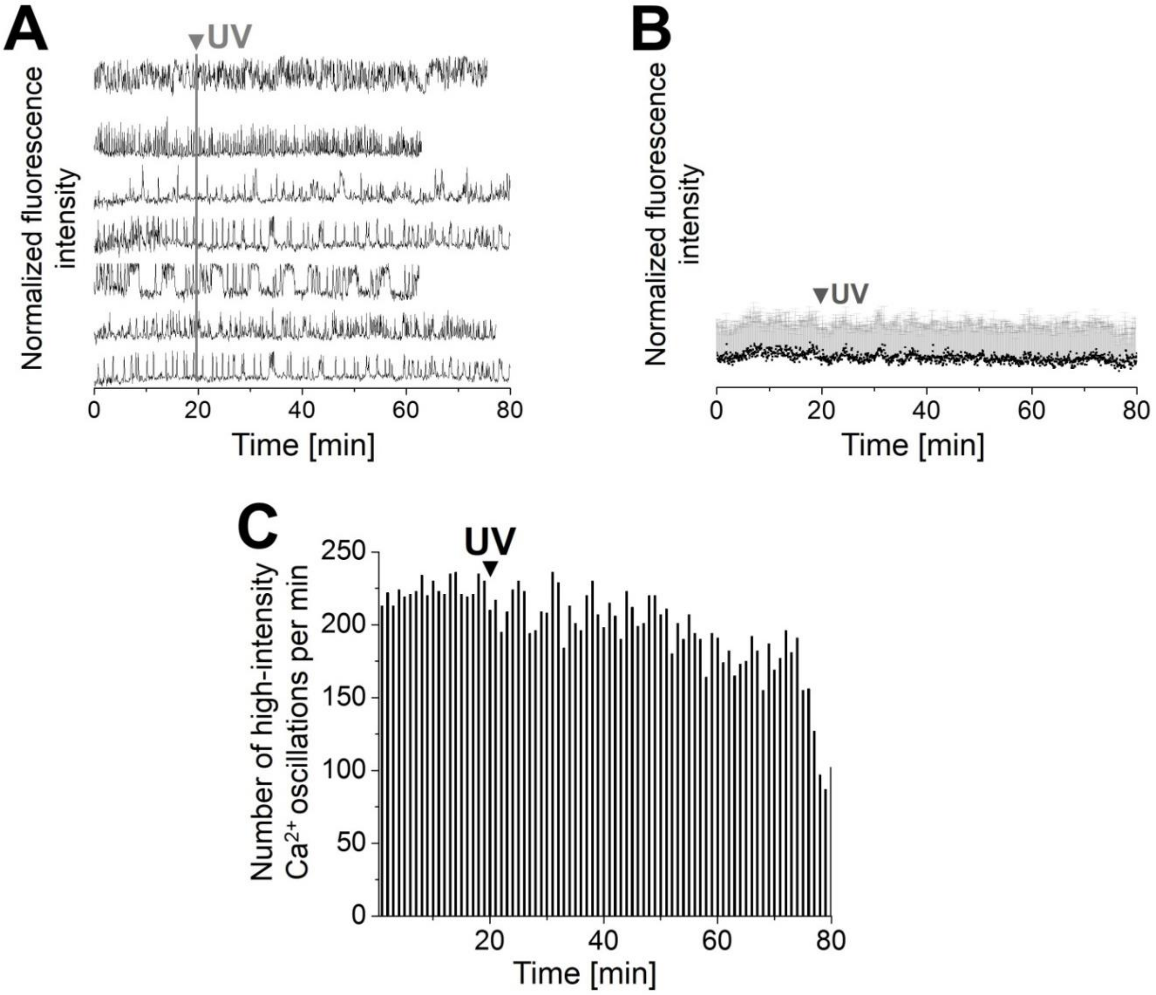

Figure S7. (A-C) UV illumination does not change the activity of vehicle-treated MIN6 cells. (A + B) Representative single **(A)** and averaged **(B)** Ca^{2+} traces from MIN6 cells, recorded with the Ca^{2+} indicator Fluo-4. **(C)** Number of detected high-intensity Ca^{2+} events within every 60 s interval. MIN6 cells were loaded with DMSO for 4 h before imaging. Imaging was conducted at 11 mM glucose. Photolysis: λ = 375 nm, 10 frames, 3.2 s frame time (indicated as: UV).

B) Synthetic methods & analyses

General remarks

Reactions were carried out using oven-dried glassware under an atmosphere of dry N_2 and magnetically stirred, unless noted otherwise. Air- and moisture-sensitive liquids and solutions were transferred via syringe or stainless steel cannula.

Reagents were purchased from commercial suppliers (Acros, Aldrich, Fluka, TCI, ChemGenes Corp.) and used without further purification, unless noted otherwise.

Solvents were obtained in analytical grade and used as received for coupling reactions, extractions, chromatography and precipitation.

Dry solvents for reactions were purified by filtration and dried by passage over activated anhydrous neutral A-2 alumina (MBraun solvent purification system) under an atmosphere of dry N_2.

Deuterated solvents for NMR and reactions were obtained from Armar Chemicals, Switzerland and euriso-top, Germany, in the indicated purity grade and used as received for NMR spectroscopy.

Thin layer chromatography was carried out using Merck silica gel 60 F254 plates, visualized with UV light or developed either with phosphormolybdic acid solution or with potassium permanganate stain followed by heating.

Flash chromatography was performed using Fluka silica gel 60 (230-400 Mesh) at a pressure of ca. 0.3 bar.
Lyophilizations were done with Christ Freeze Dryer Alpha 1-4 LDplus and Christ Freeze Dryer Alpha 1-2 LDplus.

^{1}H-NMR spectra were recorded on Bruker 300 MHz spectrometers, Bruker 400 MHz and Bruker 500 MHz spectrometers in the indicated deuterated solvent. Data are reported as follows: chemical shift (δ, ppm), multiplicity (s, singlet; d, doublet; t, triplet; q, quartet; m, multiplet; br, broad signal), coupling constant(s) (*J*, Hz), integration. All signals were

referenced to the internal solvent signal as standard ($CDCl_3$, δ 7.26; D_2O, δ 4.79; CD_3OD, δ 3.31; CD_3CN, δ 1.93).

$^{13}C\{^1H\}$-NMR spectra were recorded with ^1H-decoupling on Bruker 101 MHz, Bruker 126 MHz (with cryoprobe) and Bruker 126 MHz (without cryoprobe) spectrometers at 298 K in the indicated deuterated solvent. All signals were referenced to the internal solvent signal as standard ($CDCl_3$, δ 77.2; CD_3OD, δ 49.0; CD_3CN, δ 1.32).

$^{31}P\{^1H\}$-NMR spectra and ^{31}P-NMR spectra were recorded with ^1H-decoupling or ^1H coupling, respectively, on Bruker 202 MHz and Bruker 122 MHz spectrometers in the indicated deuterated solvent. All signals were referenced to an internal standard (PPP).

Mass spectra were recorded by the Mass spectrometry service of University of Zurich and University of Freiburg on Finnigan MAT 95 MS, Bruker Esquire LC MS, Bruker maXis QTof HRMS, Finnigan TSQ 700 MS and Thermo Scientific EXACTIVE spectrometer with Orbitrap analyzer.

Synthesis of (9*H*-Fluoren-9-yl)methyl ((7-(diethylamino)-2-oxo-2*H*-chromen-4-yl)methyl) diisopropylphosphoramidite

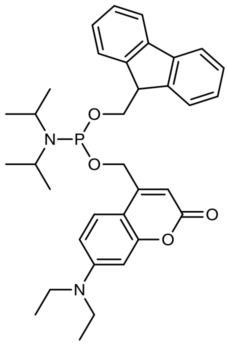

The compound was synthesized as described before starting from 1-((9H-fluoren-9-yl)methoxy)-*N*,*N*,*N'*,*N'*-tetraisopropylphosphanediamine and (7-(diethylamino)-2-oxo-2*H*-chromen-4-yl)methanol (DEACM-OH).
The analytical data were identical with the literature.[6]

Synthesis of 6

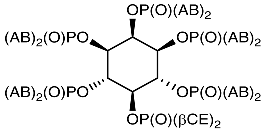

The compound was synthesized as described before in five steps starting from *myo*-inositol. The Analytical data were identical with the literature.[7]

General procedure 1 (GP-1): Synthesis of *meso*-phosphoanhydrides

1.00 eq. hexakisphosphate (**6**) was dissolved in dry CH_3CN (2 mL), 4.00 eq. DBU was added followed by addition of 4.00 eq. BSTFA. The solution was stirred 15 minutes at room temperature. Reaction was monitored by TLC. After completion of the deprotection a mixture of 4.00 eq. MeOH and 4.0 eq. TFA were added to the solution. The mixture was stirred for 10 min and then evaporated to dryness. The residue was taken up in dry CH_3CN (2 mL), 2.00 eq. of (9*H*-Fluoren-9-yl)methyl ((7-(diethylamino)-2-oxo-2*H*-chromen-4-yl)methyl) diisopropylphosphoramidite and 2.00 eq. 1*H*-tetrazole (0.45 M in CH_3CN) were further added. This solution was stirred for 15 minutes at room temperature and then cooled down to 0 °C. Progress of the reaction was monitored by ^{31}P NMR. After completion of the reaction, oxidation was achieved by slow (!) addition of 2.0 eq. *m*CPBA (70% moistened with water). The reaction mixture was precipitated with Et_2O and pure product was obtained and dried *in vacuo*.

Synthesis of 7

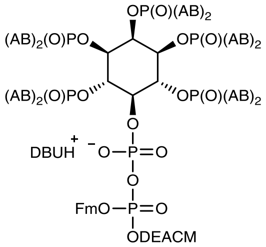

Product **7** was synthetized according to the general procedure GP-1. 50 mg (22 µmol, 1.0 eq.) hexaphosphate **6**, 13.5 µL (13.7 mg, 88 µmol, 4.0 eq.) of DBU, 23.8 µL (22.8 mg, 88 µmol, 4.0 eq.) BSTFA, 24.0 µL MeOH and 6.96 µL (10.4 mg, 88 µmol, 4.0 eq.) TFA, 22.8 mg (44 µmol, 2.0 eq.) ((7-(diethylamino)-2-oxo-2*H*-chromen-4-yl)methyl)

diisopropylphosphoramidite, 100 μL (44 μmol, 2.0 eq) 1*H*-tetrazole (0.45 M in CH_3CN) 15.0 mg (44 μmol, 2.0 eq.) *m*CPBA (70% moistened with water). Isolated yield: 72% (44.0 mg, 15.8 μmol).

^{1}**H NMR** (400 MHz, $CDCl_3$): δ 10.93 (DBU), 7.75-7.47 (m, 5H), 7.29-7.17 (m, 27H), 6.79-6.91 (m, 20H), 5.19-5.03 (m, 24H), 4.53-4.13 (m, 6H), 3.40-3.34 (DBU), 2.68 (DBU), 2.28 (t, *J* = 7.8 Hz, 30H), 2.06 (DBU), 1.86 (DBU), 1.61 (DBU), 1.29-1.18 (DBU); ^{13}**C NMR** (101 MHz, $CDCl_3$): δ 169.2, 166.2, 162.2, 161.9, 156.0, 150.7-150.6 (m), 144.3, 143.6, 143.3, 143.2, 141.34, 141.25, 141.18, 133.8-133.1 (m), 129.2, 128.1, 127.9-127.1 (m), 126.9, 125.3, 125.2, 124.7-121.6 (m), 119.8, 108.7, 105.6, 105.5, 97.4, 75.4, 73.8, 69.5, 69.3, 69.2, 64.6, 60.4, 54.4, 53.5, 48.5, 47.9, 44.71, 44.65, 38.1, 32.4, 28.9, 26.6, 25.9, 23.8, 21.1, 19.3, 18.9, 14.2, 12.4; ^{31}**P{^{1}H}NMR** (162 MHz, $CDCl_3$): δ 0.34 (br s, 2P) , − 0.48 (s, 2P) , − 1.62 (br s, 1P), − 9.80 (d, *J* = 12.6 Hz, 1P), − 11.79 (d, *J* = 13.6 Hz, 1P); ^{31}**P NMR** (162 MHz, $CDCl_3$) δ 0.42 (br s, 2P), − 0.47 (br s, 2P), − 1.60 (br s, 1P), − 9.80 (br s, 1P), − 11.83 (d, *J* = 11.0 Hz, 1P); **HRMS** (ESI) $[M-H]^-$ calcd. for $C_{124}H_{123}NO_{49}P_7$: 2627.5405, found: 2627.5356.
Note: for HRMS, M corresponds to “all protonated” form.

Synthesis of 8

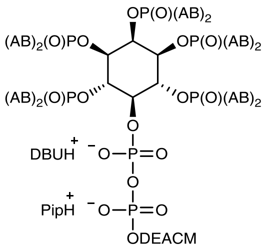

25 mg (8.99 μmol, 1.0 eq.) **7** was dissolved in DMF (1 mL) and 1.1 μL piperidine (10.79 μmol, 1.2 eq.) was added. The solution was stirred for 10 minutes at room temperature. After completion of the deprotection, the solution was concentrated under reduced pressure and the product was precipitated with Et_2O (5 mL). The precipitate was centrifuged and separated by decantation of the solvent. The precipitate was once more dissolved in CH_2Cl_2/MeOH and crystallized by addition of Et_2O. Isolated yield: 62% (15 mg, 5.58 mmol)
Note: an alternate purification by MPLC (C4 column, H_2O/CH_3CN/TEAA eluent) reduces the yield to 32%.

^{1}H-NMR (500 MHz, $CDCl_3$): δ 11.36 (DBU), 8.72 (piperidine), 8.03 (DBU), 7.29-7.21 (m, 24H), 6.92-6.90 (m, 20H), 5.19-5.03 (m, 22H), 3.47-3.31 (DBU/piperidine), 2.97 (DBU/piperidine), 2.89 (DBU/piperidine), 2.72 (t, *J* = 5.3 Hz, 4H), 2.27 (t, *J* = 3.3 Hz, 30H), 2.18 (piperidine), 1.88 (br s, *J* = 5.8 Hz, 6H), 1.64-1.15 (DBU/piperidine); **^{13}C-NMR** (126 MHz, $CDCl_3$): δ 206.9, 169.4, 169.22, 169.16, 166.2, 162.6, 162.5, 162.4, 162.1, 161.9, 159.4, 156.0, 155.9, 150.69, 150.66, 150.61, 150.55, 150.50, 150.44, 150.38, 149.9, 133.5, 133.2, 133.1, 132.7, 129.3, 129.23, 129.20, 129.18, 128.7, 128.5, 121.7, 121.66, 121.63, 121.59, 121.3, 121.1, 121.0, 119.7, 118.8, 118.2, 116.3, 115.9, 108.7, 107.8, 106.3, 106.2, 105.52, 105.45, 105.36, 97.43, 97.36, 69.3, 54.3, 53.4, 48.6, 44.6, 44.3, 38.0, 36.5, 32.3, 31.4, 30.9, 28.9, 26.7, 23.9, 22.6, 22.5, 21.1, 19.4, 12.4; **^{31}P{^{1}H}NMR** (202 MHz, $CDCl_3$): δ 0.14 (br s, 3P), − 0.62 (br s, 2P), − 10.06 (d, *J* = 12.8 Hz, 1P), − 11.21 (d, *J* = 10.8 Hz, 1P); **^{31}P-NMR** (202 MHz, $CDCl_3$) δ 0.14 (m, 3P), − 0.62 (m, 2P), − 10.02 (m, 1P), − 11.12 (m, 1P); **HRMS** (ESI) $[M{-}H]^-$ calcd. for $C_{110}H_{113}NO_{49}P_7$: 2449.4623, found: 2449.4560.

General procedure 2 (GP-2): Synthesis of asymmetric hexaphosphates

1.0 eq. of diastereopure inositol monophosphate **10** (or *dias*-**10**) and 10.0 eq. of acyloxybenzyl phosphoramidite were coevaporated twice with dry acetonitrile (2 mL). Afterwards, the residue was dissolved in dry DMF (5 mL). To this solution 10.0 eq. of 5-(Ethylthio)-1*H*-tetrazole (ETT) was added. Progress of the reaction was monitored by ^{31}P-NMR. After completion of the reaction (~ 1 h 15 min), oxidation was achieved by slow (!) addition of 10.0 eq. *m*CPBA (77% moistened with water) at 0 °C. The reaction mixture was concentrated in *vacuo*. The product was purified by silica gel flash chromatography (Ethyl acetate/Toluene 4:1) to obtain pure **11** (or *dias*-**11**) as a white semi-solid.

Synthesis of 11

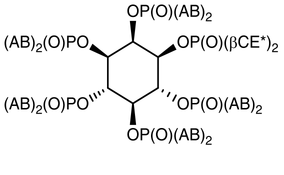

Compound **11** was synthetized according to the general procedure GP-2.

144.0 mg (0.278 mmol, 1.0 eq.) diastereopure inositol monophosphate **10**, 1.28 g (2.780 mmol, 10.0 eq.) acyloxybenzyl phosphoramidite, 361.9 mg (2.780 mmol, 10.0 eq.) ETT, 623.0 mg (2.78 mmol, 10.0 eq.) *m*CPBA (77% moistened with water). Isolated yield: 51% (340.0 mg, 0.142 mmol).

^{1}**H NMR** (400 MHz, Acetonitrile-d_3) δ 7.37-7.12 (m, 30H), 7.05-6.89 (m, 20H), 5.87-5.73 (m, 1H), 5.73-5.64 (m, 1H), 5.22-4.52 (m, 26H), 3.18-3.01 (m, 2H), 3.00-2.73 (m, 2H), 2.23-2.20 (m, 30H); ^{13}**C NMR** (101 MHz, CD_3CN) δ 170.42, 170.39, 152.0, 151.99, 151.94, 151.89, 151.86, 138.24, 138.19, 134.79, 134.77, 134.72, 134.68, 134.65, 134.62, 134.56, 134.51, 134.48, 134.45, 134.42, 130.40, 130.35, 130.28, 130.26, 130.24, 130.20, 130.0, 129.8, 129.7, 127.6, 127.0, 122.99, 122.96, 122.92, 122.86, 122.83, 118.3, 117.5, 117.2, 77.0, 76.9, 76.25, 76.20, 75.8, 74.5, 74.0, 70.4, 70.3, 70.24, 70.19, 70.13, 70.0, 69.95, 69.87, 60.9, 27.71, 27.66, 27.3, 27.2, 21.2; ^{31}**P{^{1}H}NMR** (162 MHz, Acetonitrile-d_3) δ − 0.74 (s, 1P), − 0.85 (s, 1P), − 0.90 (s, 1P), − 1.54 (s, 1P), − 2.94 (s, 1P), − 4.54 (s, 1P); ^{31}**P NMR** (162 MHz, Acetonitrile-d_3) δ − 0.82 (tt, *J* = 17.8, 9.6 Hz, 3P), − 1.57 (dt, *J* = 16.5, 8.3 Hz, 1P), − 2.94 (q, *J* = 7.8 Hz, 1P), − 4.54 (q, *J* = 8.4 Hz, 1P); **HRMS** (ESI) $[M+Na]^+$ calcd. for $C_{114}H_{112}N_2NaO_{44}P_6$: 2422.4945, found: 2422.4939.

Synthesis of *dias*-11

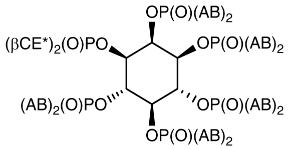

Compound *dias*-**11** was synthetized and isolated according to the general procedure GP-2.

114.0 mg (0.219 mmol, 1.0 eq.) diastereopure inositol monophosphate *dias*-**10**, 1.01 g (2.19 mmol, 10.0 eq.) acyloxybenzyl phosphoramidite, 285.0 mg (2.19 mmol, 10.0 eq.) ETT, 490.8 mg (2.19 mmol, 10.0 eq.) *m*CPBA (77% moistened with water). Isolated yield: 48% (253.3 mg, 0.105 mmol).

^{1}**H NMR** (500 MHz, Methanol-d_4) δ 7.44-6.90 (m, 50H), 5.80-5.75 (m, 1H), 5.60-5.56 (m, 1H), 5.15-4.87 (m, 22H), 4.80-4.76 (m, 3H), 4.65 (q, *J* = 9.8 Hz, 1H), 3.18-3.04 (m, 2H), 2.90-2.76 (m, 2H), 2.26-2.22 (m, 30H); ^{13}**C NMR** (126 MHz, Methanol-d_4) δ 172.9, 170.99-

170.92 (m), 152.53-152.50 (m), 138.2 (d), 138.1 (d), 134.6-134.4 (m), 130.8-130.5 (m), 130.3, 130.1, 129.8, 127.9, 127.4, 123.2-122.9, 118.1, 117.4, 77.9 (d), 77.3 (d), 76.3, 74.7, 74.4, 70.9-70.6 (m), 61.5, 27.4 (m), 20.9, 14.5; ^{31}**P{^{1}H}NMR** (202 MHz, Methanol-*d*$_4$) δ − 1.00 (s, 1P) , − 1.29 (s, 1P), − 1.55 (s, 1P), − 1.96 (s, 1P), − 2.97 (s, 1P), − 4.34 (s, 1P); **HRMS** (ESI) $[M+Na]^+$ calcd. for $C_{114}H_{112}N_2NaO_{44}P_6$: 2422.4945, found: 2422.4939.

General procedure 1´ (GP-1´): Synthesis of asymmetric phosphoanhydrides

The general procedure GP-1 was slightly modified, ETT (2.0 eq.) was used as an activator instead of 1*H*-tetrazole. For work up, after precipitation by Et_2O, the dark red viscous oil was reprecipitated from a mixture of CH_2Cl_2/Et_2O. This final reddish-yellow compound contained a crude mixture of two diastereomers (formation of phosphoanhydride generates a new stereogenic center at the phosphorus, marked as *), which was subjected to the subsequent steps without further purification.

Synthesis of 12

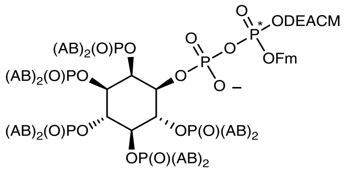

Compound **12** was synthetized according to the general procedure GP-1´.

165.0 mg (0.069 mmol, 1.0 eq.) hexaphosphate **11**, 41.0 µL (0.276 mmol, 4.0 eq.) dry DBU, 73.5 µL (0.276 mmol, 4.0 eq.) BSTFA, 21.0 µL (0.276 mmol, 4.0 eq.) TFA, 69.4 µL MeOH, 79.1 mg (0.138 mmol, 2.0 eq.) ((7-(diethylamino)-2-oxo-2*H*-chromen-4-yl)methyl) diisopropylphosphoramidite, 19.0 mg (0.138 mmol, 2.0 eq.) ETT, 30.9 mg (0.138 mmol, 2.0 eq.) *m*CPBA (77% moistened with water). Crude product: 235.0 mg.

^{31}**P{^{1}H}NMR** (122 MHz, Acetonitrile-d$_3$) δ − 0.72 (s, 1P), − 0.89 (s, 1P), − 1.09 (d, *J* = 4.4 Hz, 1P), − 1.39 (d, *J* = 4.4 Hz, 1P), − 2.85 (d, *J* = 8.4 Hz, 1P), − 11.92 (dd, *J* = 24.0, 15.1 Hz, 1P), − 13.83 (dd, *J* = 15.6, 5.5 Hz, 1P); **HRMS** (ESI) $[M-H]^-$ calcd. for $C_{124}H_{123}NO_{49}P_7$: 2627.5366, found 2627.5354.

Synthesis of *dias*-12

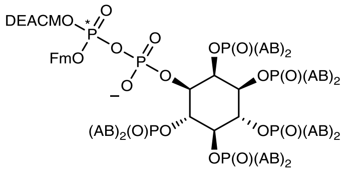

Compound *dias*-**12** was synthetized according to the general procedure GP-1´.

68.0 mg (0.028 mmol, 1.0 eq.) hexaphosphate *dias*-**11**, 16.9 µL (0.113 mmol, 4.0 eq.) dry DBU, 30.3 µL (0.113 mmol, 4.0 eq.) BSTFA, 8.65 µL (0.113 mmol, 4.0 eq.) TFA, 28.6 µL MeOH, 32.6 mg (0.057 mmol, 2.0 eq.) ((7-(diethylamino)-2-oxo-2*H*-chromen-4-yl)methyl) diisopropylphosphoramidite, 7.8 mg (0.057 mmol, 2.0 eq.) ETT, 12.8 mg (0.057 mmol, 2.0 eq.) *m*CPBA (77% moistened with water). Crude product: 100.0 mg.

^{31}P{^{1}H}NMR (202 MHz, Acetonitrile-d_3) δ − 0.77 (s, 1P), − 0.95 (d, *J* = 4.94 Hz, 1P) , − 1.13 (d, *J* = 4.9 Hz, 1P), − 1.49 (s, 1P), − 2.91 (d, *J* = 7.4 Hz, 1P), − 11.62 (dd, *J* = 37.3, 16.5 Hz, 1P), − 12.27 (d, *J* = 16.2 Hz, 1P); **HRMS** (ESI) $[M{-}H]^-$ calcd. for $C_{124}H_{123}NO_{49}P_7$: 2627.5366, found: 2627.5354.

General procedure 3 (GP-3): Deprotection of the Fm group

1.0 eq. of **12** (or *dias*-**12**) was dissolved in DMF and 1.2 eq. of piperidine was added. The solution was stirred for 4 minutes at room temperature. The crude product was then precipitated by addition of excess Et_2O. The precipitate was centrifuged and separated by decantation of solvent. The crude dark brown viscous oil was then purified on a KNAUER AZURA preparative RP-HPLC (Mobile phase: H_2O/CH_3CN gradient with isocratic 10 mM TEAA buffer) equipped with a Hypersil GOLD C4 Column (5 µ, 21.2 x 250 mm) and coupled to a SEDERE SEDEX (model LC) LT-ELSD detector.

Synthesis of 13 and *ent*-13

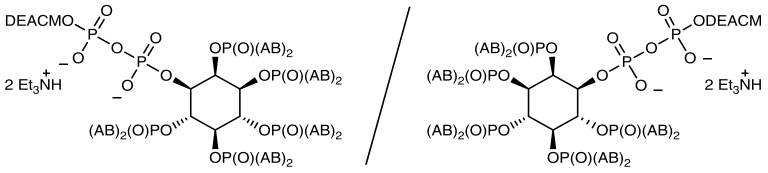

Products **13** and *ent*-**13** were synthetized according to the general procedure GP-3.

100.0 mg (35.95 µmol, 1.0 eq.) *dias*-**12**, 2.5 mL DMF, 4.3 µL piperidine (43.14 µmol, 1.2 eq.). Isolated yield *ent*-**13**: 25% (4 steps; 18.8 mg, 7.09 µmol).

118.0 mg (42.43 µmol, 1.0 eq.) **12**, 2.5 mL DMF, 5.0 µL (50.92 µmol, 1.2 eq.) piperidine. Isolated yield **13**: 23% (4 steps; 21.0 mg, 7.91 µmol).

^{1}H NMR (500 MHz, Acetonitrile-*d*$_3$) δ 7.49-6.86 (m, 43H), 6.41-6.14 (m, 1H), 5.42-4.85 (m, 28H), 2.89 (q, *J* = 7.3 Hz, 16H), 2.21-2.19 (m, 30H), 1.09 (t, *J* = 7.3 Hz, 24H); **^{13}C NMR** (126 MHz, CD_3CN) δ 173.7, 170.42-170.38 (m), 162.7, 157.1, 154.83, 154.77, 151.9, 151.81-151.78 (m), 151.6, 151.5, 135.6 (d), 135.3-135.2 (m), 134.9-134.7, 130.5, 130.4-130.1 (m), 129.4, 126.6, 123.7, 122.8-122.3 (m), 118.3, 109.5, 107.1, 106.2, 97.9, 78.5, 76.8, 76.3, 74.8, 72.1, 70.3-69.7 (m), 64.3 (d), 53.4, 46.3, 45.2, 30.9, 21.7, 21.2, 12.8, 9.3, 8.1; **^{31}P{^{1}H} NMR** (202 MHz, Acetonitrile-*d*$_3$) δ − 0.72 (s, 1P), − 0.96 (s, 1P), − 1.07 (s, 1P), − 1.50 (s, 1P), − 3.16 (s, 1P), − 11.11 (d, *J* = 15.1 Hz, 1P), − 11.78 (d, *J* = 15.2 Hz, 1P); **HRMS** (ESI) $[M-2H]^{2-}$ calcd. for $C_{110}H_{112}NO_{49}P_7$: 1224.2255, found: 1224.2255. The analytical data obtained for **13**, *ent*-**13** were identical in all respects (besides minor concentration dependent shifts in the NMR spectra), except for the inverted optical rotations.
$[\alpha]_D^{20}$ = + 11.5 (**13**, C 0.600, $CHCl_3$). **$[\alpha]_D^{20}$** = − 12.0 (*ent*-**13**, C 0.083, $CHCl_3$).

General procedure 4 (GP-4): Global deprotection

AB-protected photocaged **13** (or *ent*-**13**) was dissolved in DMF and piperidine (33%) was added. The solution was stirred for 90 minutes at room temperature. After completion of the deprotection, the product was precipitated by addition of excess Et_2O. The precipitate was centrifuged and the collected solid was redissolved in MeOH; NaI was added into this solution to exchange the piperidinium counter ions. After 30 minutes of stirring at room

temperature, sodium salt of the final compound was precipitated from the solution, which was further centrifuged, washed with additional MeOH and finally collected as yellow solids.

Synthesis of 14 and *ent*-14

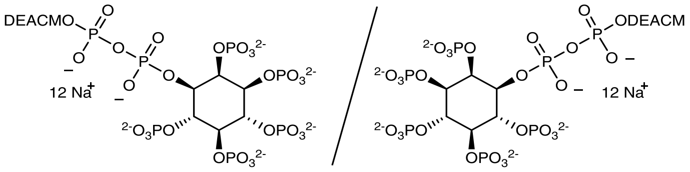

Products **14** and *ent*-**14** were synthetized according to the general procedure GP-4.

18.0 mg (6.78 µmol, 1.0 eq.) **13**, 1.5 mL DMF, 0.75 mL piperidine, 1.0 mL MeOH, 15.5 mg NaI. Isolated yield: 62% (5.2 mg, 4.22 µmol).
9.0 mg (3.39 µmol, 1.0 eq.) *ent*-**13**, 0.8 mL DMF, 0.4 mL piperidine, 0.5 mL MeOH, 7.8 mg NaI. Isolated yield: 62% (2.6 mg, 2.11 µmol)

^{1}H NMR (300 MHz, D_2O) δ 7.97-7.81 (m, 1H), 7.64-7.56 (m, 1H), 6.84-6.73 (m, 1H), 6.62-6.27 (m, 1H), 5.32 (d, *J* = 6.4 Hz, 1H), 5.22 (*J* = 6.6 Hz, 1H), 4.98 (d, *J* = 9.8 Hz, 1H), 4.39 (p, *J* = 9.9 Hz, 2H), 4.13 (dt, *J* = 18.9, 10.5 Hz, 3H), 3.93-3.84 (m, 1H), 3.72-3.59 (m, 1H), 3.39 (q, *J* = 7.1 Hz, 2H), 1.06 (dt, *J* = 25.0, 7.0 Hz, 6H); **^{31}P{^{1}H} NMR** (122 MHz, D_2O) δ 2.54 (s, 1P), 1.13-0.69 (m, 4P), − 10.54 (br s, 2P); **HRMS** (ESI) $[M{-}H]^-$ calcd. for $C_{20}H_{33}NO_{29}P_7$: 967.9307, found: 967.9315.

General procedure 5 (GP-5): Photo-uncaging

Photocaged **14** (or *ent*-**14)** were dissolved in H_2O and taken into a quartz tube which was placed inside a Rayonet photoreactor. The solution was then irradiated at 366 nm for 10 minutes. Afterwards, the tube was taken out and put it on a gentle vortex for 2 minutes. Afterwards, the tube was put back inside the reactor and this cycle was continued for 3 more times. The progress of the reaction was monitored by HPLC traces. After completion of the photo-uncaging, products were precipitated by addition of MeOH, which was then centrifuged and further washed with additional MeOH. The final products were collected as white solids.

Synthesis of 3 and *ent*-3

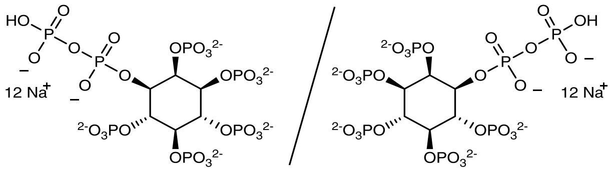

Compounds **3** and *ent*-**3** were synthetized according to the general procedure GP-5.

5.0 mg (4.05 µmol) **14**, 0.5 mL H_2O. Isolated yield: 80% (3.18 mg, 3.24 µmol).
3.0 mg (2.43 µmol) *ent*-**14**, 0.5 mL H_2O. Isolated yield: 80% (1.90 mg, 1.94 µmol).
The analytical data for **3** and *ent*-**3** were identical with the literature.[8]

Synthesis of phosphate triester 15 (negative control)

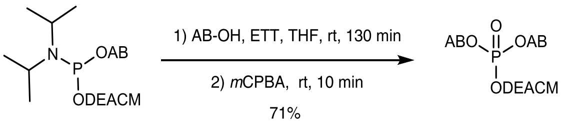

Bis(diisopropyl)(AB)(DEACM) P-amidite (27.0 mg, 50.0 µmol, 1.0 eq.) was coevaporated with dry MeCN (2.0 mL) and dissolved in dry THF (1.0 mL). AB-OH (13.0 mg, 78.3 µmol, 1.6 eq.) and ETT (0.77 M, 130 µL, 100 µmol, 2.0 eq.) were added and the mixture was stirred at room temperature. After 105 min more ETT (0.77 M, 65 µL, 50.0 µmol, 1.0 eq.) was added and after further 25 min *m*CPBA (77%, 13.5 mg, 60.0 µmol, 1.5 eq.) was added. After another 10 min the solvent was removed under reduced pressure and the crude product was purified by flash chromatography (SiO_2, from Et_2O to Et_2O/MeOH 20:1) to obtain the desired $(AB)_2$(DEACM) phosphate triester **15** (22.0 mg, 35.3 µmol, 71%) as yellow sticky wax.

^{1}H-NMR (400 MHz, $CDCl_3$) δ = 7.35 (d, *J* = 8.5 Hz, 4H), 7.18 (d, *J* = 8.9 Hz, 1H), 7.08 (d, *J* = 8.5 Hz, 4H), 6.53 (dd, *J* = 9.0, 2.6 Hz, 1H), 6.49 (d, *J* = 2.6 Hz, 1H), 6.10 (t, *J* = 1.2 Hz, 1H), 5.08 – 5.02 (m, 6H), 3.40 (q, *J* = 7.1 Hz, 4H), 2.29 (s, 6H), 1.20 (t, *J* = 7.1 Hz, 6H).; **^{13}C-NMR** (101 MHz, $CDCl_3$) δ = 169.3, 161.8, 156.3, 151.0, 150.8, 149.0 (d, *J* = 8.1 Hz), 133.0 (d, *J* = 6.6 Hz), 129.4, 124.4, 122.0, 108.8, 106.7, 105.6, 97.9, 69.3 (d, *J* = 5.8 Hz), 64.7 (d, *J* = 4.6 Hz), 44.8, 21.2, 12.5; **^{31}P{^{1}H}-NMR** (121 MHz, $CDCl_3$) δ = −1.0; **HRMS** (ESI) $[M+Na^+]^+$ calcd. for $C_{32}H_{34}NNaO_{10}P$: 646.1813, found: 646.1805.

References

1. Miyazaki, J-I. *et al*. Establishment of a pancreatic β cell line that retains glucose-inducible insulin secretion: special reference to expression of glucose transporter isoforms. *Endocrinology* **127(1)**, 126–132 (1990).
2. Losito, O., Szijgyarto, Z., Resnick, A. C. & Saiardi, A. Inositol pyrophosphates and Their Unique Metabolic Complexity: Analysis by Gel electrophoresis. *PLoS One* **4**, e5580 (2009).
3. Wilson, M. S. C. *et al*. A novel method for the purification of inositol phosphates from biological samples reveals that no phytate is present in human plasma or urine *Open Biol.* **5:** 150013 (2018).
4. Wilson, M. S. C., Saiardi, A. Inositol Phosphate Purification Using Titanium Dioxide Beads. *Bio Protoc.* **8(15)**, e2959 (2018).
5. Schindelin, J. *et al*. Fiji: an open-source platform for biological-image analysis. *Nat Methods* **9(7)**, 676–682 (2012).
6. Subramanian, D. *et al*. Activation of membrane-permeant caged ptdins(3)p induces endosomal fusion in cells. *Nat Chem Biol* **6**, 324 – 326 (2010).
7. Pavlovic, I. *et al*. Prometabolites of 5-Diphospho-*myo*-inositol Pentakisphosphate. *Angew. Chem. Int. Ed.* **54**, 9622 – 9626 (2015).
8. Capolicchio, S., Thakor, D. T., Linden, A. & Jessen, H. J. Synthesis of Unsymmetric Diphospho-Inositol Polyphosphates. *Angew.* Chem. *Int. Ed.* **52** , 6912 – 6916 (2013).

Compound 7, 1H

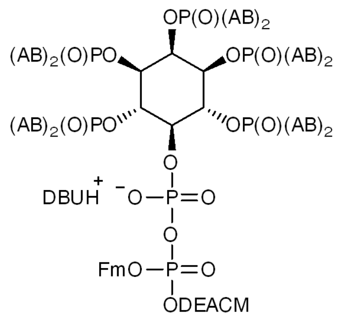

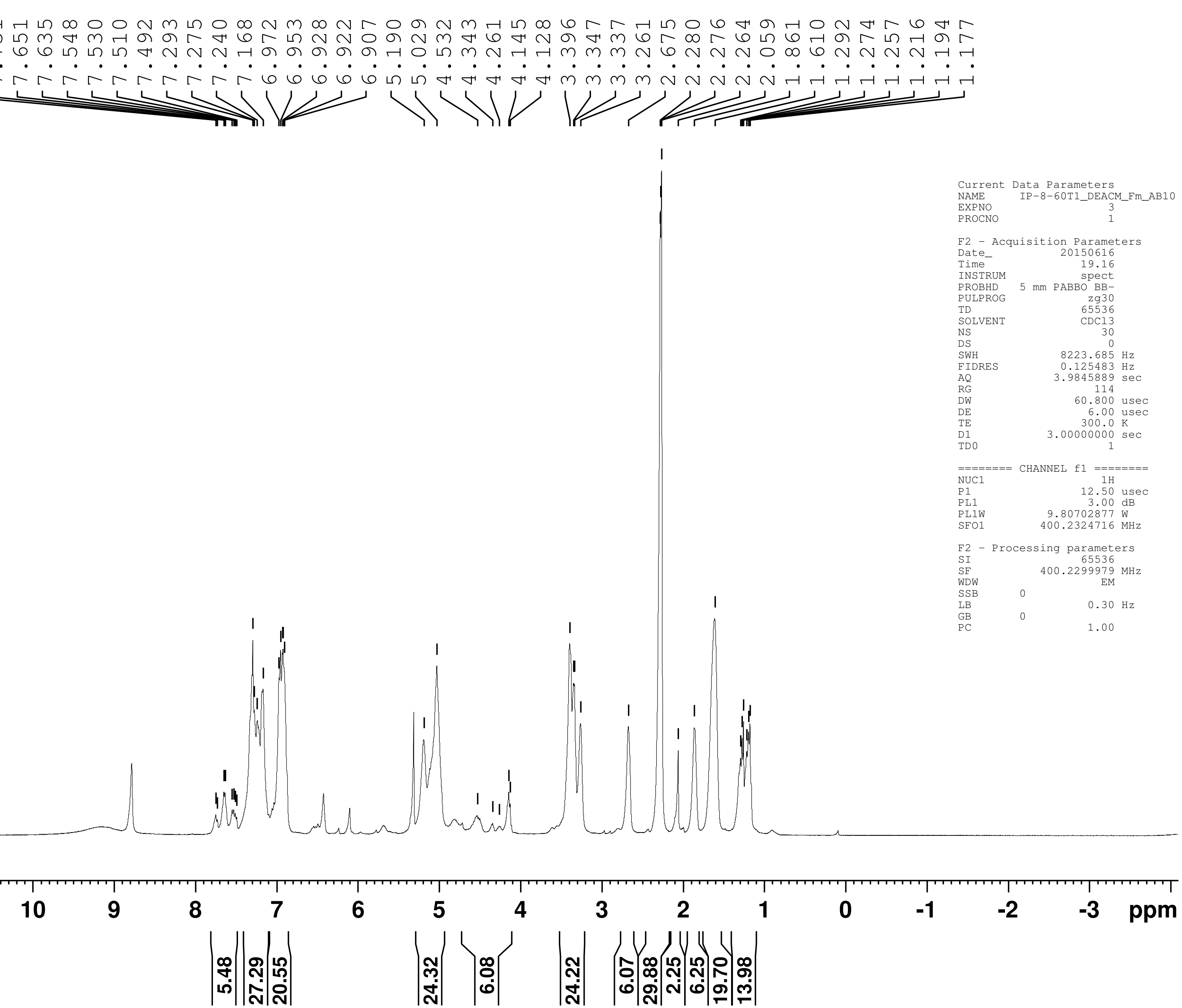

Current Data Parameters
NAME IP-8-60T1_DEACM_Fm_AB10
EXPNO 3
PROCNO 1

F2 - Acquisition Parameters
Date_ 20150616
Time 19.16
INSTRUM spect
PROBHD 5 mm PABBO BB-
PULPROG zg30
TD 65536
SOLVENT CDCl3
NS 30
DS 0
SWH 8223.685 Hz
FIDRES 0.125483 Hz
AQ 3.9845889 sec
RG 114
DW 60.800 usec
DE 6.00 usec
TE 300.0 K
D1 3.00000000 sec
TD0 1

======== CHANNEL f1 ========
NUC1 1H
P1 12.50 usec
PL1 3.00 dB
PL1W 9.80702877 W
SFO1 400.2324716 MHz

F2 - Processing parameters
SI 65536
SF 400.2299979 MHz
WDW EM
SSB 0
LB 0.30 Hz
GB 0
PC 1.00

Compound 7, 13C{1H}

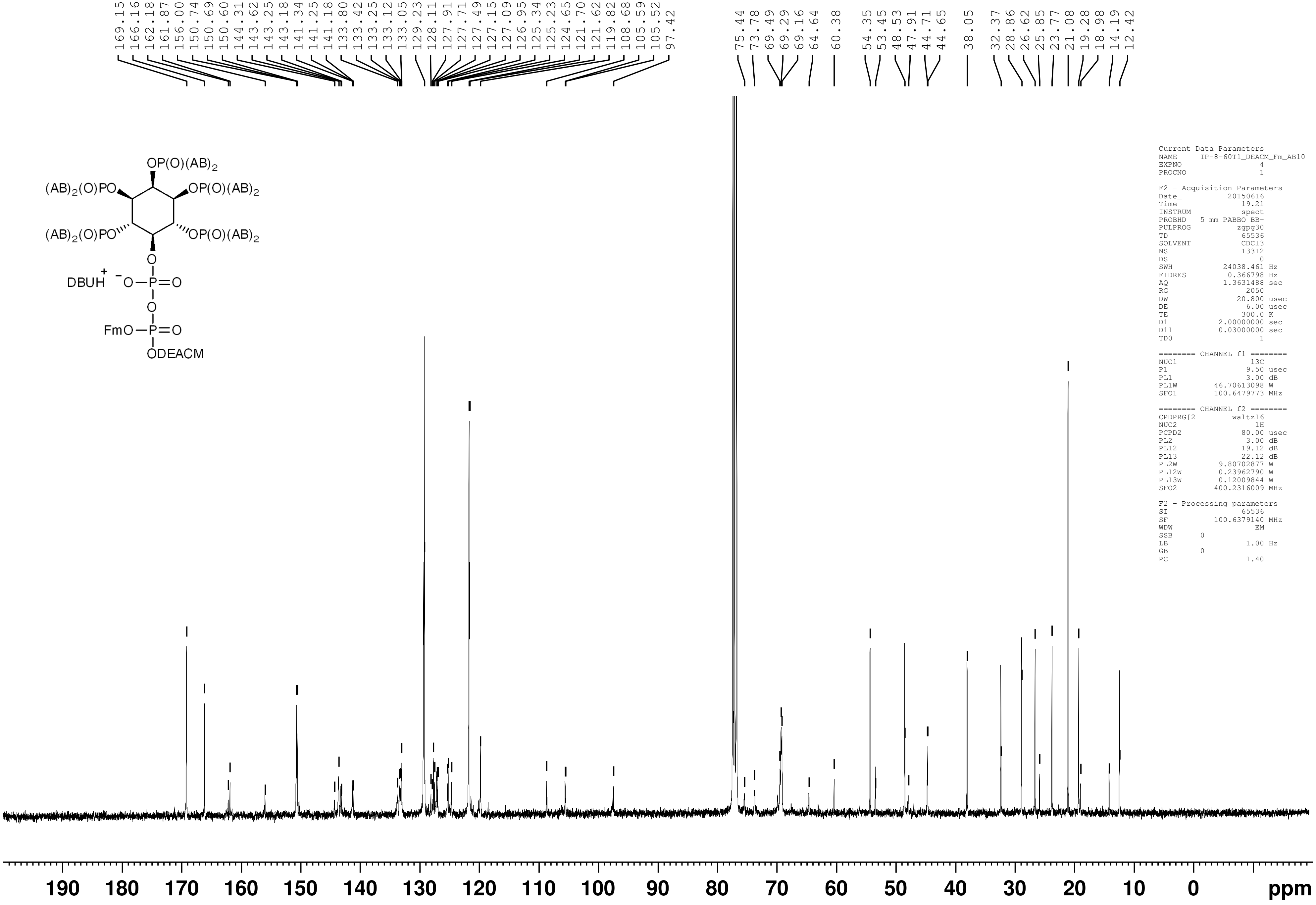

Compound 7, 31P{1H}

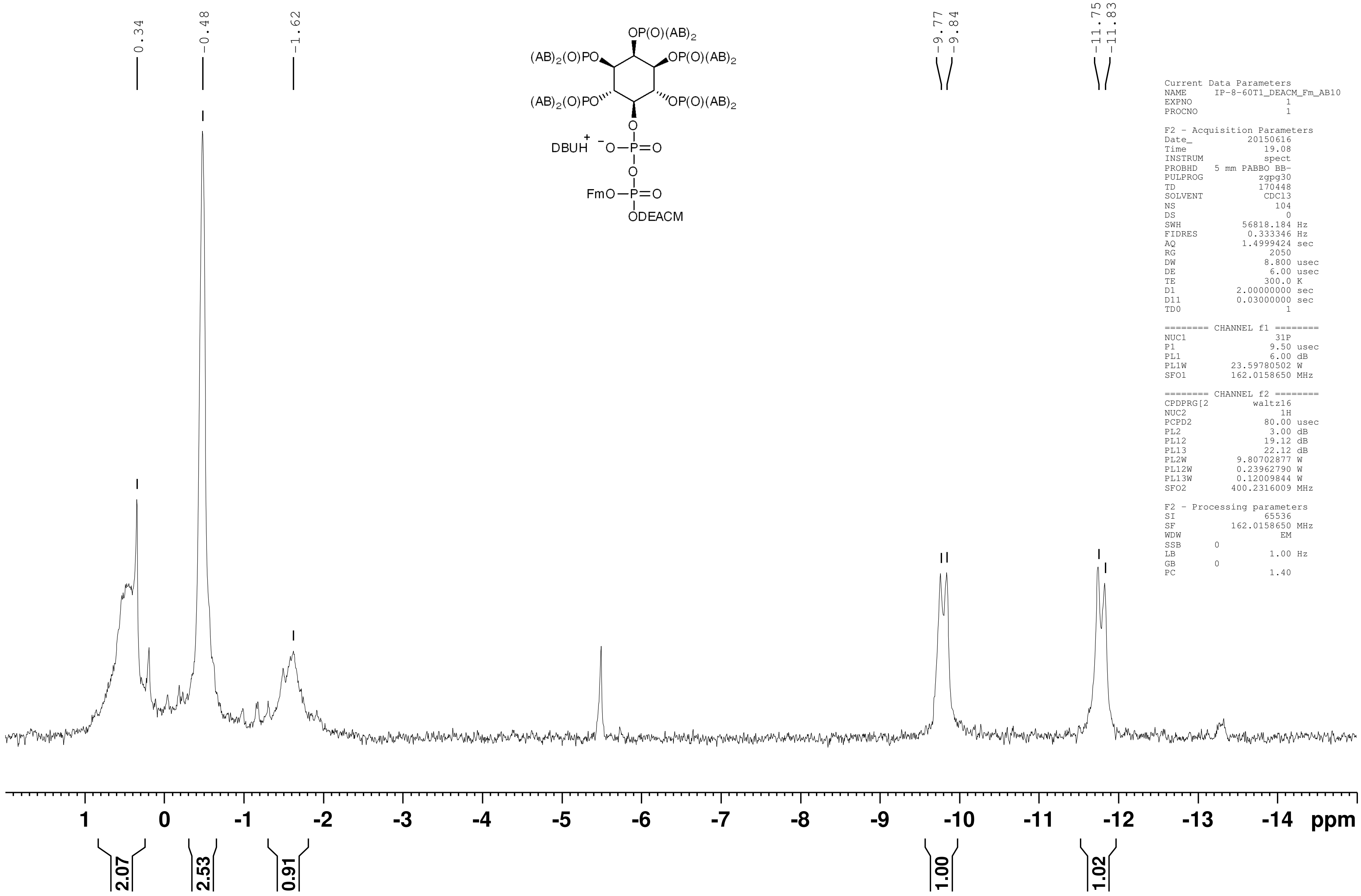

Current Data Parameters
NAME IP-8-60T1_DEACM_Fm_AB10
EXPNO 1
PROCNO 1

F2 - Acquisition Parameters
Date_ 20150616
Time 19.08
INSTRUM spect
PROBHD 5 mm PABBO BB-
PULPROG zgpg30
TD 170448
SOLVENT CDCl3
NS 104
DS 0
SWH 56818.184 Hz
FIDRES 0.333346 Hz
AQ 1.4999424 sec
RG 2050
DW 8.800 usec
DE 6.00 usec
TE 300.0 K
D1 2.00000000 sec
D11 0.03000000 sec
TD0 1

======== CHANNEL f1 ========
NUC1 31P
P1 9.50 usec
PL1 6.00 dB
PL1W 23.59780502 W
SFO1 162.0158650 MHz

======== CHANNEL f2 ========
CPDPRG[2 waltz16
NUC2 1H
PCPD2 80.00 usec
PL2 3.00 dB
PL12 19.12 dB
PL13 22.12 dB
PL2W 9.80702877 W
PL12W 0.23962790 W
PL13W 0.12009844 W
SFO2 400.2316009 MHz

F2 - Processing parameters
SI 65536
SF 162.0158650 MHz
WDW EM
SSB 0
LB 1.00 Hz
GB 0
PC 1.40

Compound 7, 31P

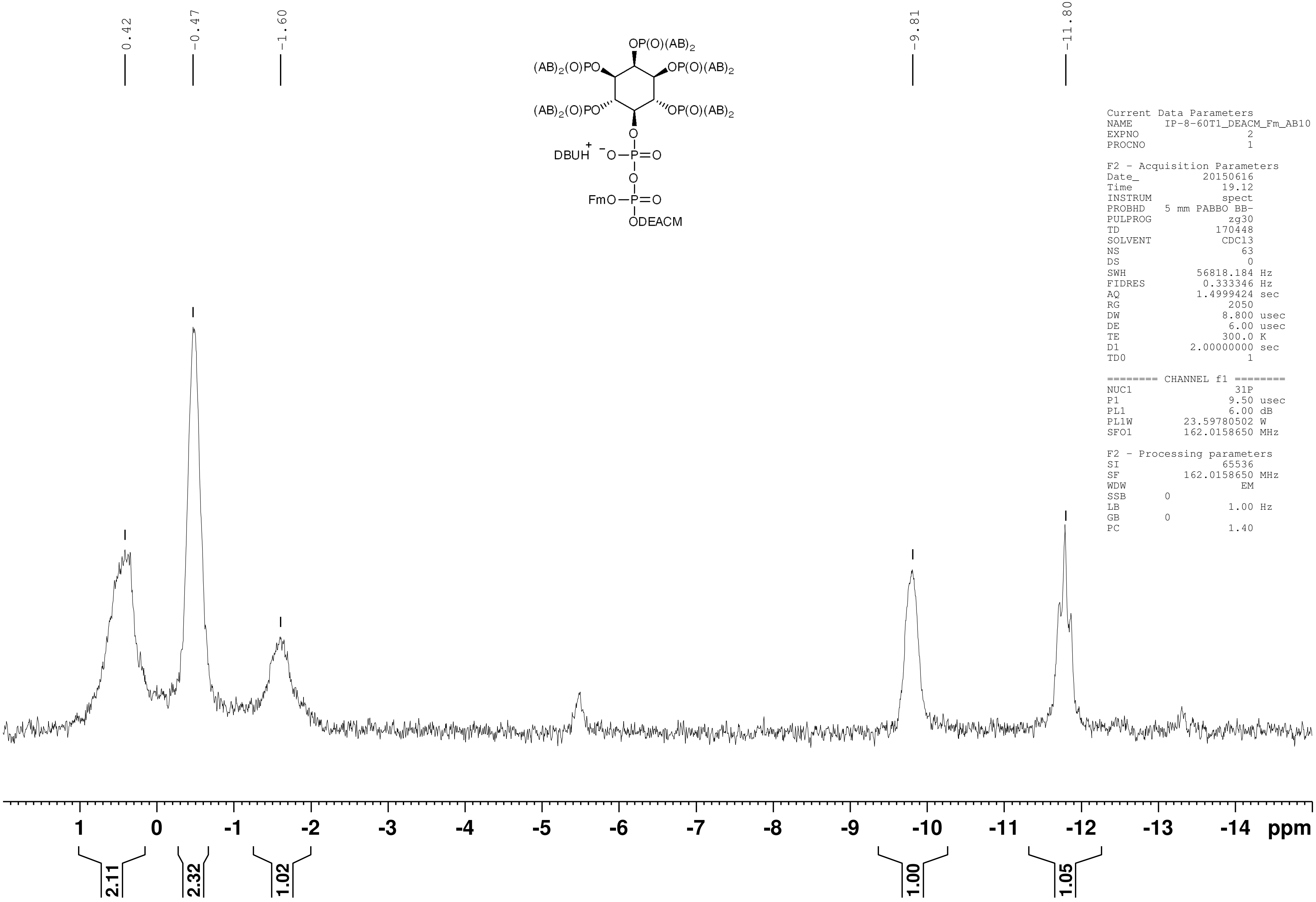

Compound 8, 1H

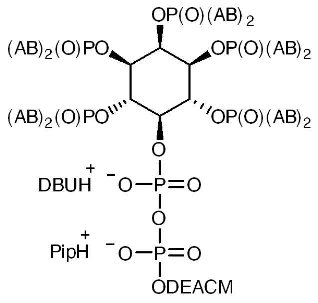

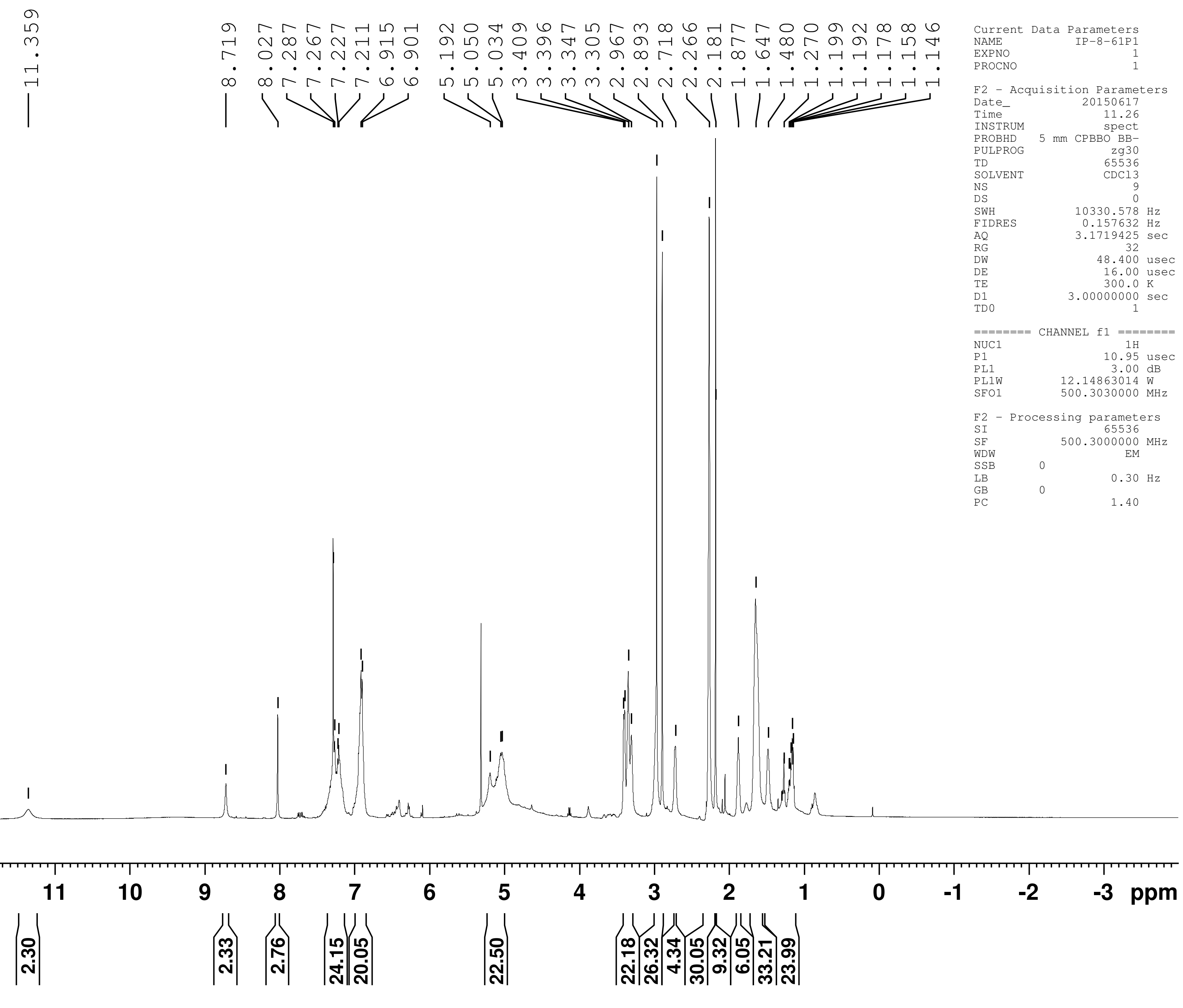

Current Data Parameters
NAME IP-8-61P1
EXPNO 1
PROCNO 1

F2 - Acquisition Parameters
Date_ 20150617
Time 11.26
INSTRUM spect
PROBHD 5 mm CPBBO BB-
PULPROG zg30
TD 65536
SOLVENT CDCl3
NS 9
DS 0
SWH 10330.578 Hz
FIDRES 0.157632 Hz
AQ 3.1719425 sec
RG 32
DW 48.400 usec
DE 16.00 usec
TE 300.0 K
D1 3.00000000 sec
TD0 1

======== CHANNEL f1 ========
NUC1 1H
P1 10.95 usec
PL1 3.00 dB
PL1W 12.14863014 W
SFO1 500.3030000 MHz

F2 - Processing parameters
SI 65536
SF 500.3000000 MHz
WDW EM
SSB 0
LB 0.30 Hz
GB 0
PC 1.40

Compound 8, 13C{1H}

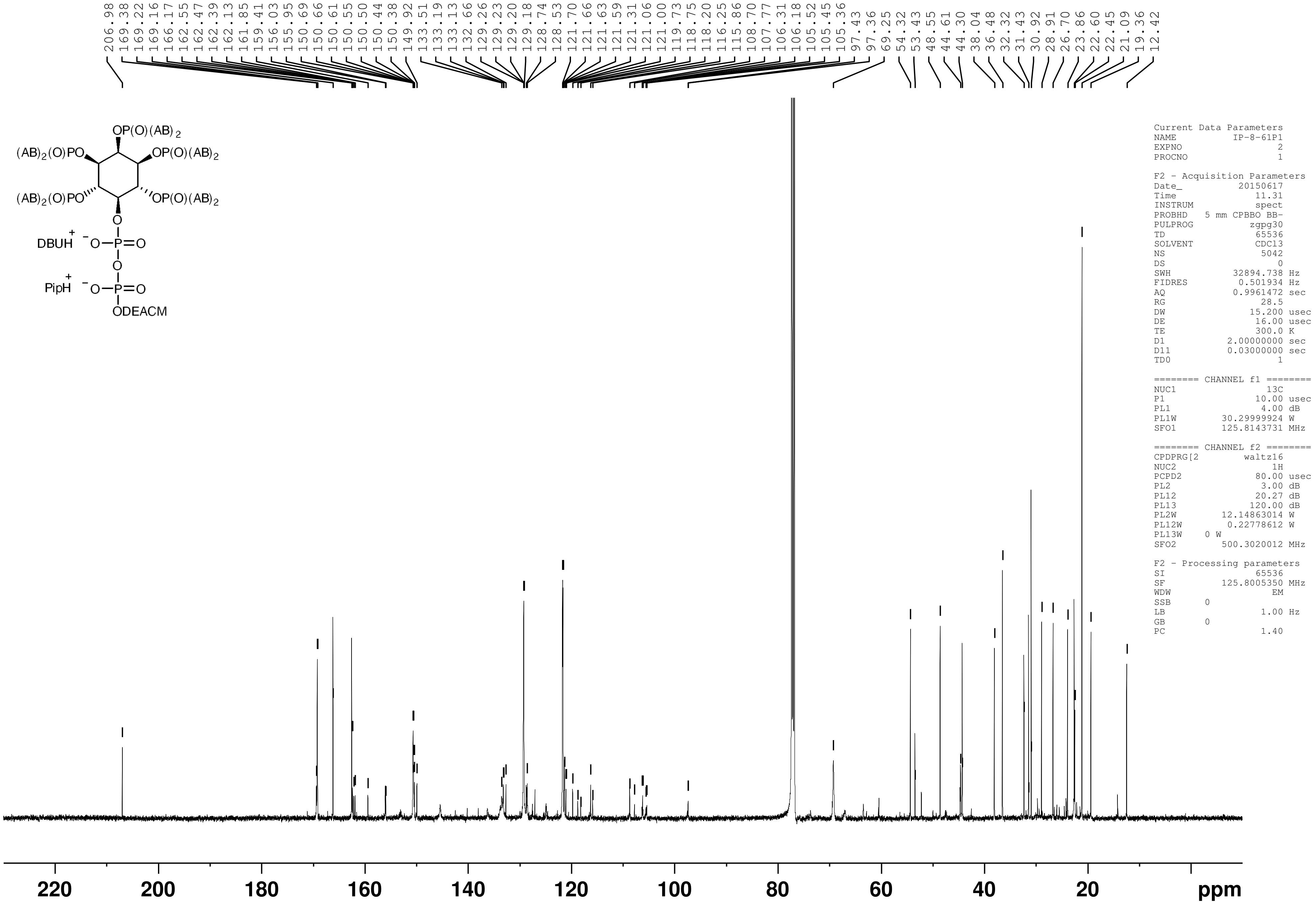

Compound 8, 31P{1H}

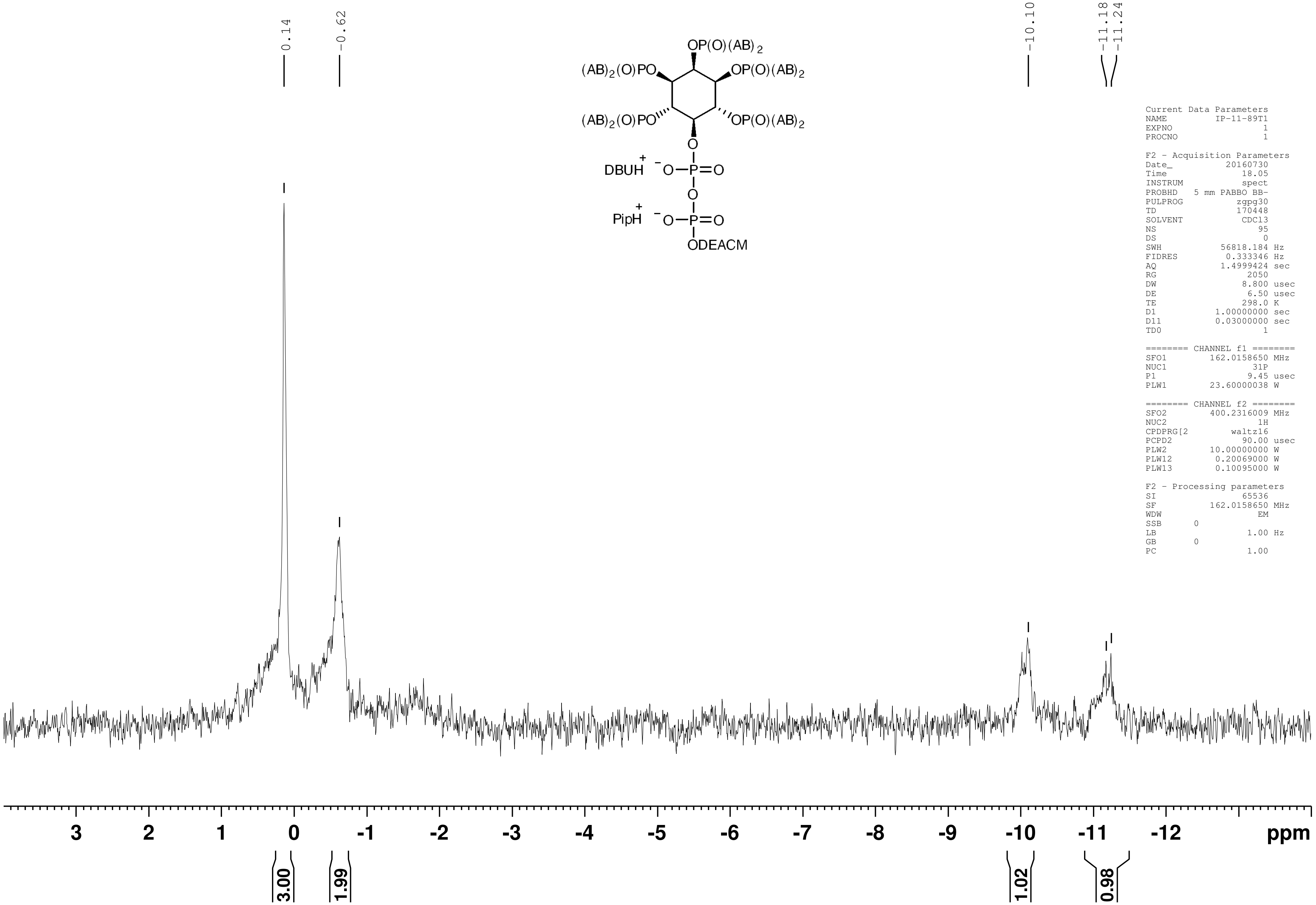


```
Current Data Parameters
NAME          IP-11-89T1
EXPNO                  1
PROCNO                 1

F2 - Acquisition Parameters
Date_           20160730
Time               18.05
INSTRUM            spect
PROBHD   5 mm PABBO BB-
PULPROG          zgpg30
TD                170448
SOLVENT            CDCl3
NS                    95
DS                     0
SWH           56818.184 Hz
FIDRES         0.333346 Hz
AQ            1.4999424 sec
RG                  2050
DW                 8.800 usec
DE                  6.50 usec
TE                 298.0 K
D1            1.00000000 sec
D11           0.03000000 sec
TD0                    1

======== CHANNEL f1 ========
SFO1         162.0158650 MHz
NUC1                 31P
P1                  9.45 usec
PLW1         23.60000038 W

======== CHANNEL f2 ========
SFO2         400.2316009 MHz
NUC2                  1H
CPDPRG[2          waltz16
PCPD2              90.00 usec
PLW2         10.00000000 W
PLW12         0.20069000 W
PLW13         0.10095000 W

F2 - Processing parameters
SI                 65536
SF           162.0158650 MHz
WDW                   EM
SSB      0
LB                  1.00 Hz
GB       0
PC                  1.00
```

Compound 8, 31P

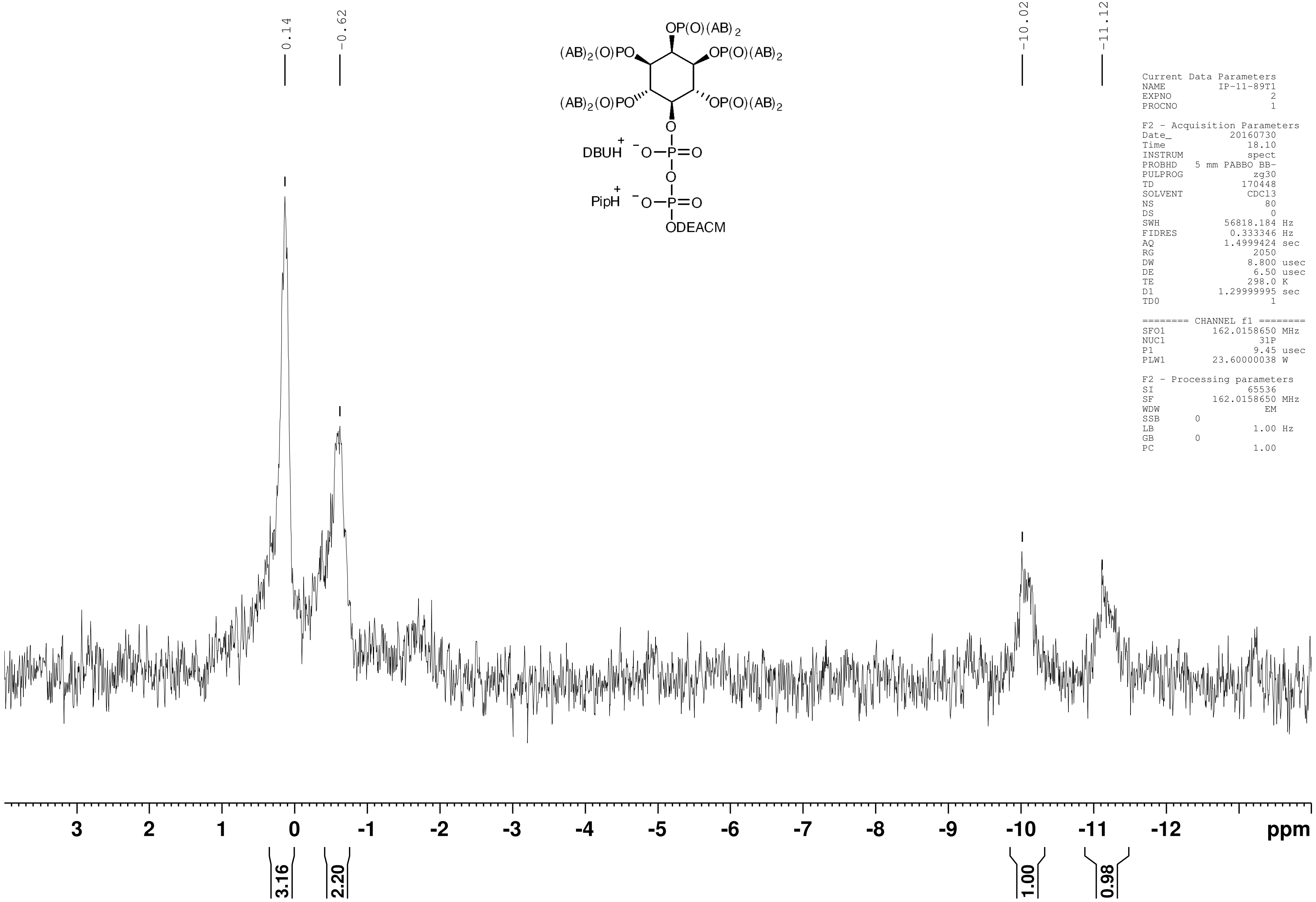


```
Current Data Parameters
NAME          IP-11-89T1
EXPNO                  2
PROCNO                 1

F2 - Acquisition Parameters
Date_           20160730
Time               18.10
INSTRUM            spect
PROBHD   5 mm PABBO BB-
PULPROG             zg30
TD                170448
SOLVENT            CDCl3
NS                    80
DS                     0
SWH           56818.184 Hz
FIDRES         0.333346 Hz
AQ            1.4999424 sec
RG                  2050
DW                 8.800 usec
DE                  6.50 usec
TE                 298.0 K
D1            1.29999995 sec
TD0                    1

======== CHANNEL f1 ========
SFO1        162.0158650 MHz
NUC1                 31P
P1                  9.45 usec
PLW1         23.60000038 W

F2 - Processing parameters
SI                 65536
SF          162.0158650 MHz
WDW                   EM
SSB      0
LB                  1.00 Hz
GB       0
PC                  1.00
```

Compound 11, 1H

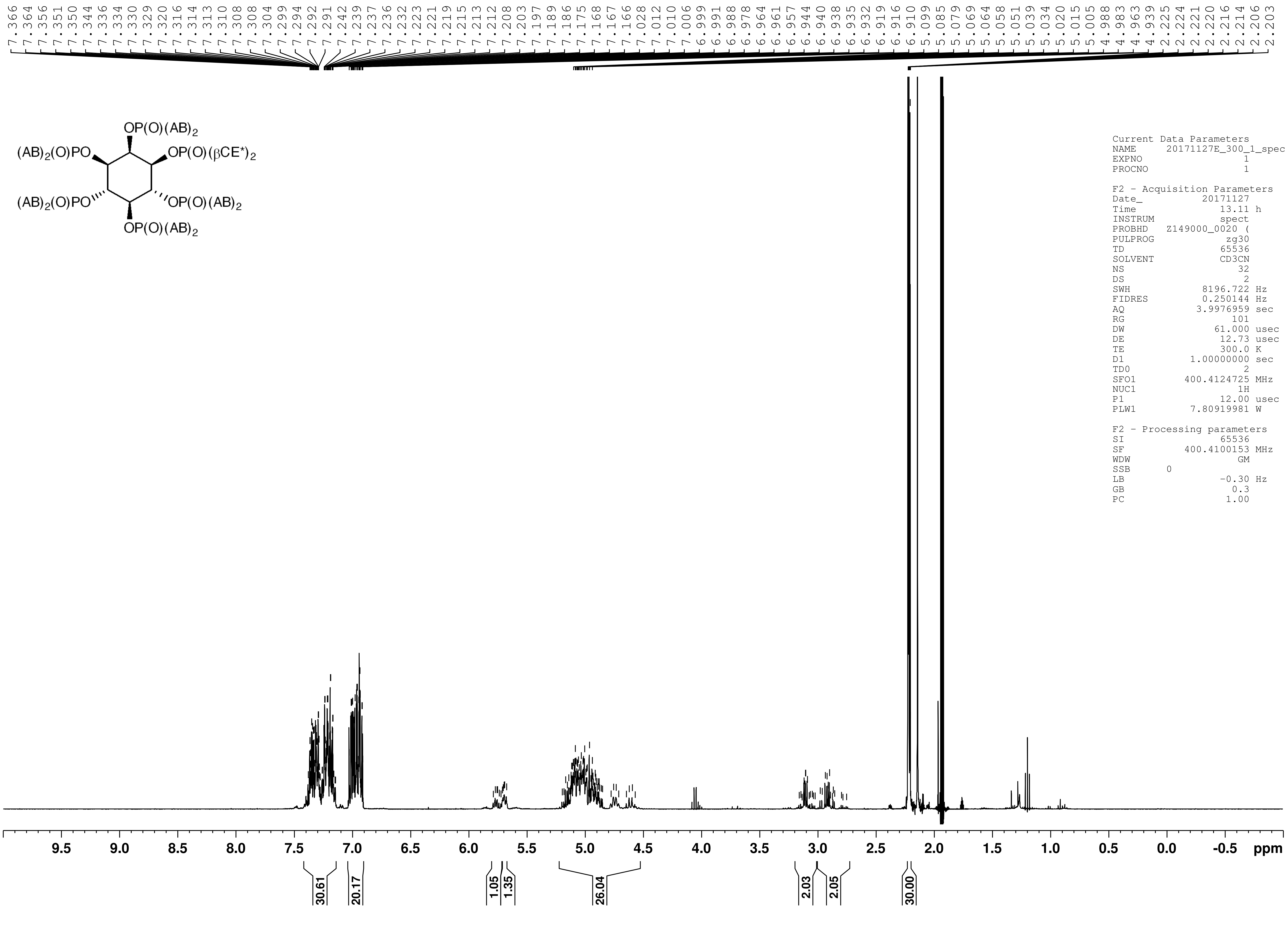


```
Current Data Parameters
NAME     20171127E_300_1_spec
EXPNO                       1
PROCNO                      1

F2 - Acquisition Parameters
Date_          20171127
Time              13.11 h
INSTRUM           spect
PROBHD   Z149000_0020 (
PULPROG            zg30
TD                65536
SOLVENT           CD3CN
NS                   32
DS                    2
SWH            8196.722 Hz
FIDRES         0.250144 Hz
AQ            3.9976959 sec
RG                  101
DW               61.000 usec
DE                12.73 usec
TE                300.0 K
D1           1.00000000 sec
TD0                   2
SFO1        400.4124725 MHz
NUC1                 1H
P1                12.00 usec
PLW1         7.80919981 W

F2 - Processing parameters
SI                65536
SF          400.4100153 MHz
WDW                  GM
SSB      0
LB                -0.30 Hz
GB                  0.3
PC                 1.00
```

Compound 11, 13C{1H}

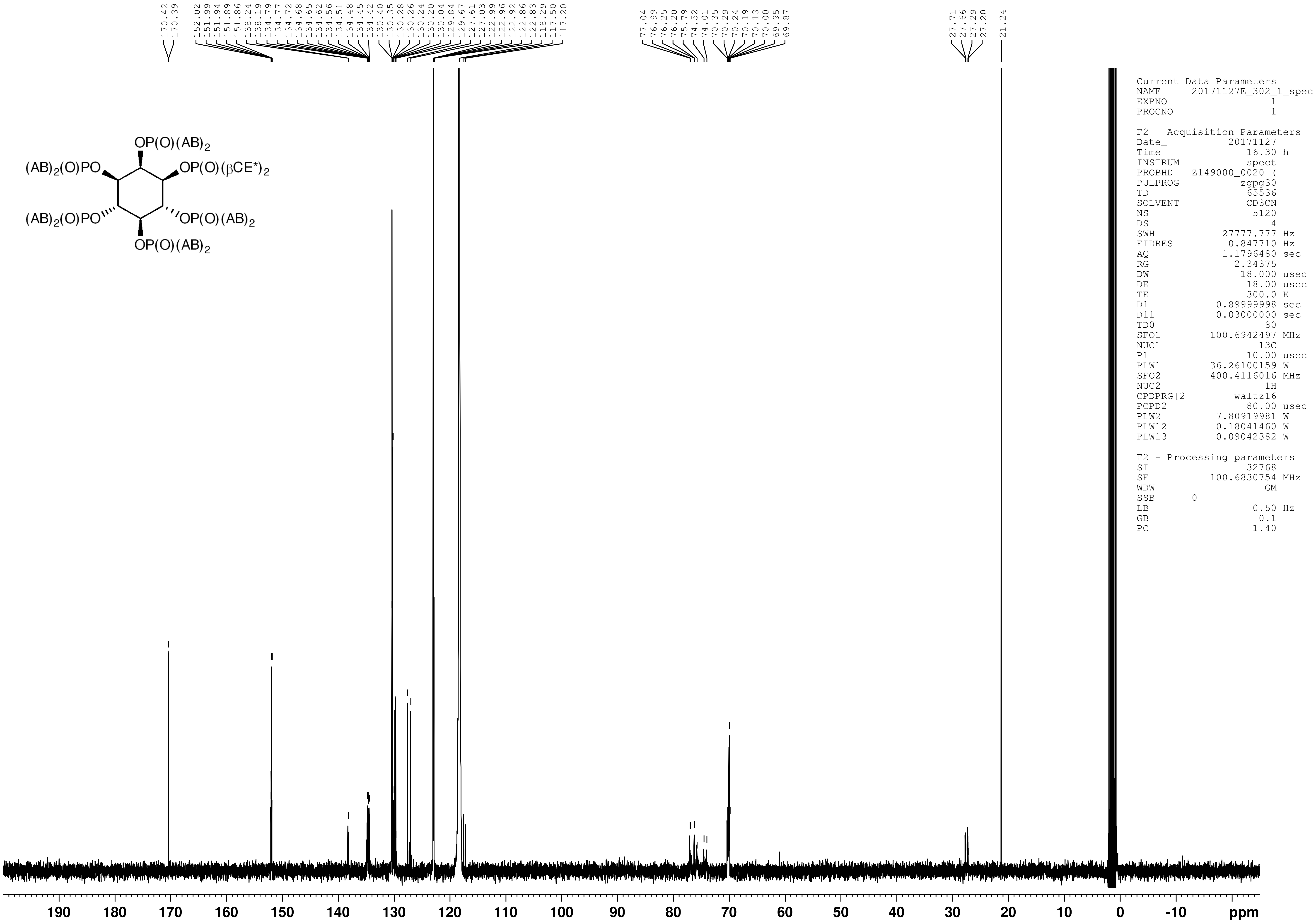

Compound 11, 31P{1H}

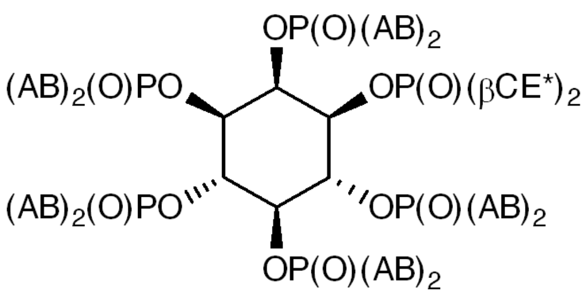


```
Current Data Parameters
NAME      20171127E_301_1_spec
EXPNO                    1
PROCNO                   1

F2 - Acquisition Parameters
Date_            20171127
Time                13.26 h
INSTRUM             spect
PROBHD   Z149000_0020 (
PULPROG            zgpg30
TD                  65536
SOLVENT             CD3CN
NS                    320
DS                      4
SWH             65789.477 Hz
FIDRES           2.007735 Hz
AQ              0.4980736 sec
RG                    101
DW                  7.600 usec
DE                  18.00 usec
TE                  300.0 K
D1             0.60000002 sec
D11            0.03000000 sec
TD0                    40
SFO1          162.0889390 MHz
NUC1                  31P
P1                  12.00 usec
PLW1          25.88400078 W
SFO2          400.4116016 MHz
NUC2                   1H
CPDPRG[2          waltz16
PCPD2               80.00 usec
PLW2           7.80919981 W
PLW12          0.18041460 W
PLW13          0.09042382 W

F2 - Processing parameters
SI                  32768
SF            162.0889390 MHz
WDW                    EM
SSB      0
LB                   1.00 Hz
GB       0
PC                   1.40
```

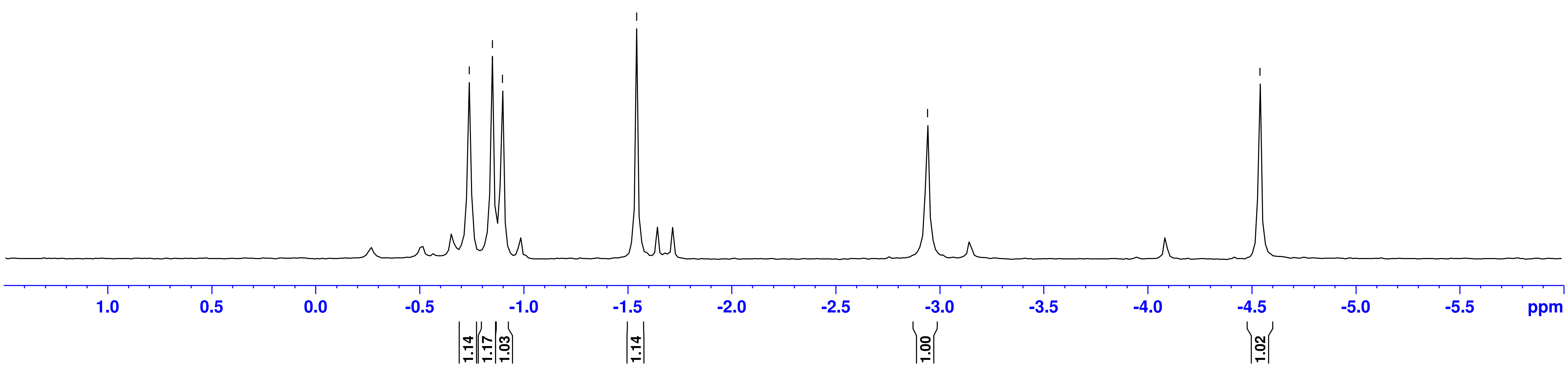

Compound 11, 31P

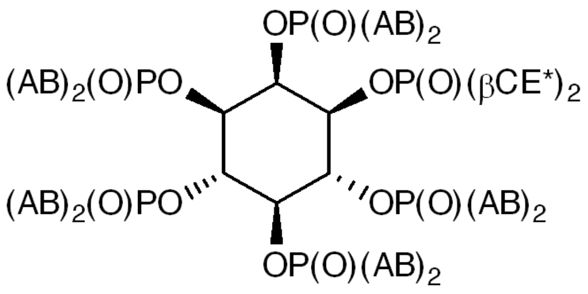

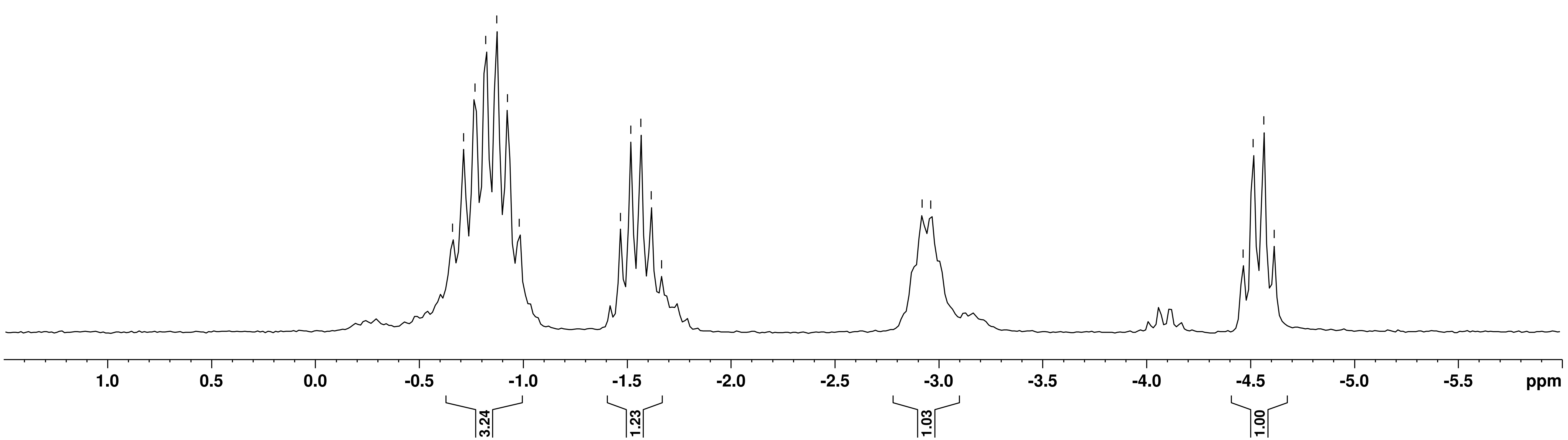

Compound dias-11, 1H

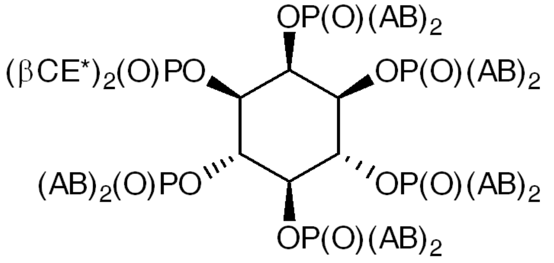

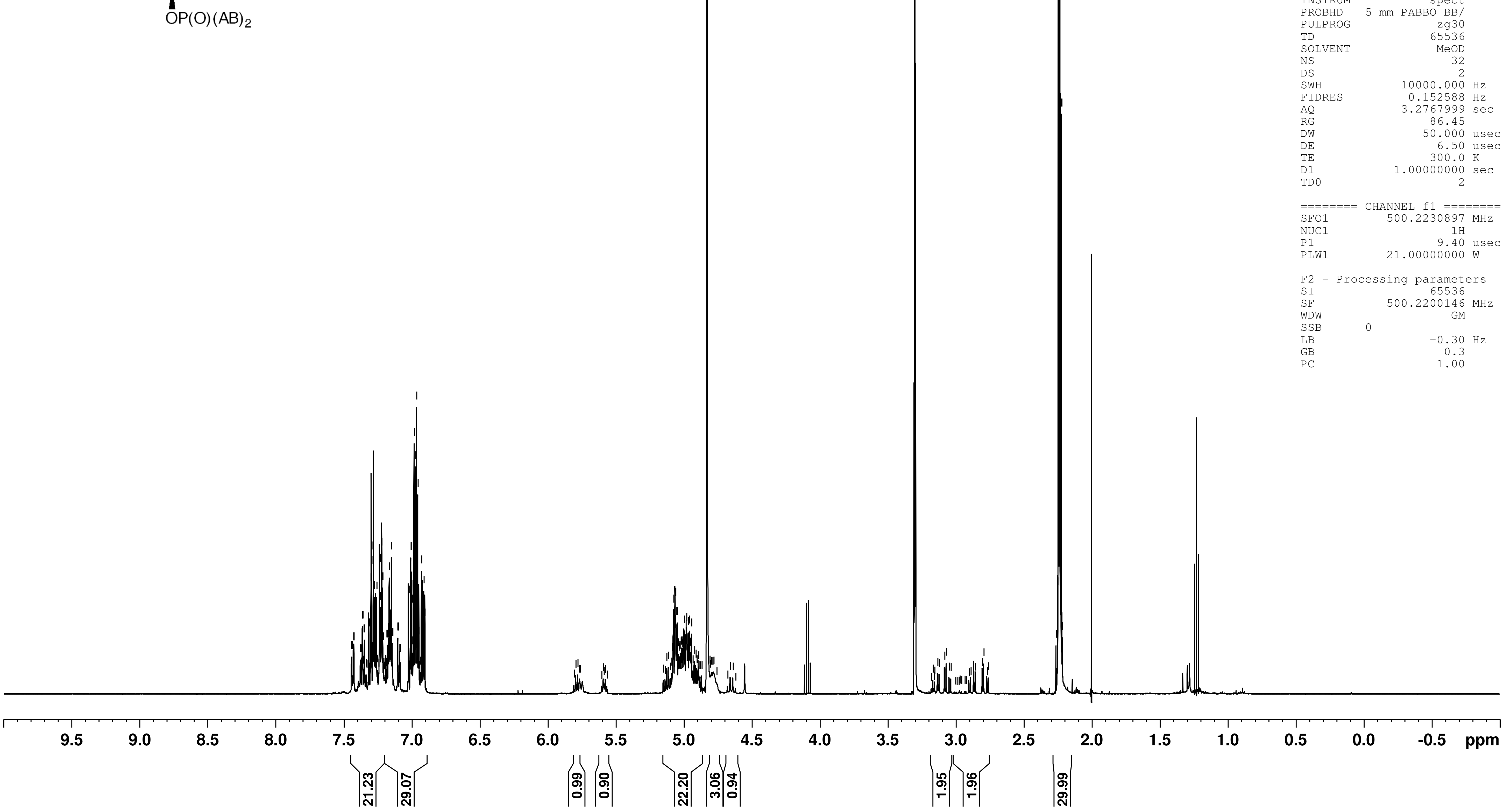


```
Current Data Parameters
NAME     20170901D_40_1_spec
EXPNO                  1
PROCNO                 1

F2 - Acquisition Parameters
Date_           20170901
Time               14.43
INSTRUM            spect
PROBHD   5 mm PABBO BB/
PULPROG             zg30
TD                 65536
SOLVENT             MeOD
NS                    32
DS                     2
SWH           10000.000 Hz
FIDRES         0.152588 Hz
AQ            3.2767999 sec
RG                 86.45
DW                50.000 usec
DE                 6.50 usec
TE                300.0 K
D1           1.00000000 sec
TD0                    2

======== CHANNEL f1 ========
SFO1        500.2230897 MHz
NUC1                  1H
P1                  9.40 usec
PLW1         21.00000000 W

F2 - Processing parameters
SI                 65536
SF           500.2200146 MHz
WDW                   GM
SSB      0
LB                 -0.30 Hz
GB                   0.3
PC                  1.00
```

Compound dias-11, 13C{1H}

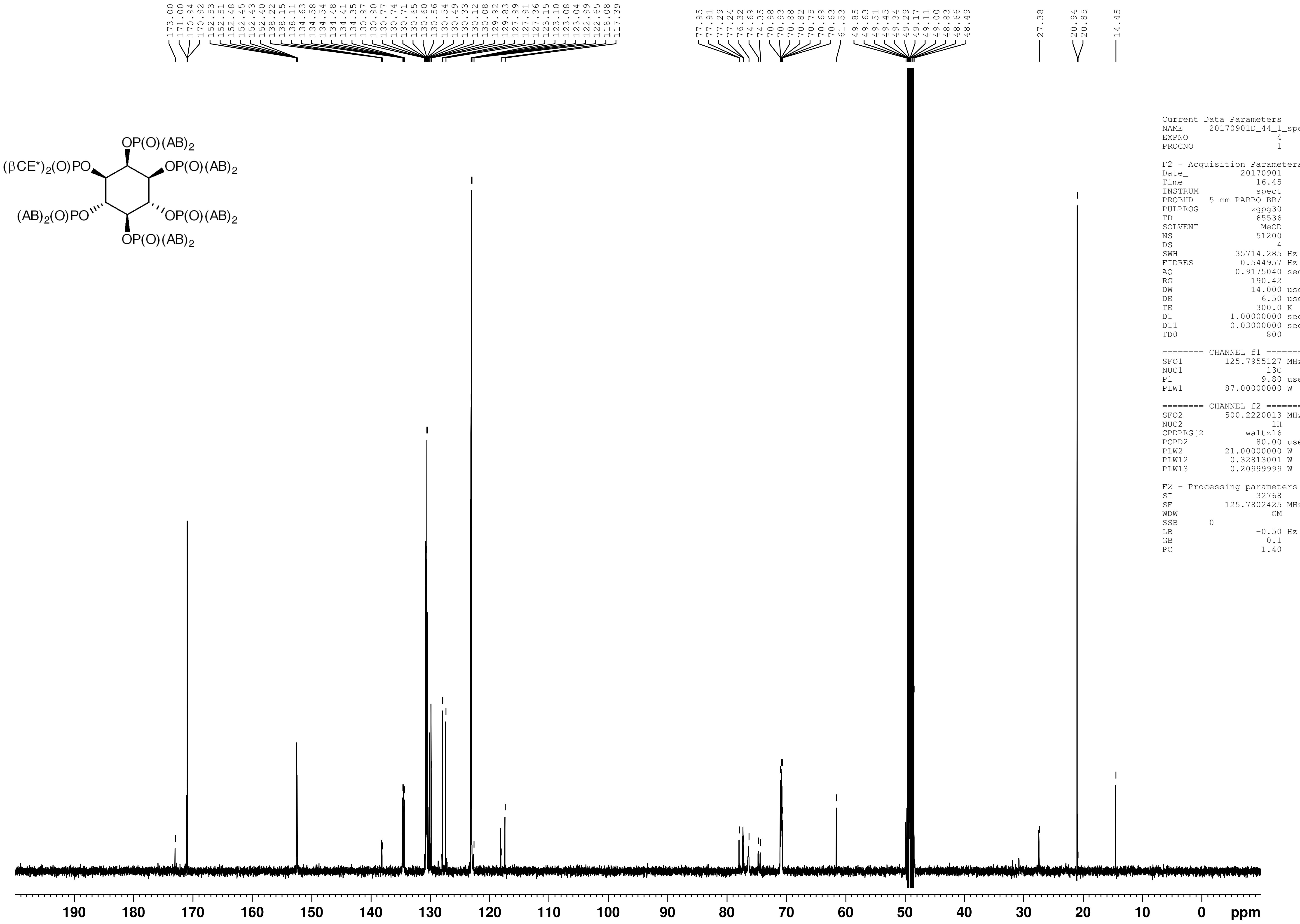


```
Current Data Parameters
NAME     20170901D_44_1_spec
EXPNO                      4
PROCNO                     1

F2 - Acquisition Parameters
Date_           20170901
Time               16.45
INSTRUM            spect
PROBHD   5 mm PABBO BB/
PULPROG          zgpg30
TD                 65536
SOLVENT             MeOD
NS                 51200
DS                     4
SWH          35714.285 Hz
FIDRES        0.544957 Hz
AQ           0.9175040 sec
RG              190.42
DW              14.000 usec
DE                6.50 usec
TE               300.0 K
D1          1.00000000 sec
D11         0.03000000 sec
TD0                800

======== CHANNEL f1 ========
SFO1        125.7955127 MHz
NUC1               13C
P1                9.80 usec
PLW1       87.00000000 W

======== CHANNEL f2 ========
SFO2        500.2220013 MHz
NUC2                1H
CPDPRG[2          waltz16
PCPD2             80.00 usec
PLW2       21.00000000 W
PLW12       0.32813001 W
PLW13       0.20999999 W

F2 - Processing parameters
SI                 32768
SF          125.7802425 MHz
WDW                   GM
SSB      0
LB                -0.50 Hz
GB                  0.1
PC                 1.40
```

Compound dias-11, 31P{1H}

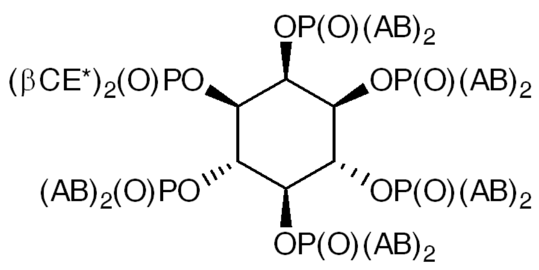

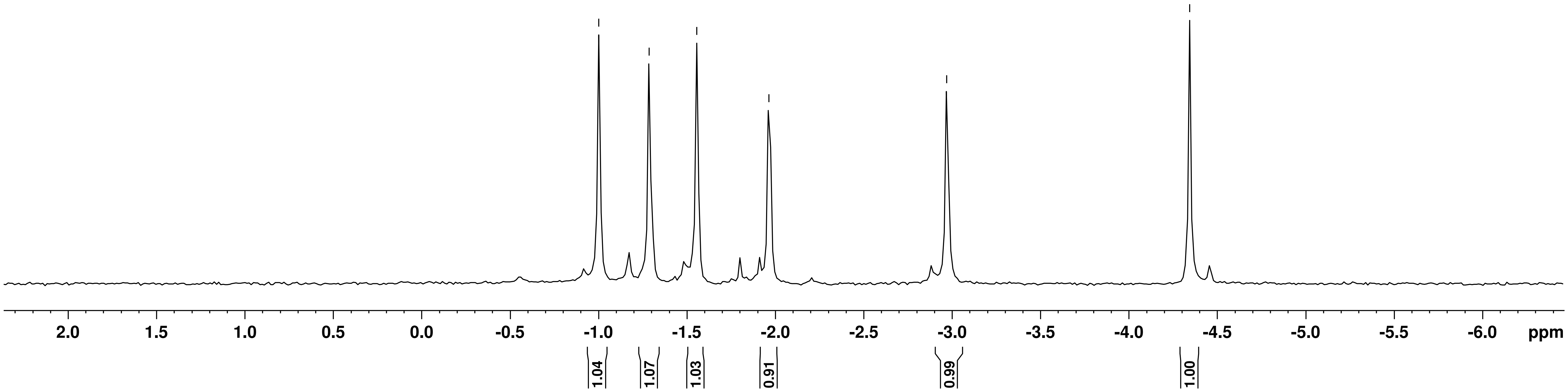


```
Current Data Parameters
NAME     20170901D_42_1_spec
EXPNO                      1
PROCNO                     1

F2 - Acquisition Parameters
Date_             20170901
Time                 14.55
INSTRUM              spect
PROBHD   5 mm PABBO BB/
PULPROG            zgpg30
TD                  65536
SOLVENT              MeOD
NS                    320
DS                      4
SWH            81521.742 Hz
FIDRES          1.243923 Hz
AQ             0.4019541 sec
RG                 190.42
DW                  6.133 usec
DE                   6.50 usec
TE                  300.2 K
D1            0.60000002 sec
D11           0.03000000 sec
TD0                    20

======== CHANNEL f1 ========
SFO1         202.4927676 MHz
NUC1                 31P
P1                  14.70 usec
PLW1         49.00000000 W

======== CHANNEL f2 ========
SFO2         500.2220009 MHz
NUC2                   1H
CPDPRG[2          waltz16
PCPD2               80.00 usec
PLW2         21.00000000 W
PLW12         0.32813001 W
PLW13         0.20999999 W

F2 - Processing parameters
SI                  32768
SF           202.4927676 MHz
WDW                    EM
SSB      0
LB                   1.00 Hz
GB       0
PC                   1.40
```

Compound 12, 31P{1H}

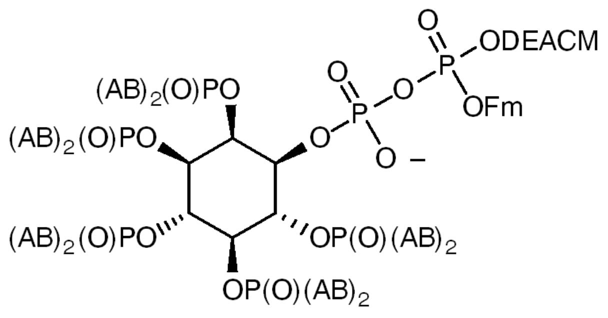


```
Current Data Parameters
NAME      Dutta,AD-137,CD3CN_SPE_spec
EXPNO                   3
PROCNO                  1

F2 - Acquisition Parameters
Date_          20170924
Time              19.40
INSTRUM           spect
PROBHD   5 mm PABBO BB-
PULPROG         zgpg30
TD                65536
SOLVENT       CD3CN_SPE
NS                  160
DS                    4
SWH          49019.609 Hz
FIDRES        0.747980 Hz
AQ           0.6684672 sec
RG                195.4
DW               10.200 usec
DE                 6.50 usec
TE                300.1 K
D1           0.40000001 sec
D11          0.03000000 sec
TD0                  10

======== CHANNEL f1 ========
SFO1        121.5009257 MHz
NUC1                31P
P1                10.40 usec
PLW1        18.00000000 W

======== CHANNEL f2 ========
SFO2        300.1312005 MHz
NUC2                 1H
CPDPRG[2        waltz16
PCPD2             90.00 usec
PLW2        10.00000000 W
PLW12        0.21186000 W
PLW13        0.17161000 W

F2 - Processing parameters
SI                32768
SF          121.4948510 MHz
WDW                  EM
SSB      0
LB                 1.00 Hz
GB       0
PC                 1.40
```

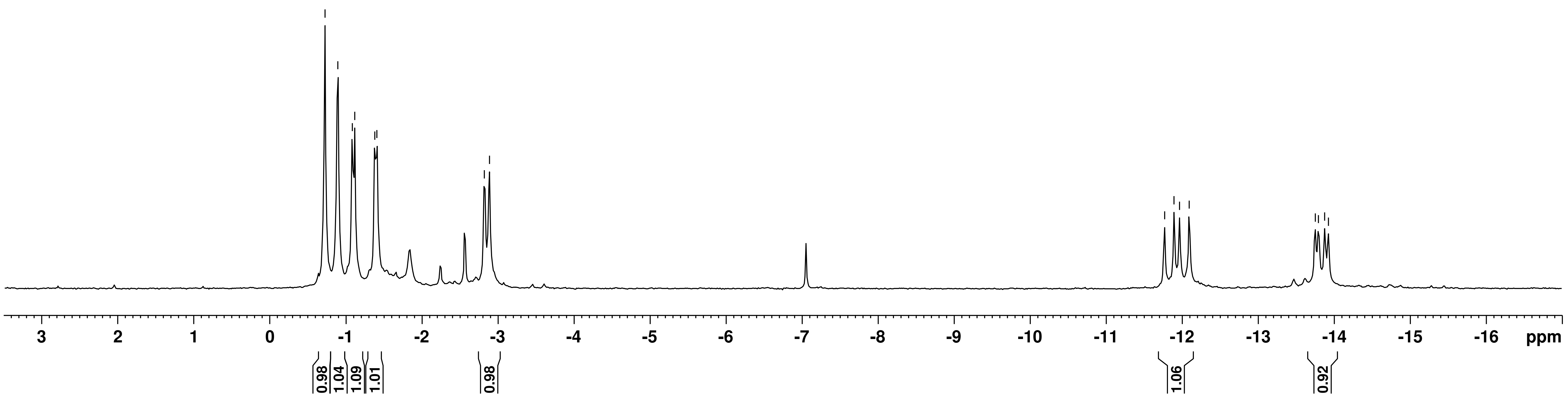

Compound dias-12, 31P{1H}

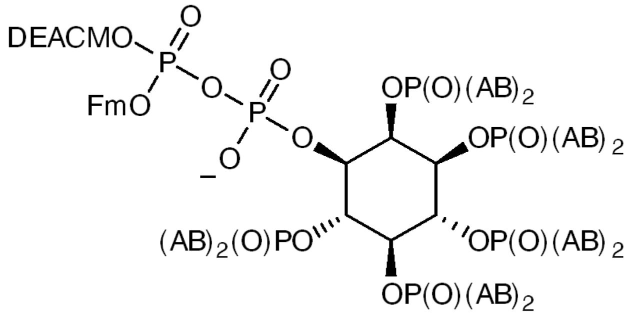


```
Current Data Parameters
NAME     20171009D_31_1_spec
EXPNO                      1
PROCNO                     1

F2 - Acquisition Parameters
Date_             20171009
Time                 12.00
INSTRUM              spect
PROBHD   5 mm PABBO BB/
PULPROG            zgpg30
TD                  65536
SOLVENT             CD3CN
NS                    640
DS                      4
SWH             81521.742 Hz
FIDRES           1.243923 Hz
AQ              0.4019541 sec
RG                 190.42
DW                  6.133 usec
DE                   6.50 usec
TE                  300.2 K
D1             0.60000002 sec
D11            0.03000000 sec
TD0                    40

======== CHANNEL f1 ========
SFO1        202.4927676 MHz
NUC1                31P
P1                14.70 usec
PLW1        49.00000000 W

======== CHANNEL f2 ========
SFO2        500.2220009 MHz
NUC2                 1H
CPDPRG[2         waltz16
PCPD2             80.00 usec
PLW2        21.00000000 W
PLW12        0.32813001 W
PLW13        0.20999999 W

F2 - Processing parameters
SI                  32768
SF            202.4927676 MHz
WDW                    EM
SSB      0
LB                   1.00 Hz
GB       0
PC                   1.40
```

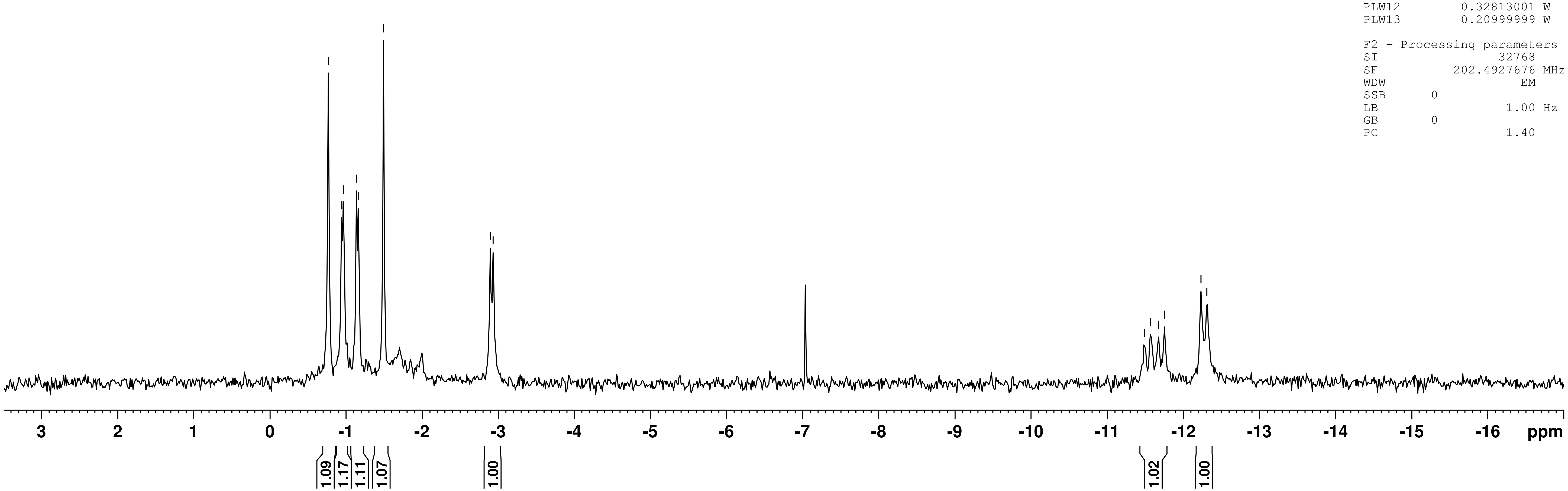

Compound ent-13, 1H

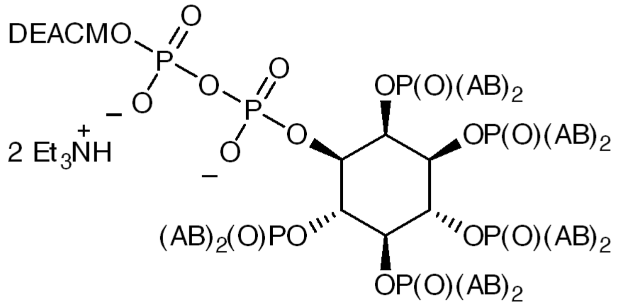

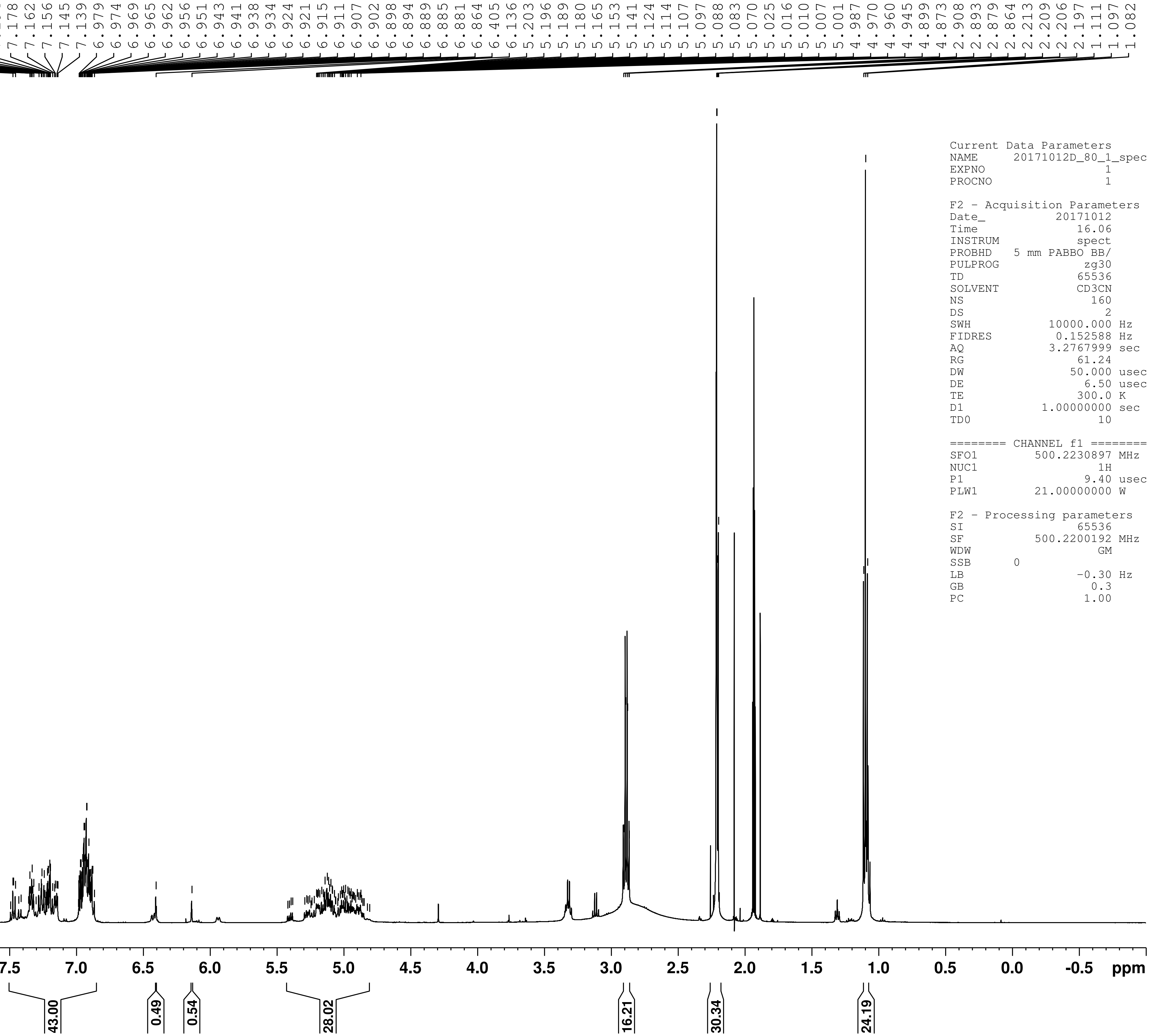


```
Current Data Parameters
NAME      20171012D_80_1_spec
EXPNO                    1
PROCNO                   1

F2 - Acquisition Parameters
Date_            20171012
Time                16.06
INSTRUM             spect
PROBHD   5 mm PABBO BB/
PULPROG              zg30
TD                  65536
SOLVENT             CD3CN
NS                    160
DS                      2
SWH             10000.000 Hz
FIDRES           0.152588 Hz
AQ              3.2767999 sec
RG                  61.24
DW                 50.000 usec
DE                   6.50 usec
TE                  300.0 K
D1            1.00000000 sec
TD0                    10

======== CHANNEL f1 ========
SFO1          500.2230897 MHz
NUC1                   1H
P1                   9.40 usec
PLW1          21.00000000 W

F2 - Processing parameters
SI                  65536
SF            500.2200192 MHz
WDW                    GM
SSB      0
LB                  -0.30 Hz
GB                    0.3
PC                   1.00
```

Compounds ent-13, 13C{1H}

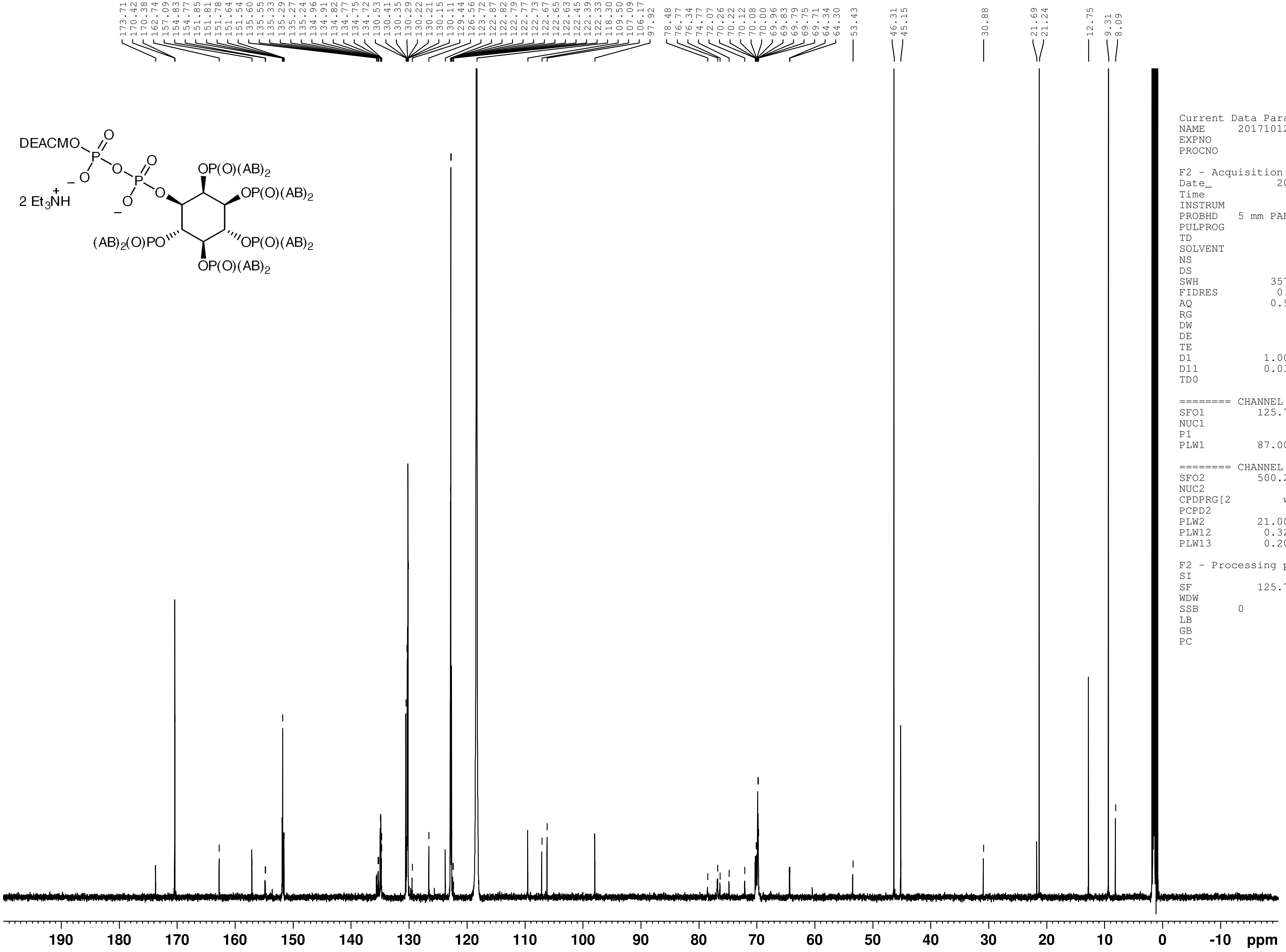


```
Current Data Parameters
NAME       20171012D_82_1_spec
EXPNO                    2
PROCNO                   1

F2 - Acquisition Parameters
Date_             20171012
Time                 16.52
INSTRUM              spect
PROBHD   5 mm PABBO BB/
PULPROG            zgpg30
TD                   65536
SOLVENT              CD3CN
NS                   25600
DS                       4
SWH              35714.285 Hz
FIDRES            0.544957 Hz
AQ               0.9175040 sec
RG                  190.42
DW                  14.000 usec
DE                    6.50 usec
TE                   300.0 K
D1              1.00000000 sec
D11             0.03000000 sec
TD0                    400

======== CHANNEL f1 ========
SFO1         125.7955127 MHz
NUC1                   13C
P1                    9.80 usec
PLW1         87.00000000 W

======== CHANNEL f2 ========
SFO2         500.2220013 MHz
NUC2                    1H
CPDPRG[2           waltz16
PCPD2                80.00 usec
PLW2         21.00000000 W
PLW12         0.32813001 W
PLW13         0.20999999 W

F2 - Processing parameters
SI                   32768
SF           125.7802958 MHz
WDW                     GM
SSB      0
LB                   -0.50 Hz
GB                     0.1
PC                    1.40
```

Compound ent-13, 31P{1H}

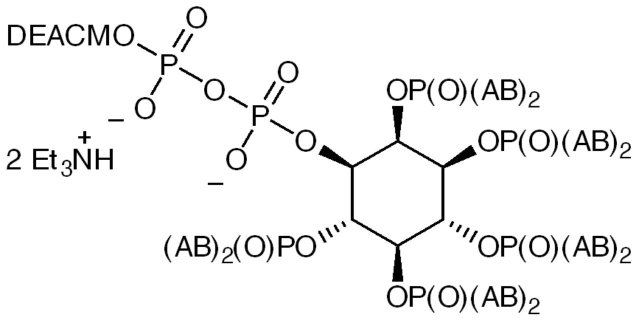

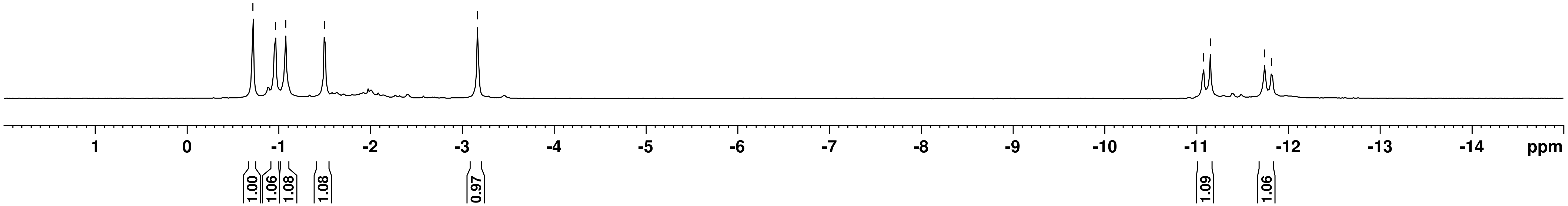


```
Current Data Parameters
NAME     20171012D_81_1_spec
EXPNO                      1
PROCNO                     1

F2 - Acquisition Parameters
Date_               20171012
Time                   16.19
INSTRUM                spect
PROBHD   5 mm PABBO BB/
PULPROG               zgpg30
TD                     65536
SOLVENT                CD3CN
NS                      1600
DS                         4
SWH                81521.742 Hz
FIDRES              1.243923 Hz
AQ                 0.4019541 sec
RG                    190.42
DW                     6.133 usec
DE                      6.50 usec
TE                     300.2 K
D1                0.60000002 sec
D11               0.03000000 sec
TD0                      100

======== CHANNEL f1 ========
SFO1             202.4927676 MHz
NUC1                     31P
P1                     14.70 usec
PLW1             49.00000000 W

======== CHANNEL f2 ========
SFO2             500.2220009 MHz
NUC2                      1H
CPDPRG[2             waltz16
PCPD2                  80.00 usec
PLW2             21.00000000 W
PLW12             0.32813001 W
PLW13             0.20999999 W

F2 - Processing parameters
SI                     32768
SF               202.4927676 MHz
WDW                       EM
SSB      0
LB                      1.00 Hz
GB       0
PC                      1.40
```

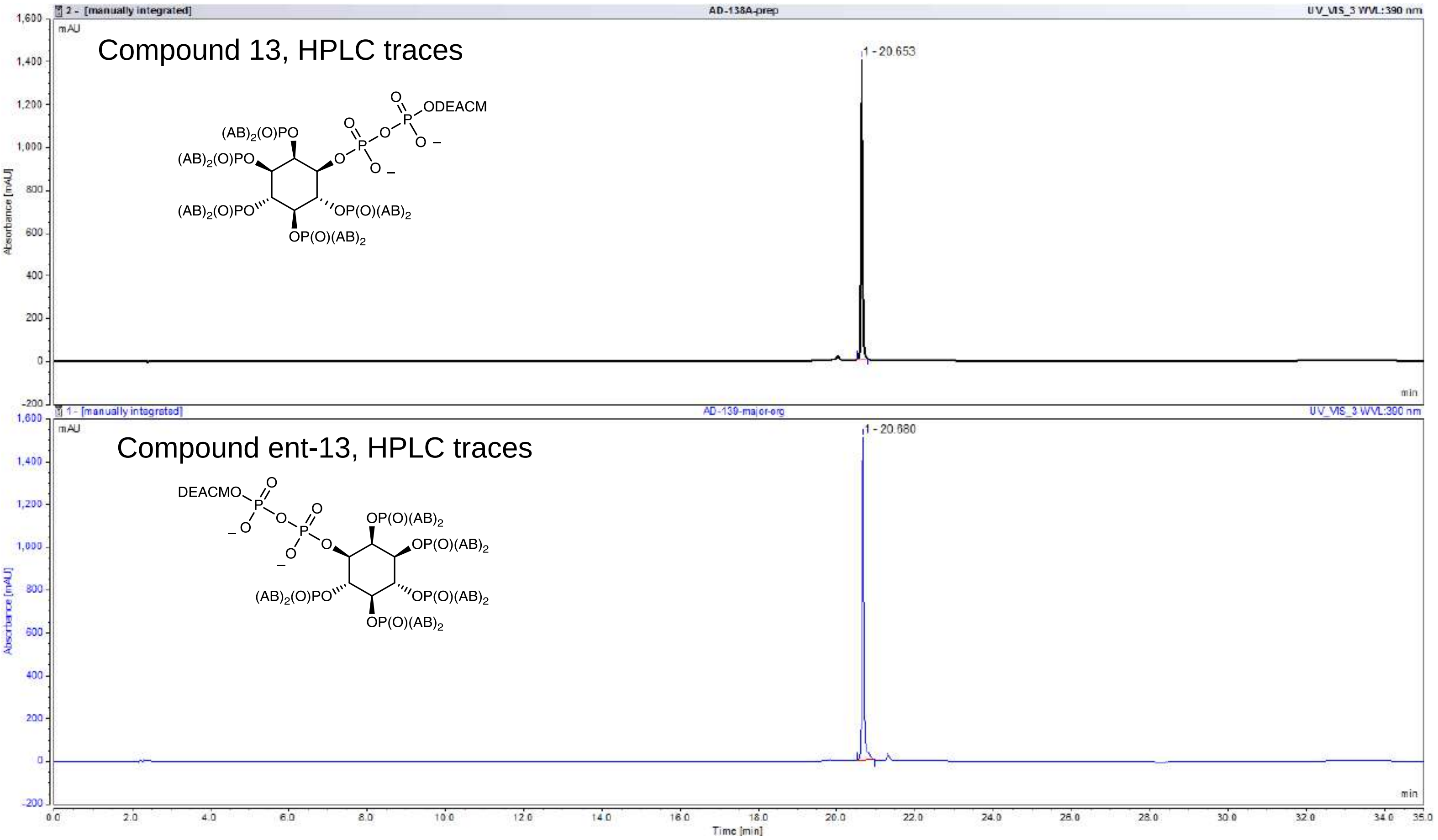
2 - [manually integrated]
AD-138A-prep
UV_VIS_3 WVL:390 nm
mAU
Compound 13, HPLC traces
1 - 20.653
ODEACM
(AB)2(O)PO
(AB)2(O)PO
(AB)2(O)PO
OP(O)(AB)2
OP(O)(AB)2
Absorbance [mAU]
min
1 - [manually integrated]
AD-139-major-org
UV_VIS_3 WVL:390 nm
mAU
Compound ent-13, HPLC traces
1 - 20.680
DEACMO
OP(O)(AB)2
OP(O)(AB)2
(AB)2(O)PO
OP(O)(AB)2
OP(O)(AB)2
Absorbance [mAU]
min
Time [min]

Compound ent-14, 1H

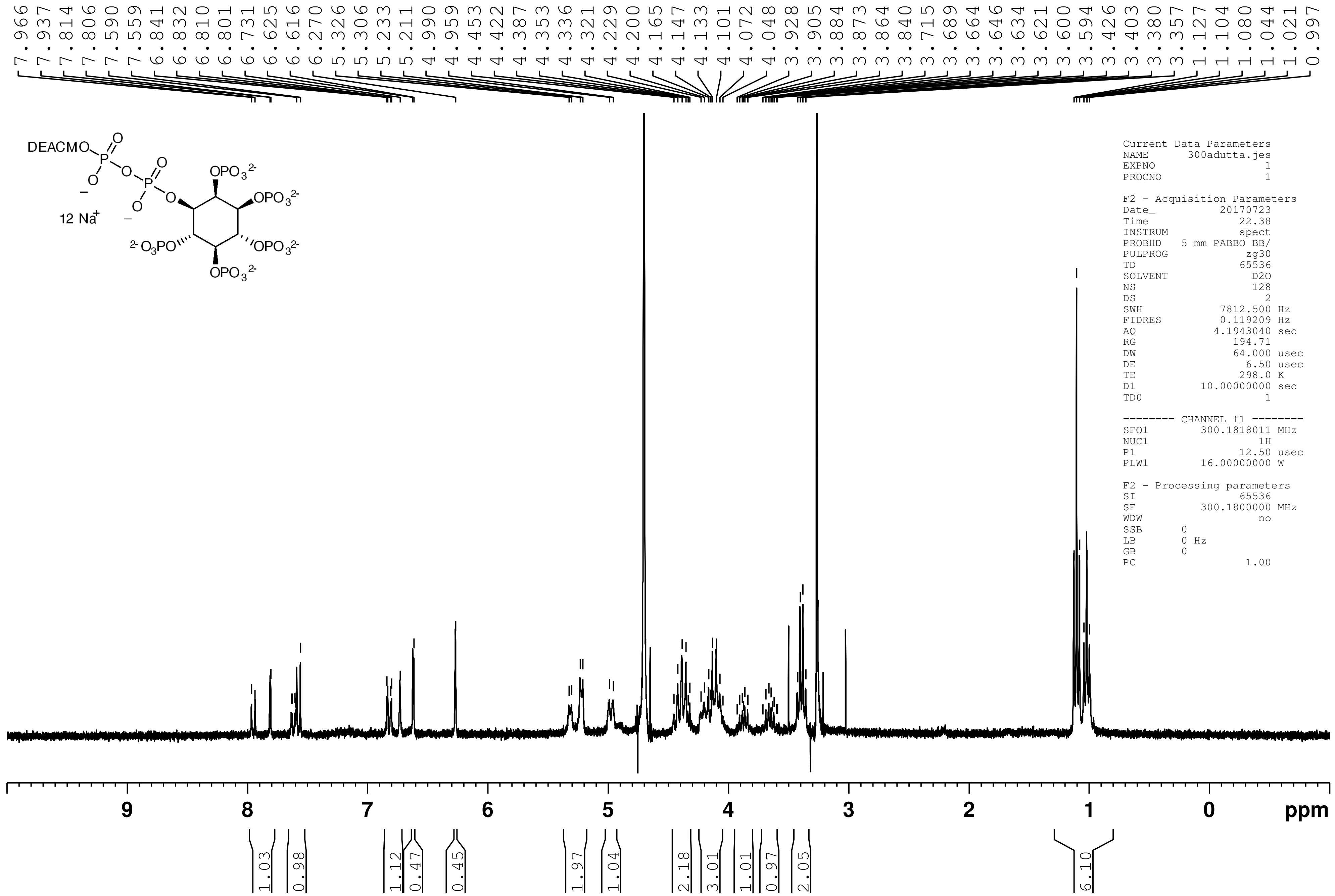

Compound ent-14, 31P{1H}

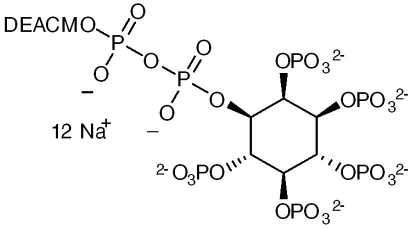

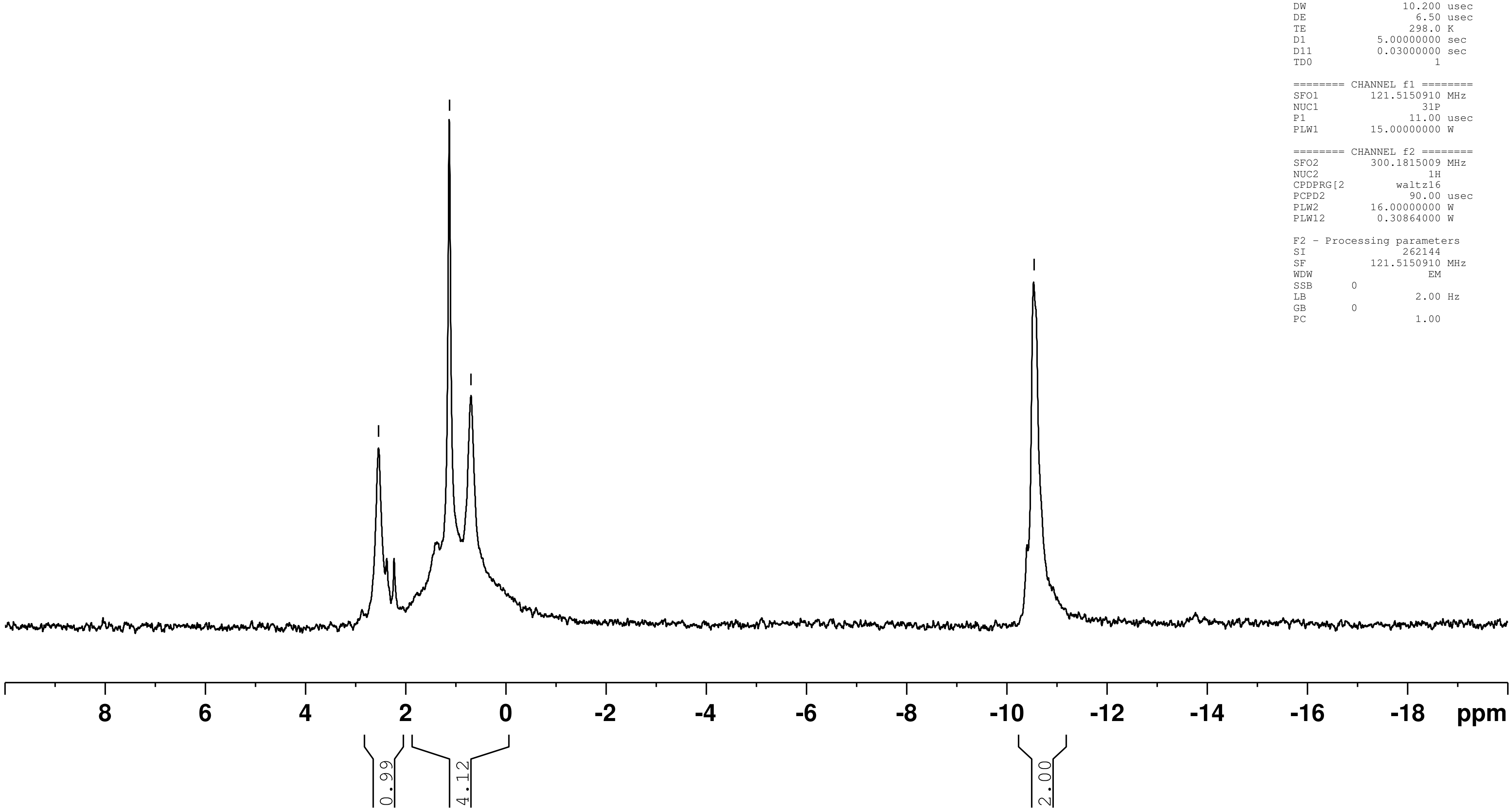


```
Current Data Parameters
NAME      300adutta.jes
EXPNO                 2
PROCNO                1

F2 - Acquisition Parameters
Date_          20170724
Time               8.04
INSTRUM           spect
PROBHD   5 mm PABBO BB/
PULPROG          zgig30
TD               262144
SOLVENT             D2O
NS                 5470
DS                    8
SWH           49019.609 Hz
FIDRES         0.186995 Hz
AQ            2.6738689 sec
RG               194.71
DW               10.200 usec
DE                 6.50 usec
TE                298.0 K
D1           5.00000000 sec
D11          0.03000000 sec
TD0                   1

======== CHANNEL f1 ========
SFO1        121.5150910 MHz
NUC1                31P
P1                11.00 usec
PLW1        15.00000000 W

======== CHANNEL f2 ========
SFO2        300.1815009 MHz
NUC2                 1H
CPDPRG[2        waltz16
PCPD2             90.00 usec
PLW2        16.00000000 W
PLW12        0.30864000 W

F2 - Processing parameters
SI               262144
SF          121.5150910 MHz
WDW                  EM
SSB      0
LB                 2.00 Hz
GB       0
PC                 1.00
```

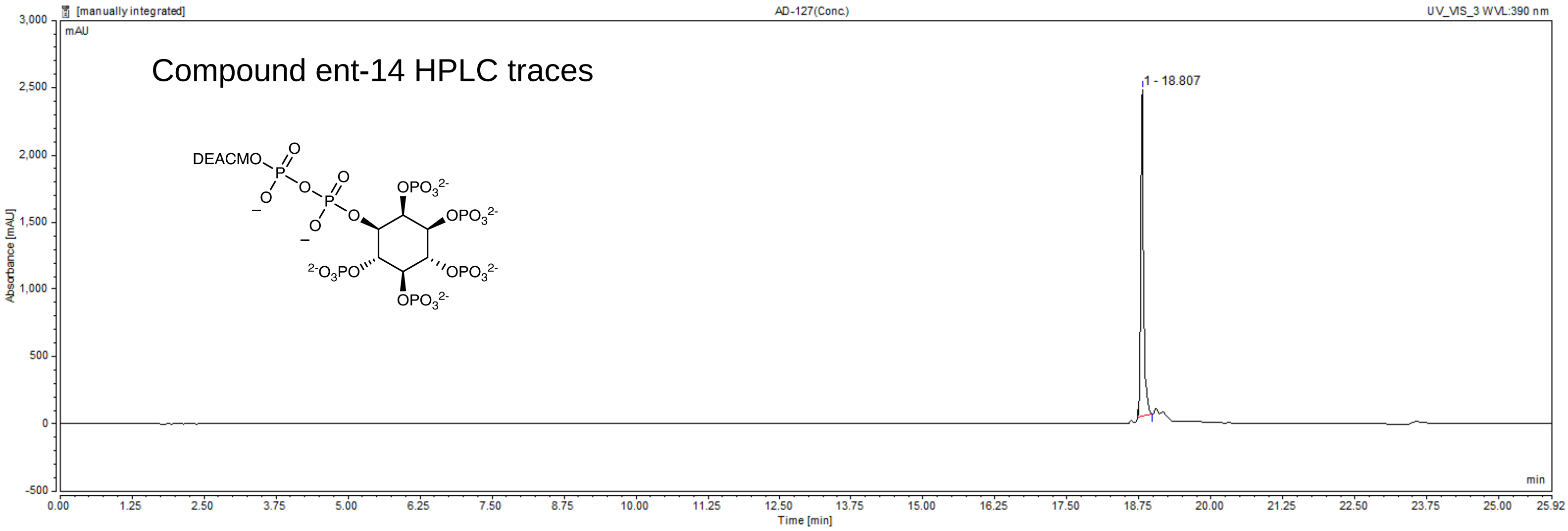
[manually integrated]
AD-127(Conc.)
UV_VIS_3 WVL:390 nm
mAU
Compound ent-14 HPLC traces
DEACMO
1 - 18.807
Absorbance [mAU]
Time [min]
min

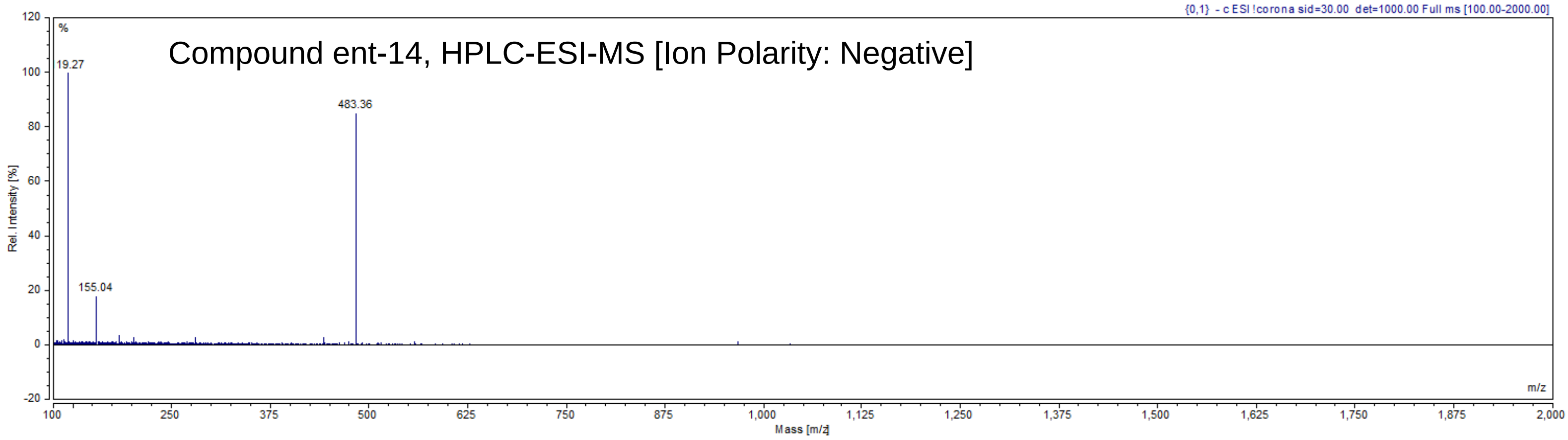
{0,1} - c ESI !corona sid=30.00 det=1000.00 Full ms [100.00-2000.00]
%
Compound ent-14, HPLC-ESI-MS [Ion Polarity: Negative]
19.27
483.36
155.04
Rel. Intensity [%]
Mass [m/z]
m/z

Compound 15, 1H

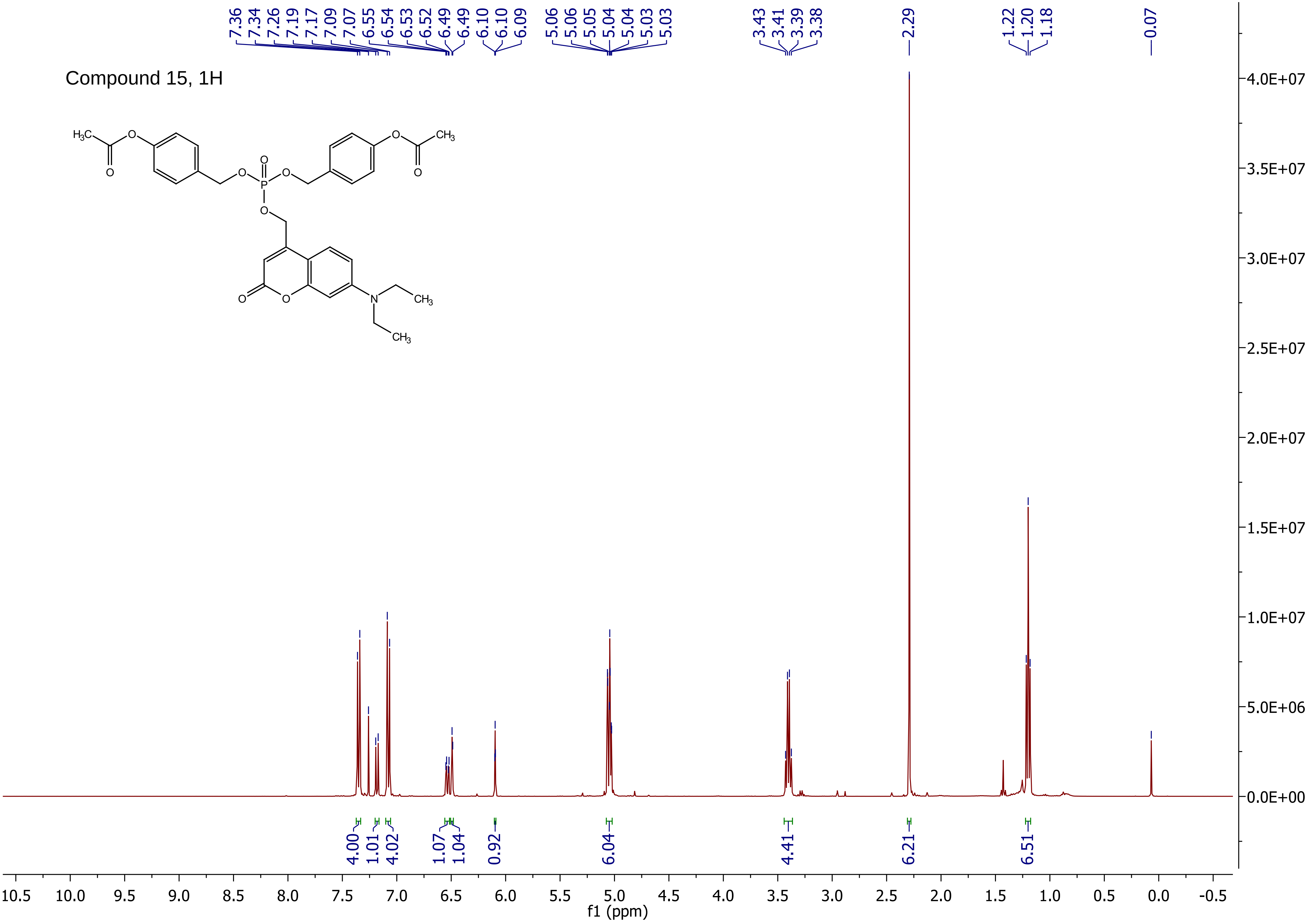

Compound 15, 31P{1H}

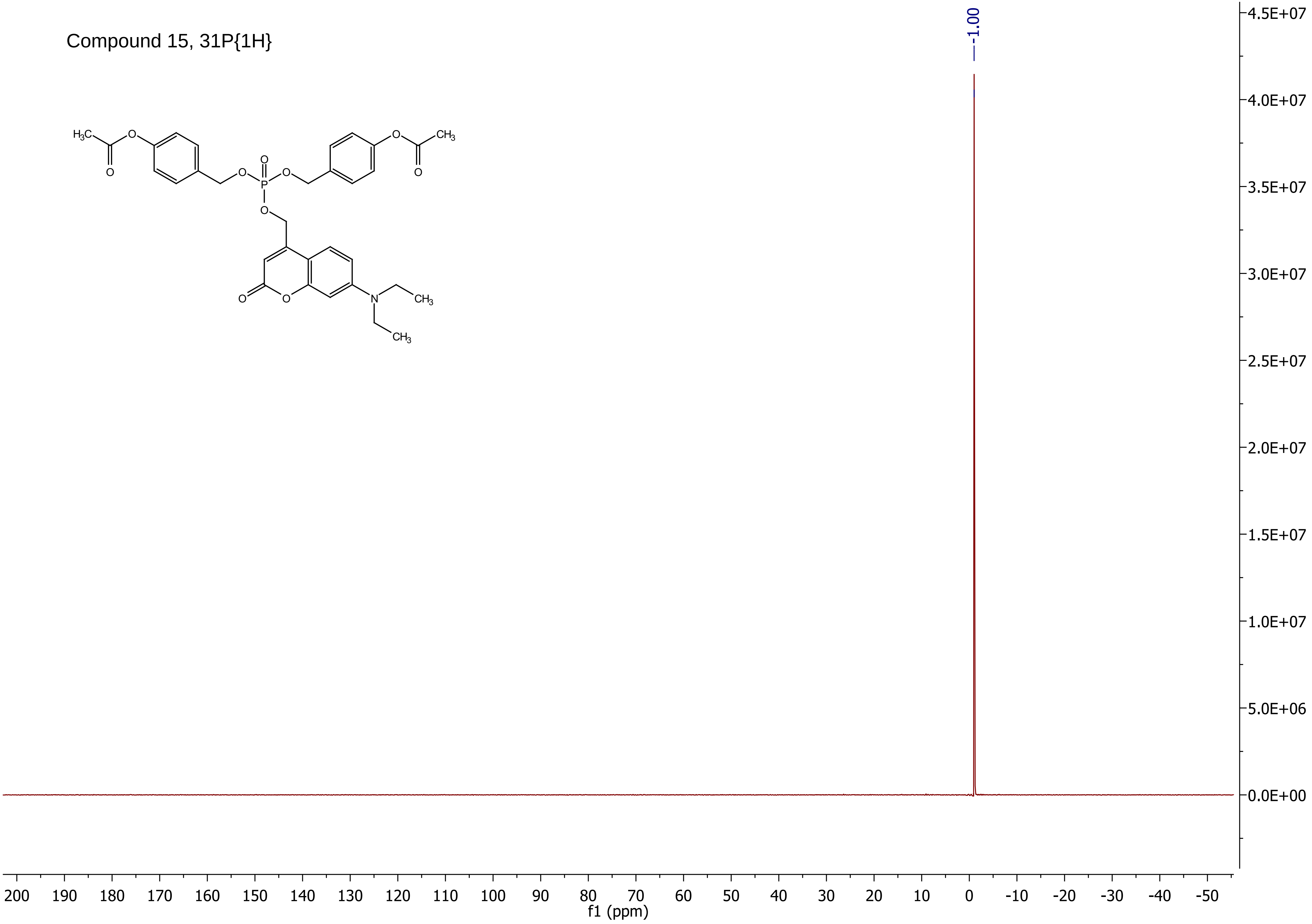

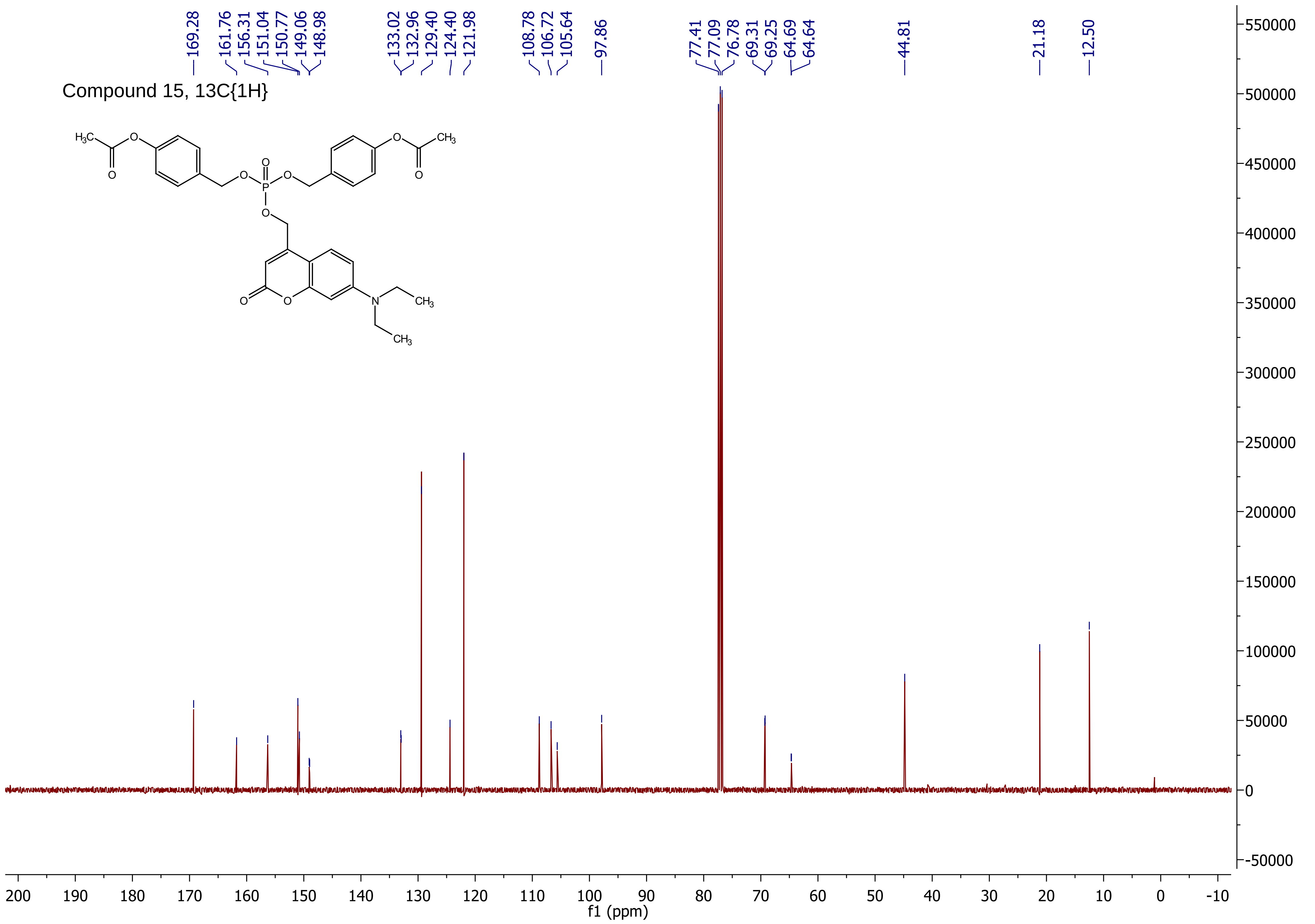

Compound 15, 13C{1H}
169.28
161.76
156.31
151.04
150.77
149.06
148.98
133.02
132.96
129.40
124.40
121.98
108.78
106.72
105.64
97.86
77.41
77.09
76.78
69.31
69.25
64.69
64.64
44.81
21.18
12.50
f1 (ppm)
